# Immune cell infiltration-related clinical diagnostic model for Ankylosing Spondylitis

**DOI:** 10.3389/fgene.2022.949882

**Published:** 2022-09-05

**Authors:** Chenxing Zhou, Tuo Liang, Jie Jiang, Zide Zhang, Jiarui Chen, Tianyou Chen, Liyi Chen, Xuhua Sun, ShengSheng Huang, Jichong Zhu, Shaofeng Wu, Xinli Zhan, Chong Liu

**Affiliations:** Department of Spine and Osteopathy Ward, The First Affiliated Hospital of Guangxi Medical University, Nanning, Guangxi, China

**Keywords:** Ankylosing Spondylitis, immune infiltration, monocytes, neutrophils, nomogram, diagnosis

## Abstract

**Background:** The pathogenesis and diagnosis of Ankylosing Spondylitis (AS) has remained uncertain due to several reasons, including the lack of studies on the local and systemic immune response in AS. To construct a clinical diagnostic model, this study identified the micro RNA-messenger RNA (miRNA-mRNA) interaction network and immune cell infiltration-related hub genes associated with AS.

**Materials and Methods:** Total RNA was extracted and purified from the interspinous ligament tissue samples of three patients with AS and three patients without AS; miRNA and mRNA microarrays were constructed using the extracted RNA. Bioinformatic tools were used to construct an miRNA-mRNA network, identify hub genes, and analyze immune infiltration associated with AS. Next, we collected the blood samples and clinical characteristics of 359 patients (197 with AS and 162 without AS). On the basis of the clinical characteristics and results of the routine blood tests, we selected immune-related cells whose numbers were significantly different in patients with AS and patients without AS. Univariate and multivariate logistic regression analysis was performed to construct a nomogram. Immunohistochemistry staining analysis was utilized to verify the differentially expression of *LYN* in AS and controls.

**Results:** A total of 225 differentially expressed miRNAs (DE miRNAs) and 406 differentially expressed mRNAs (DE mRNAs) were identified from the microarray. We selected 15 DE miRNAs and 38 DE mRNAs to construct a miRNA-mRNA network. The expression of *LYN*, an immune-related gene, correlated with the counts of monocytes, neutrophils, and dendritic cells. Based on the independent predictive factors of sex, age, and counts of monocytes, neutrophils, and white blood cells, a nomogram was established. Receiver operating characteristic (ROC) analysis was performed to evaluate the nomogram, with a C-index of 0.835 and AUC of 0.855.

**Conclusion:** *LYN*, an immune-related hub gene, correlated with immune cell infiltration in patients with AS. In addition, the counts of monocytes and neutrophils were the independent diagnostic factors for AS. If verified in future studies, a diagnostic model based on these findings may be used to predict AS effectively.

## Introduction

Axial spondyloarthritis (SpA) is a chronic inflammatory rheumatic disease characterized by a progressive inflammatory course. The global prevalence rate of SpA is 0.9%–1.4% (Taurog et al., 2016). The disease mainly affects the axial skeleton, and the symptoms include inflammatory back pain, spinal mobility limitation, and peripheral and extra-articular manifestations (van der Heijde et al., 2019). Sacroiliitis in patients with SpA is diagnosed by using radiographic and non-radiographic techniques. Radiographic sacroiliitis positive SpA is known as Ankylosing Spondylitis (AS) (van der Heijde et al., 2017; Ward et al., 2019). Because this disease mainly affects the productive population of society, i.e., young adults (Miceli-Richard et al., 2000), AS poses a considerable burden to patients and society (Krüger et al., 2018).

Biologic therapies are effective in the early treatment of AS (Rudwaleit et al., 2009), thereby reducing the joint fusion rate in long-term follow-ups (Haroon et al., 2013). However, chronic back pain is common in society, with the cause remaining mostly uncertain. Moreover, only a minority of patients with this symptom have a diagnosis of AS. Therefore, it can be challenging to diagnose AS on the basis of the symptom of chronic back pain. Consequently, the early treatment of AS is delayed, and the average delay in diagnosis can be 6–10 years (Feldtkeller et al., 2003; Reed et al., 2008; Ogdie et al., 2019). Therefore, it is necessary to construct an effective clinical prediction model for AS.

In the last few years, developments in bioinformatics have supported the growth of molecular biology research. Bioinformatics can be used to study molecular biology mechanisms, and some special biological markers can be identified and used as targets for disease diagnosis and treatment. A microarray is an effective tool for the analysis of pathological tissues. The analysis of differentially expressed genes (DEGs) in a microarray is common in molecular biology research. MicroRNAs (miRNAs) include approximately 20–24 nucleotides (Ogdie et al., 2019; Zhao et al., 2019) belonging to a series of small noncoding RNAs. miRNAs can regulate the degradation and translation of target mRNAs. Recently, a study reported that the potential pathogenic mechanism of AS can be attributed to the abnormal regulation of miRNAs that occurs as a consequence of differentially expressed (DE) mRNAs (Qin et al., 2019).

**FIGURE 1:**
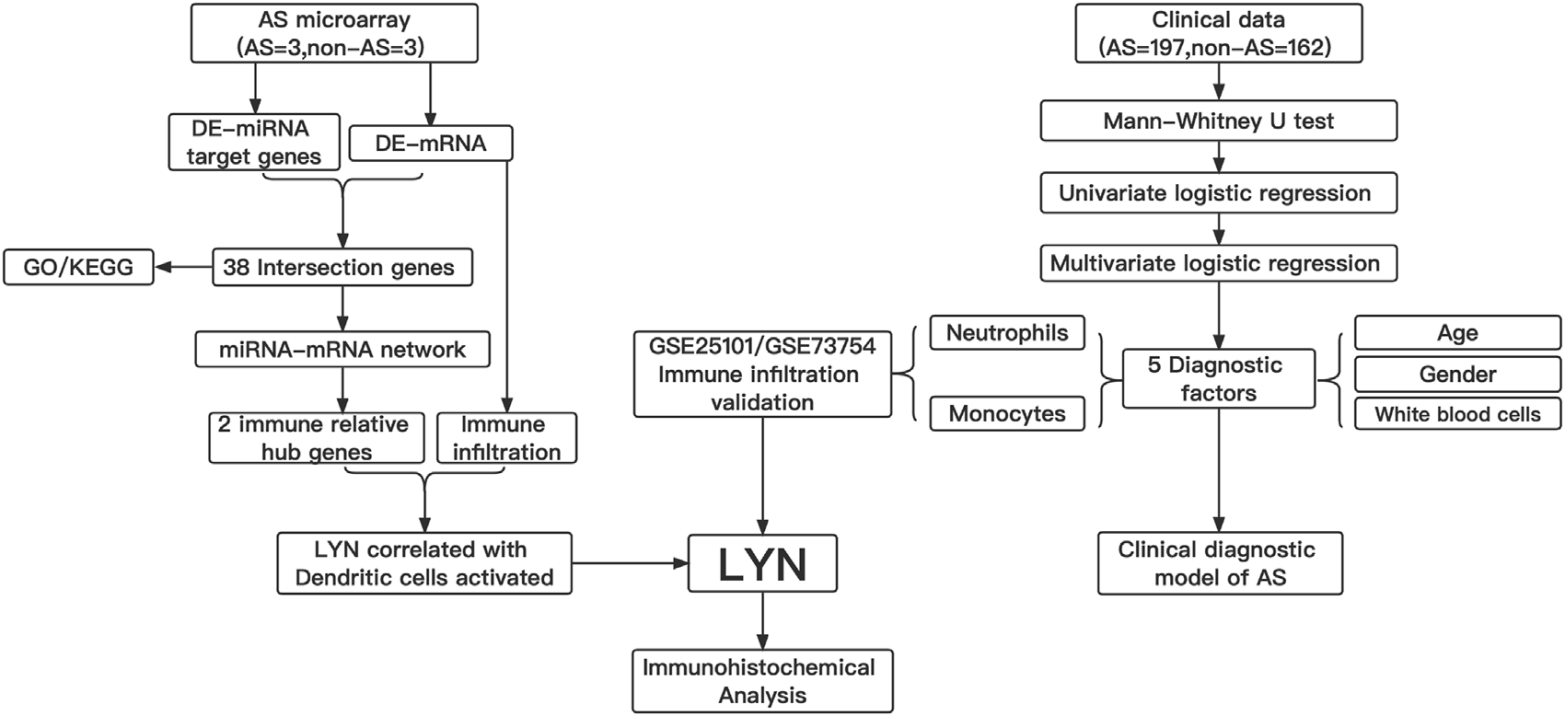
Workflow gram of this study. DE-miRNAs, different expression of miRNAs; DE-mRNAs, different expression of mRNAs ; GO, gene ontology; KEGG, kyoto encyclopedia of genes and genomes; *LYN*, *LYN* Proto-Oncogene, Src Family Tyrosine Kinase.

In this study, we constructed an miRNA-mRNA network associated with AS by using an interspinous ligament microarray. Subsequently, immune-related hub genes were identified from the constructed network. Immune cell infiltration analysis of the interspinous ligament tissue was performed by using CIBERSORT. We further analyzed the clinical data and determined neutrophils and monocytes to be the independent predictive factors for AS, which was verified by performing an immune cell infiltration search on the GEO database. Finally, an AS clinical diagnosis model visualized by nomogram was constructed, and the diagnostic effectiveness of this model was verified. Figure 1 shows the workflow of this study

## Materials and methods

### Patients

Participants signed informed consent forms. This study was approved by the Ethics Committee of The First Affiliated Hospital of Guangxi Medical University.

We collected the interspinous ligament tissue samples from three patients with AS and three patients without AS during surgical procedures; the samples were collected from September 2015 to June 2019. Loss of horizontal vision without compensation, disabling kyphosis, painful spinal pseudarthrosis, or Andersson lesion were the surgery indications (Zochling et al., 2006). Patients without AS did not have any systemic inflammation, including SpA; however, they presented with the symptoms of thoracic spine cord compression or lumbar spinal stenosis.

Blood samples from patients with AS and patients without AS were collected during hospitalization and ambulatory treatment. Supplementary Table S1 shows the clinical characteristics of the patient. We collected 197 samples from patients with AS and 162 samples from patients without AS from September 2015 to June 2019. Loss of horizontal vision without compensation, disabling kyphosis, painful spinal pseudarthrosis, or Andersson lesion were the diagnostic indications for AS. Patients without AS did not have systemic infection and inflammation, including SpA.

### miRNA and mRNA microarray

We selected the interspinous ligament tissue samples of six patients (three patients with AS and three patients without AS). Total RNA from the interspinous ligament tissue samples of patients were extracted and purified using mirVana™ miRNA Isolation Kit (catalog number: AM1561, Ambion, Austin, TX, United States), as per the manufacturer’s instructions; the RNA integrity number (RIN) of the extracted RNA was determined by using an Agilent Bioanalyzer 2100 (Agilent Technologies, Santa Clara, CA, United States). Total RNA from the interspinous ligament tissue samples of patients with AS was amplified and labeled by Low Input Quick Amp Labeling Kit, one-color (catalog number: 5190–2305, Agilent Technologies, Santa Clara, CA, United States), as per the manufacturer’s instructions. Labeled cRNA was purified by RNeasy mini kit (catalog number: 74106, QIAGEN, GmBH, Germany). Each slide was hybridized with 1.65 µg Cy3-labeled cRNA using Gene Expression Hybridization Kit (catalog number: 5188–5242, Agilent Technologies, Santa Clara, CA, United States) in a hybridization oven (catalog number: G2545A, Agilent Technologies, Santa Clara, CA, United States), as per the manufacturer’s instructions. After 17 h of hybridization, slides were washed in staining dishes (catalog number: 121, Thermo Shandon, Waltham, MA, United States) with Gene Expression Wash Buffer Kit (catalog number: 5188–5327, Agilent Technologies, Santa Clara, CA, United States), as per the manufacturer’s instructions. Slides were scanned by using Agilent Microarray Scanner (catalog number: G2565CA, Agilent technologies, Santa Clara, CA, United States) with the default settings of dye channel: green, scan resolution = 3 μm, PMT 100%, 20 bit. Feature Extraction software 10.7 was used to extract the data (Agilent technologies, Santa Clara, CA, United States), and quantile normalization of the raw data was performed by using limma packages in R.

### Data download

We searched the GEO database [“Ankylosing Spondylitis” (MeSH Terms) and mRNA (All Fields) AND “*Homo sapiens*” (Organism) AND “Expression profiling by array” (Filter)] from the date of its inception to August 2021. The inclusion criterion was: mRNAs from healthy people or patients with AS. In the end, the following two datasets were chosen for analysis: GSE 25101 based on Illumina HumanHT-12 V3.0 expression beadchip and GSE 73754 based on GPL10558 Illumina HumanHT-12 V4.0 expression beadchip. Normalization, background correction, and log2 transformation were performed by using the R package “affy” from the Bioconductor project. Analysis of the DE mRNAs was performed using the Student’s t-test. A *p* value of <0.05 was set as the criterion for selecting the DE mRNAs. We obtained 2,483 immune-related genes data from immProt database (https://www.immport.org/shared/).

### Identification of miRNA-mRNA network and hub genes from microarray

The DE mRNA and miRNA obtained by using microarray were subjected to Student’s t-test. A *p* value of <0.05 was set as the criterion for identifying DE mRNAs and miRNAs. The target genes of the DE miRNAs were predicted by the bioinformatics software FunRich and compared with the DE mRNAs identified by microarray. We used a Venn diagram to present the intersection genes and identified the overlapping mRNAs. miRNAs are negatively correlated with the expression of target genes. This fact was used to identify the DE mRNAs. An miRNA-mRNA network was constructed by using Cytoscape v3.8.0 (Szklarczyk et al., 2015; Doncheva et al., 2019) and visualized by using a Sankey diagram (R package “ggalluvial” and “ggplot2”). Hub genes were identified by using Cytoscape v3.8.0.

### Gene ontology and kyoto encyclopedia of genes and genomes pathway enrichment analyses of intersection genes

GO (Gaudet and Dessimoz, 2017) and KEGG (Kanehisa et al., 2017) pathway enrichment analyses were performed by using R packages, including clusterProfifiler, org. Hs.eg.db, enrichplot, and ggplot2 (Yu et al., 2012). A *p* value of <0.05 was set as the cut-off value. GO enrichment analysis included three main categories: Cellular Component (CC), Biological Process (BP), and Molecular Function (MF).

### Estimation of immune infiltration-related cells and genes

To evaluate immune infiltration in patients with AS, we analyzed the immune cell composition of the microarray and GEO dataset by using CIBERSORT. The microarray was constructed using RNA from interspinous ligament tissue samples, whereas the GEO database included data from blood samples. CIBERSORT (Newman et al., 2015) is a bioinformatic software that can evaluate the composition of immune cells and express the immune cell components in a matrix. The total value of the immune cell compositions for each sample in the microarray and GEO dataset was 100%. To explore the correlation between immune cells and AS, we constructed a correlation heat map. Finally, we used the statistically significant immune cell differences in AS (*p* < 0.05) for subsequent analyses.

### Statistical analysis of clinical data

We performed statistical analyses by using SPSS V.22.0. Clinical data were represented as mean (SD) and median (P25, P75). According to the type of data, Student’s t-test, Mann-Whitney U test, or chi-square test was performed to compare the differences between the two groups. The level of significance was set at *p* < 0.05. Subsequently, univariate and multivariate logistic regression analysis was performed to identify the independent predictive factors. The results of logistic regression were compared, and a nomogram was constructed. The performance of the nomogram and the independent predictive factors was assessed by using receiver operating characteristic (ROC) curves (SPSS V.22.0) and C-index calibration (“RMS” package). A *p* value of <0.05 was considered to be a statistically significant difference.

### Immunohistochemistry

The interspinous ligaments of five AS patients with kyphosis who underwent surgery in the First Clinical Affiliated Hospital of Guangxi Medical University were used as the experimental group. The interspinous ligaments of three spinal fracture who underwent surgery were used as the control group. Immunohistochemistry was used to compare the differences in *LYN* expression between AS group and control group. Baseline information of the patients can be found in Supplementary Table S10. Antibodies specific to *LYN* for specific staining were purchased from the Bioss Antibodies (http://www.bioss.com.cn/prolook_03.asp?id=AF08169606002674&pro37=1), dilution ratio of 1:200. The interspinous ligament tissue was isolated and preserved in formalin solution for 10 min. We obtained all 16 immunohistochemical sections with completed staining after laboratory operations such as wax sealing, sectioning, antigen repair, antibody hybridization, color development, and sealing of the tissue. The specimens were observed under an inverted microscope, and the images of the AS group and normal control group were captured respectively. We used ImageJ software to assess the positivity of all immunohistochemical images. IBM SPSS Statistics 22.0 were utilized to performed statistical analysis of the positivity of *LYN* in the AS and normal control groups by using independent samples t-test.

## Results

### Differentially expressed miRNAs and mRNAs in patients with Ankylosing Spondylitis and without Ankylosing Spondylitis

**FIGURE 2:**
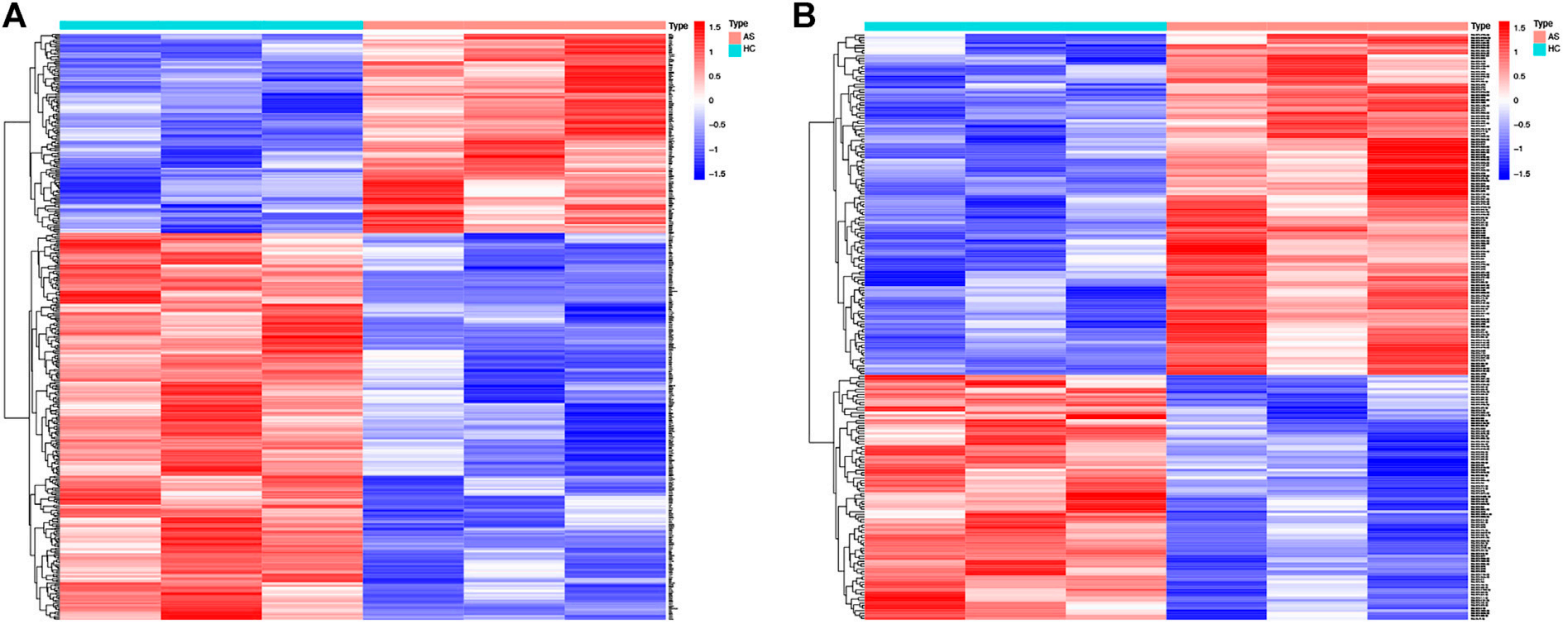
Heat map of DE-mRNAs and DE-miRNAs from the AS microarray. **(A)** Heat map of 406 DE-mRNAs. Red part of the heat map indicates upregulated mRNAs, blue part of the heat map indicates downregulated mRNAs. **(B)** Heat map of 225 DE-miRNAs. Red part of the heat map indicates upregulated miRNAs, blue part of the heat map indicates downregulated miRNAs.

A total of 406 DE mRNAs, including 138 upregulated and 268 downregulated mRNAs, were obtained from interspinous ligament tissue samples of three patients with AS and three patients without AS. A Heatmap of DE mRNAs is provided in Figure 2A. We identified 225 DE miRNAs (131 upregulated and 94 downregulated) from interspinous ligament tissue samples of three patients with AS and three patients without AS by using the Student’s t-test. A Heatmap of DE miRNAs are provided in Figure 2B. Clinical characteristics of three patients with AS and three patients without AS are included in Supplementary Table 1. The list of DE miRNAs and DE mRNAs are included in Supplementary Tables S2, S3, respectively.

### Identification of miRNA-mRNA network

**FIGURE 3:**
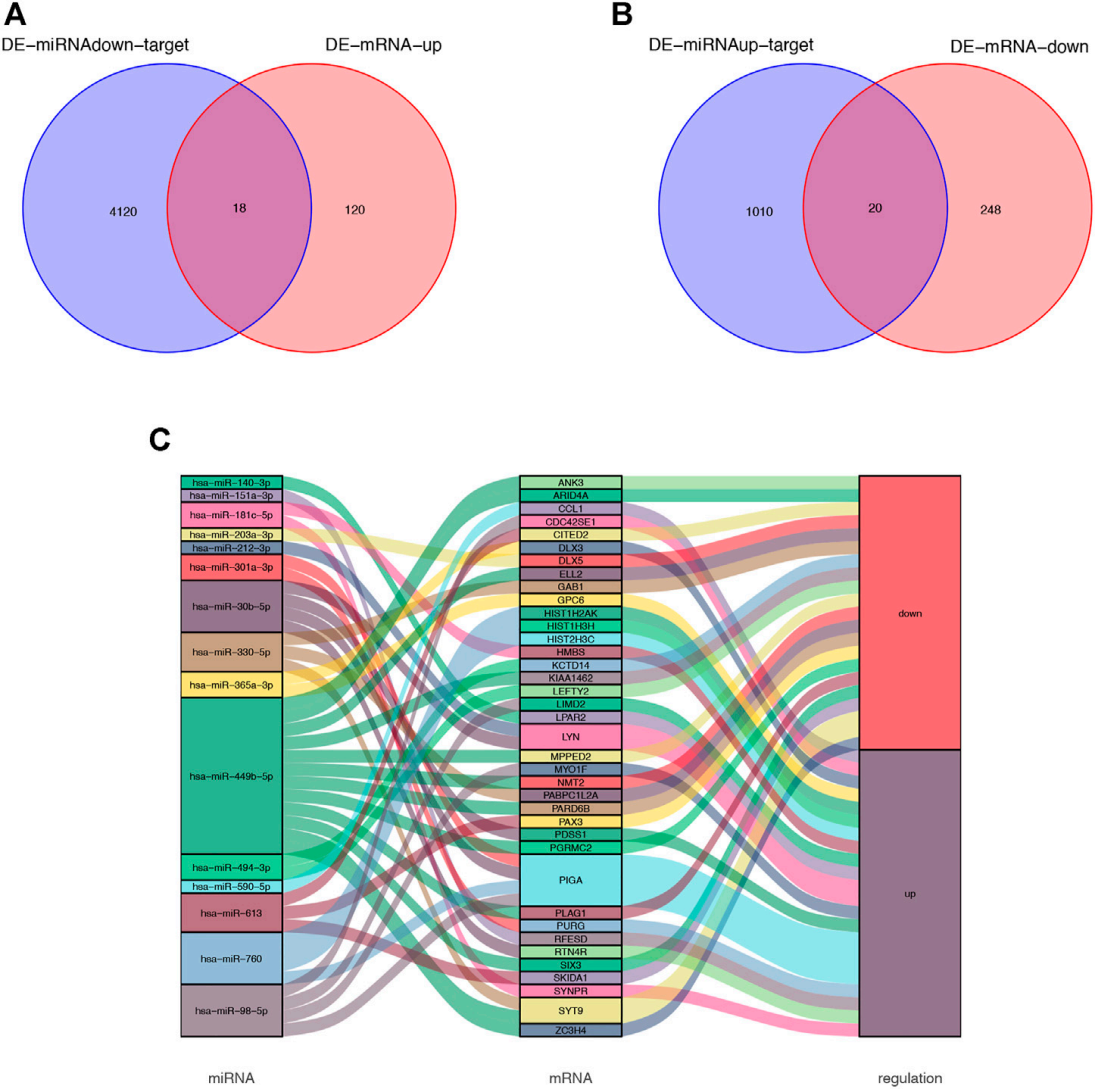
Venn diagram of DE-miRNA target genes and DE-mRNAs. **(A)** Venn diagram of 138 upregulated DE-mRNAs and 4,138 target genes of the downregulated DE-miRNAs. 18 overlapping mRNAs was filtered. **(B)** Venn diagram of 268 downregulated DE-mRNAs and 1,030 target genes of the upregulated DE-miRNAs. 20 overlapping mRNAs was filtered. **(C)** miRNA-mRNA network was visualized by Sankey diagram.

The bioinformatics software FunRich was used to predict 4,138 target genes of the downregulated DE miRNAs (Supplementary Table S4); the predicted target genes were then compared with 138 upregulated DE mRNAs; 18 overlapping upregulated DE mRNAs were visualized by using a Venn diagram (Figure 3A). Similarly, 1,030 target genes of the upregulated DE miRNAs were predicted by using FunRich and compared with 268 downregulated DE mRNAs; 20 overlapping downregulated DE mRNAs were visualized by using a Venn diagram (Figure 3B). The identified intersection genes are provided in Supplementary Table S5. Subsequently, dysregulated mRNAs were identified. Sankey diagram of the miRNA-mRNA network is illustrated in Figure 3C.

### Gene ontology enrichment analysis and kyoto encyclopedia of genes and genomes pathway enrichment analysis

**FIGURE 4:**
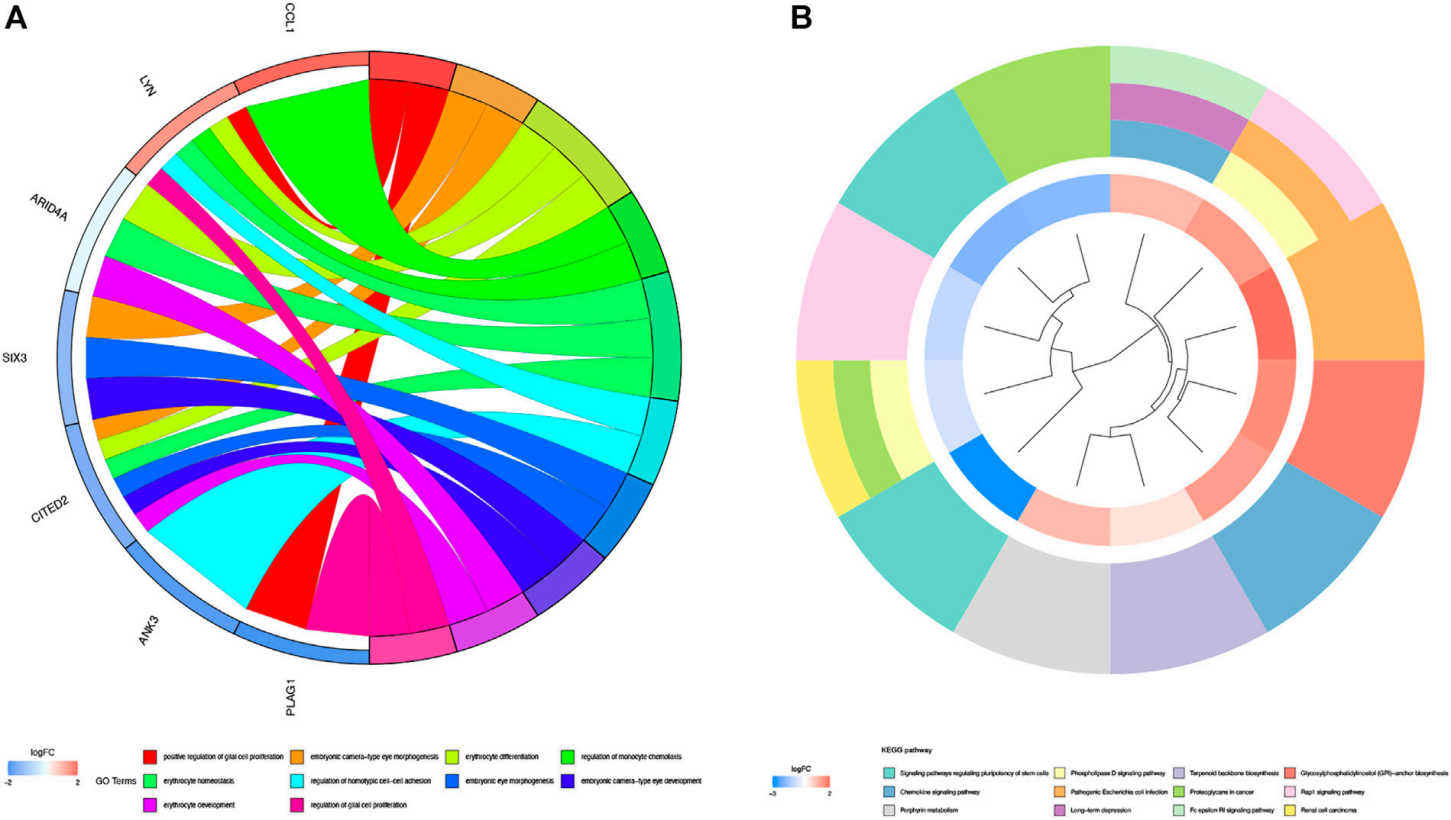
GO enrichment analysis and KEGG pathway analysis. **(A)** GO enrichment analysis of 38 genes in miRNA-mRNA network. Different color on left side of the graph represents different genes; GO entries are right side of the graph, magnitude of logFC values visualized by red and blue shades. **(B)** Graph shows entries of KEGG. Different KEGG pathway were visualized by different color modules. LogFC values of genes were visualized by innermost color.

GO enrichment analysis and KEGG pathway enrichment analysis were used to further explore the potential biological function of genes in the miRNA-mRNA network. The top five entries of Biological Process (BP) in the GO enrichment analysis were positive regulation of glial cell proliferation, embryonic camera-type eye morphogenesis, erythrocyte differentiation, regulation of monocyte chemotaxis, and erythrocyte homeostasis. The top five entries of Cellular Component (CC) were the integral component of the synaptic vesicle membrane, intrinsic component of the synaptic vesicle membrane, anchored component of the plasma membrane, cell-cell junction, and synaptic vesicle membrane. The top five entries of Molecular Function (MF) were glycosphingolipid binding, HMG-box domain binding, glycolipid binding, sphingolipid binding, and transferase alkyl or aryl activity (Figure 4A). The KEGG pathway enrichment analysis revealed that these genes mainly enrich the signaling pathways regulating the pluripotency of stem cells, phospholipase D signaling pathway, terpenoid backbone biosynthesis, glycosylphosphatidylinositol (GPI)-anchor biosynthesis, and chemokine signaling pathway (Figure 4B).

### Identification of hub genes

**FIGURE 5:**
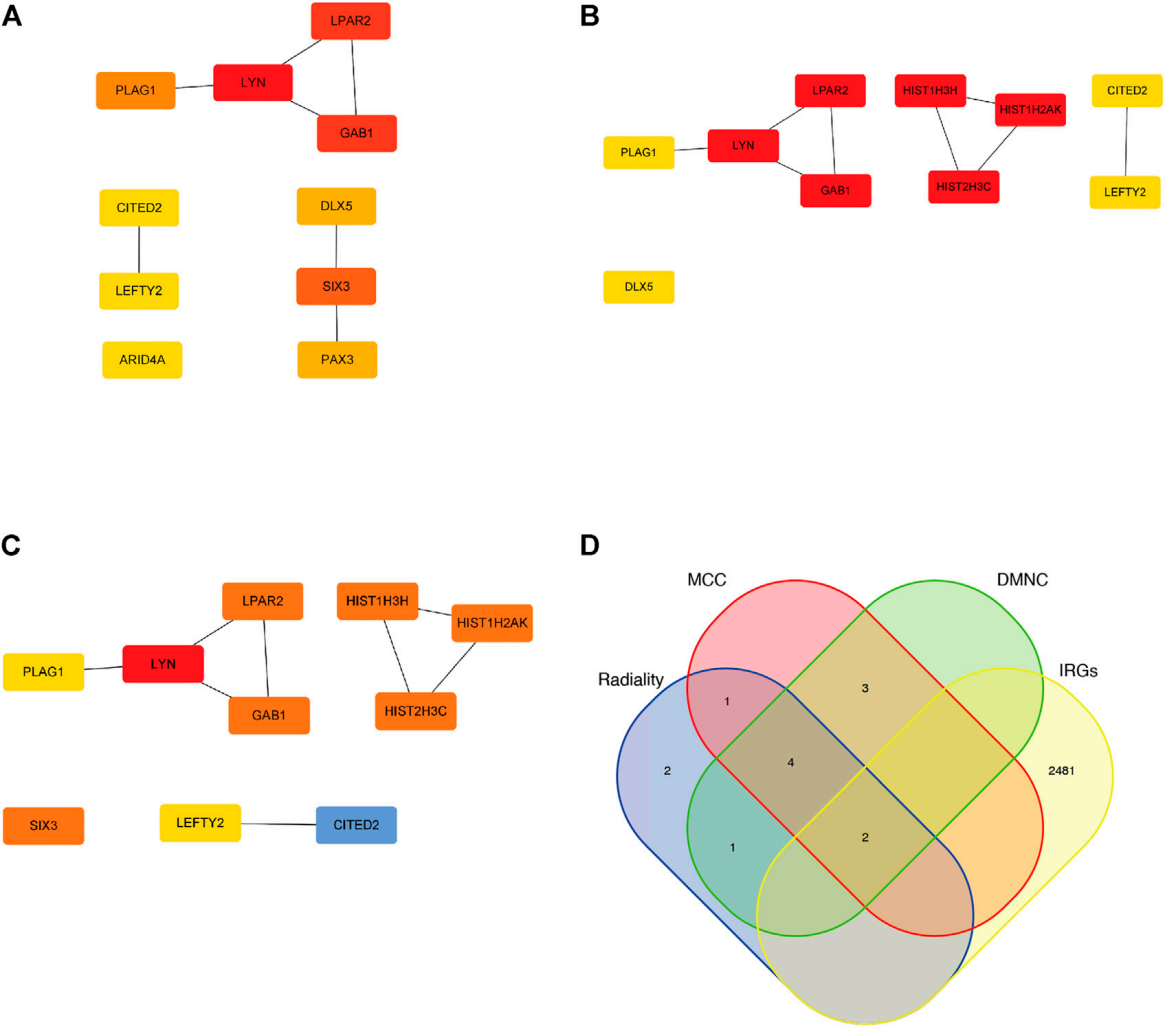
Hub genes of the miRNA-mRNA network. **(A)** Top 10 Hub genes by utilizing Radiality algorithm. **(B)** Top 10 Hub genes by utilizing DMNC algorithm. **(C)** Top 10 Hub genes by utilizing MCC algorithm. **(D)** two overlapping genes were filtered by utilizing venn diagram.

Thirty-eight intersection genes were imported into the STRING online database. We filtered ten hub genes by using three cytoHubba algorithms (Radiality, DMNC, and MCC) (Figures 5A–C); 2,483 immune-related genes (IRGs) were compared with the identified hub genes and presented in a Venn diagram (Figure 5D). Two overlapping genes were filtered, *LYN* (LYN Proto-Oncogene, Src Family Tyrosine Kinase) and *LEFTY2* (Left-Right Determination Factor 2).

### Immune infiltration-correlated cells and genes of the microarray

**FIGURE 6:**
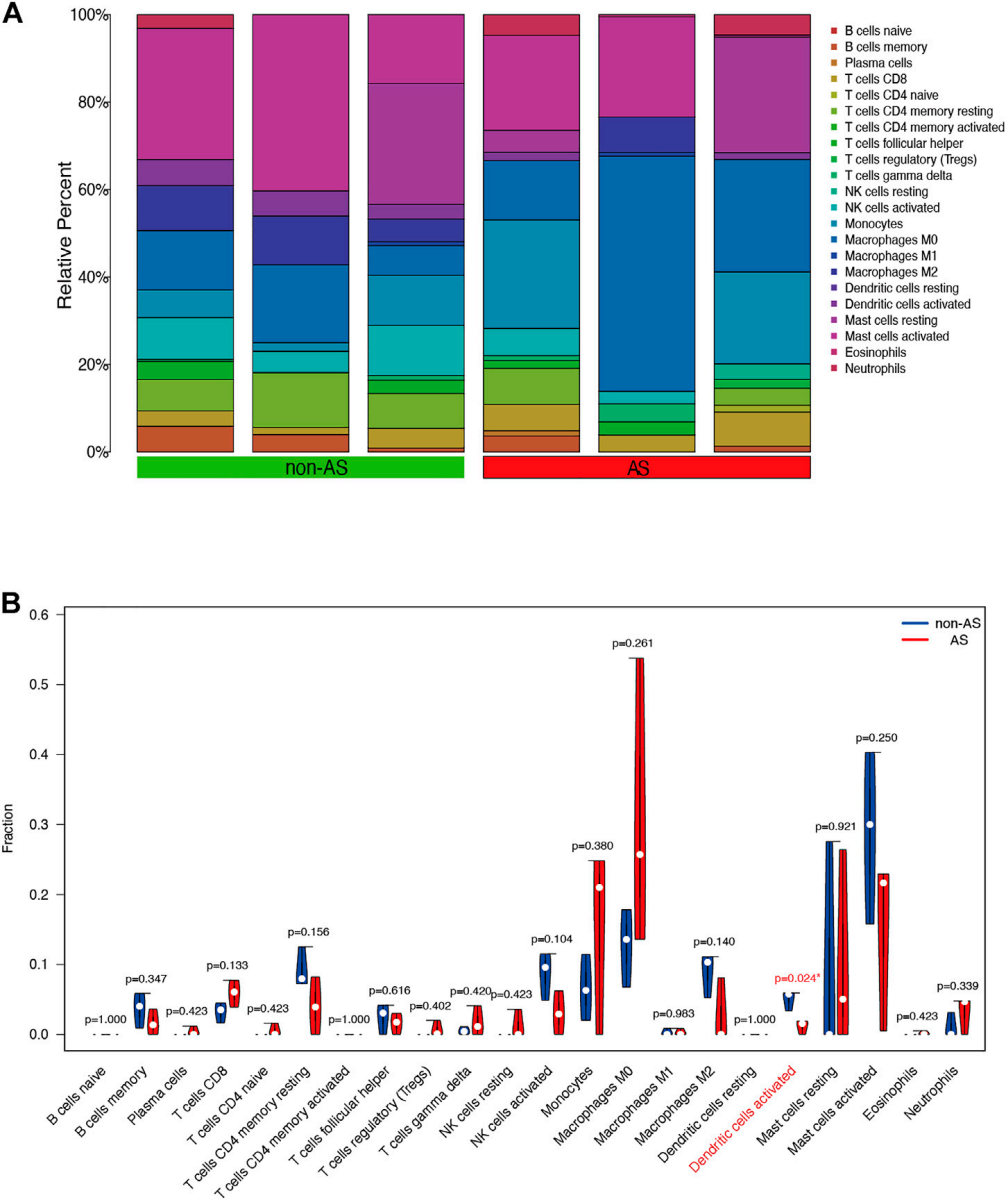
Immune cells composition diagram and immune cells violin plot of AS microarray. **(A)** Immune cells composition of each sample was visualized by histogram. **(B)** indicates the differences in the composition of the 22 immune cells based on AS microarray.

**FIGURE 7:**
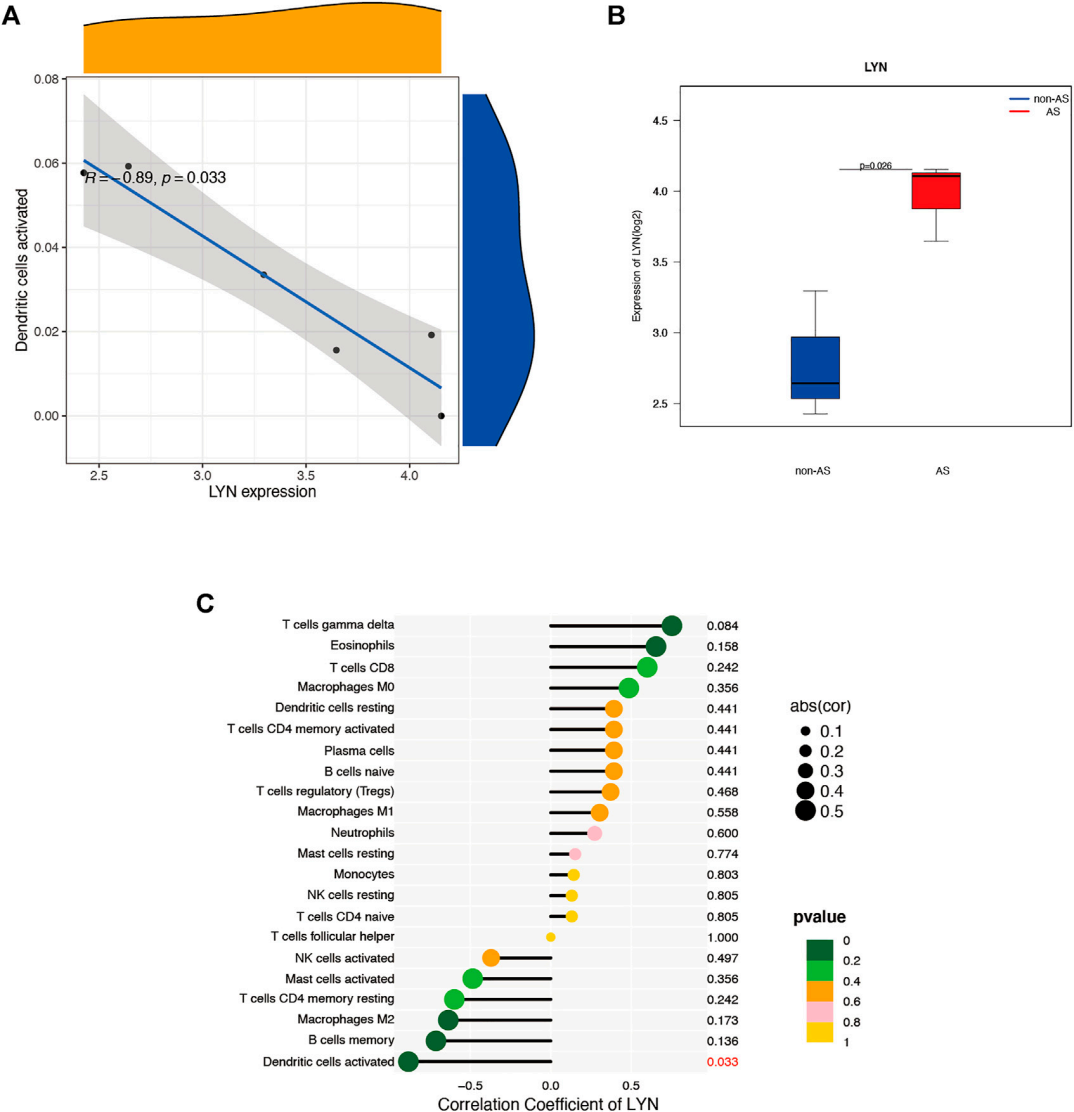
Immune cells correlation graph of *LYN* base on AS microarray. **(A)** Correlation plot of *LYN* with dendric cells activated. **(B)** *LYN* expression in the AS microarray. **(C)** Correlation coefficient of *LYN* with 22 immune cells.

We used the CIBERSORT software to analyze the immune cell infiltration of the obtained microarray. Constitute plots revealed the presence of 22 immune cell types in the interspinous ligament tissue samples of each patient (Figure 6A). The violin plot of immune cell components revealed significant dendritic cell activation in the samples of three patients with AS and three patients without AS (Figure 6B). Figure 7A depicts the significant correlation of *LYN* with dendritic cell activation (*p* < 0.05). Moreover, the expression of *LYN* was significantly different in patients with AS and patients without AS (Figure 7B). Figure 7C provides the correlation coefficient of *LYN* and immune cells.

### Clinical data analysis and diagnostic model of Ankylosing Spondylitis

**FIGURE 8:**
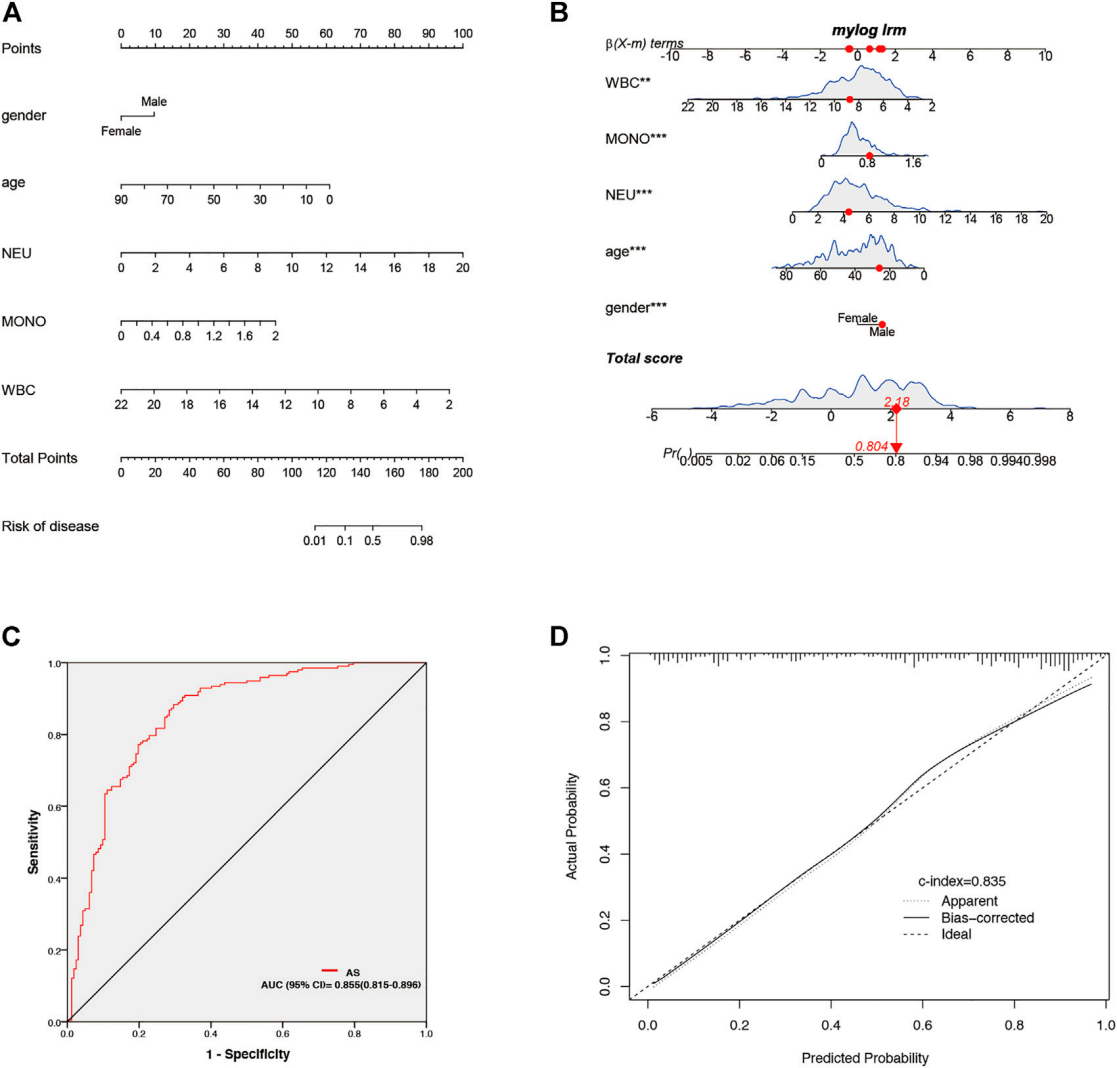
Establishment and validation of nomogram for predicting of AS patients. **(A)** Nomogram for predicting of AS patient. **(B)** A nomogram represents an AS patient. **(C)** AUC of the nomogram. **(D)** Calibration curves for predicting AS patients for clinical data.

Clinical data of 359 patients are tabulated in Table 1. Patient characteristics, such as age and sex, were included in this study. We also obtained immune cell data (white blood cells, monocytes, neutrophils, lymphocytes, basophils, and eosinophils) from routine blood tests. Mann-Whitney U test revealed that age, gender, and counts of white blood cells, neutrophils, monocytes, and eosinophils were significantly different in patients with AS and patients without AS (*p* < 0.05) (Table 1). Univariate logistic regression revealed that sex and counts of white blood cells, monocytes, and neutrophils correlated with AS. Furthermore, multivariate logistic regression was performed, and five independent risk factors related to the diagnosis of AS, including gender, age, and counts of white blood cells, monocytes, and neutrophils, were screened out (Table 2). The five independent risk factors were used to construct a nomogram (Figure 8A). Figure 8B depicts a nomogram sample of a patient with AS. Area under curve (AUC) of the nomogram ROC was 0.855 (95% CI 0.815–0.896) (Figure 8C). The actual and prediction probabilities of the nomogram were validated by calibration curves (Figure 8D), and the C-index of the calibration curves was 0.835. Positive predictive values (PPV) and negative predictive values (NPV) are depicted in Supplementary Figure S2.

**TABLE 1:** Baseline characteristics between AS patients and non-AS patients.

Clinical data	AS	Non-AS	*p*-value
	(*n* = 197)	(*n* = 162)	
Age			<0.01
Mean (SD)	30.73 (6.59)	47.99 (17.15)	
median [P25, P75]	30 [24, 37]	51 [36, 60]	
Gender			<0.01
Male	172 (87.31%)	92 (56.79%)	
Female	25 (12.69%)	70 (43.21%)	
WBC			<0.01
Mean (SD)	8.49 (2.08)	7.62 (2.56)	
median [P25, P75]	8.2 [7.14, 9.81]	7.08 [5.84, 8.95]	
NEU			<0.01
Mean (SD)	5.47 (1.83)	4.72 (2.48)	
median [P25, P75]	5.31 [4.18, 6.53]	4.07 [3.13, 5.50]	
LYM			0.216
Mean (SD)	2.16 (0.77)	2.06 (0.78)	
median [P25, P75]	2.09 [1.72, 2.44]	1.95 [1.47, 2.56]	
MONO			<0.01
Mean (SD)	0.68 (0.24)	0.58 (0.20)	
median [P25, P75]	0.62 [0.51, 0.83]	0.55 [0.44, 0.69]	
BAS			0.207
Mean (SD)	0.04 (0.03)	0.04 (0.02)	
median [P25, P75]	0.04 [0.03, 0.05]	0.032 [0.025, 0.045]	
EOS			<0.01
Mean (SD)	0.19 (0.17)	0.23 (0.17)	
median [P25, P75]	0.14 [0.8, 0.26]	0.20 [0.12, 0.29]	

WBC, white blood cells; NEU, neutrophils; LYM, lymphocytes; MONO, monocytes; BAS, basophils; EOS, eosinophils.

**TABLE 2:** Univariate and multivariate logistic regression used for identifying independent diagnostic factors to distinguish AS patients from healthy controls.

Clinical data	Univariate logistic regression		Multivariate logistic regression	
	OR [95% CI]	*p*-value	OR [95% CI]	*p*-value
Gender (male)	3.87 [2.06, 7.27]	<0.001***	0.27 [0.14, 0.51]	<0.001***
Age	0.91 [0.89, 0.93]	<0.001***	0.91 [0.89, 0.93]	<0.001***
MONO	22.09 [4.04, 120.90]	<0.001***	21.32 [3.97, 114.55]	<0.001***
NEU	1.67 [1.10, 2.51]	0.016*	1.97 [1.35, 2.87]	<0.001***
WBC	0.60 [0.40, 0.92]	0.018*	0.52 [0.35, 0.77]	0.001***
EOS	0.19 [0.03, 1.04]	0.056	0.19 [0.03, 1.02]	0.06

WBC, white blood cells; NEU, neutrophils; MONO, monocytes; EOS, eosinophils.

### Data download and identification of differentially expressed genes

**FIGURE 9:**
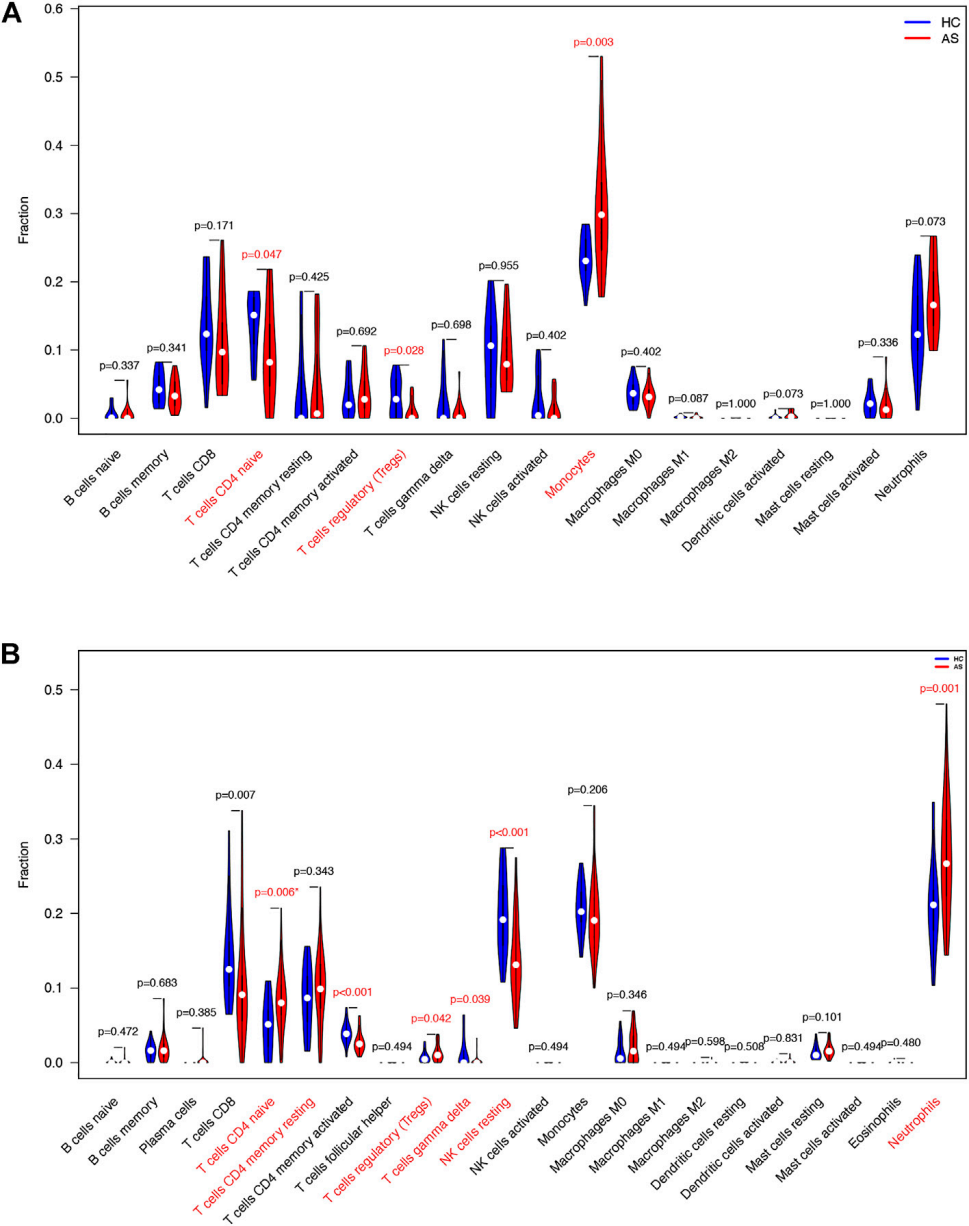
Immune cells violin plot of GEO datasets. **(A)** Immune cells violin plot of GSE 25101. Red text illustrates significant different immune cells and *p*-value< 0.05. **(B)** Immune cells violin plot of GSE 73754. Red text illustrates significant different immune cells and *p*-value< 0.05.

**FIGURE 10:**
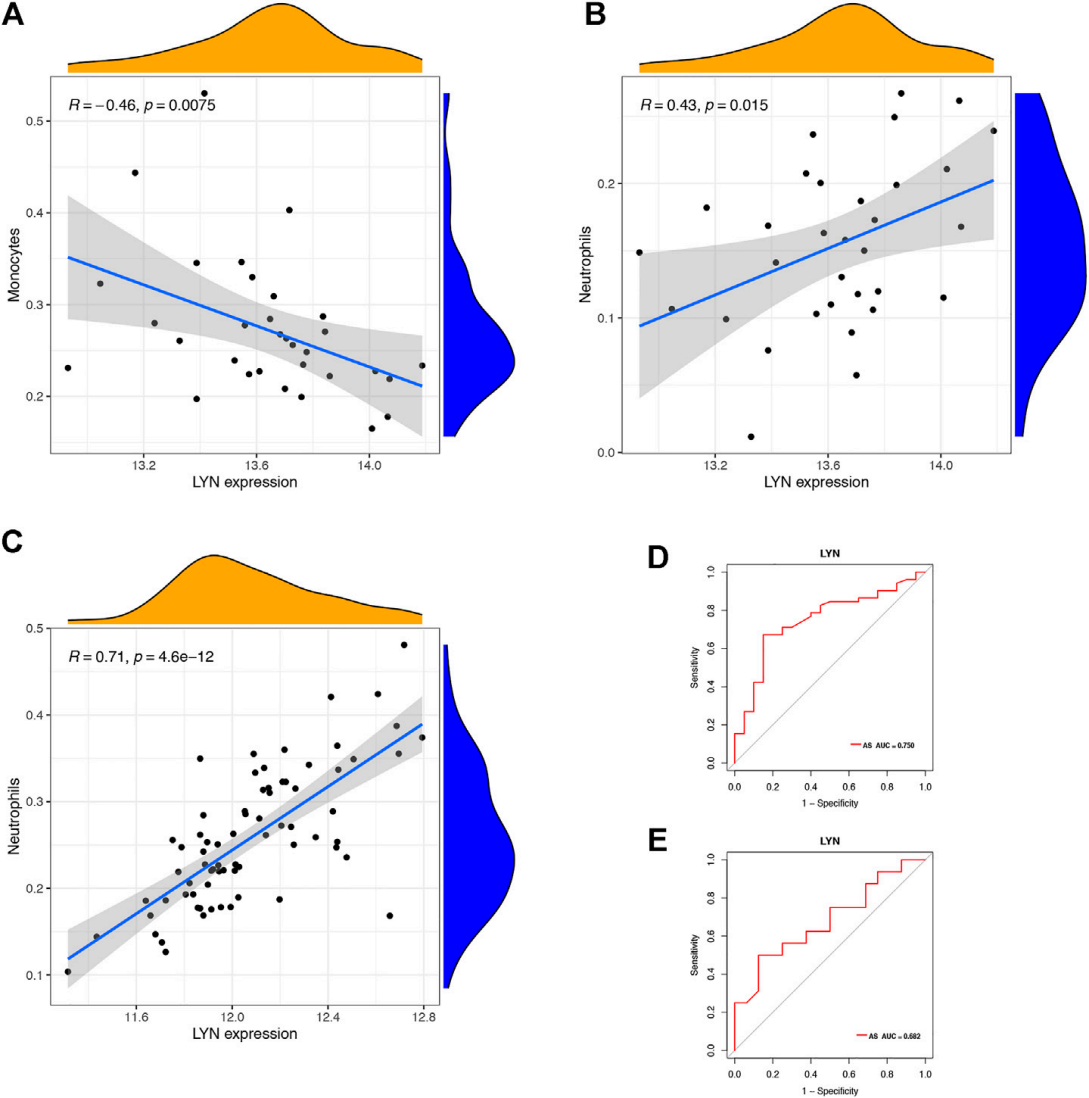
Immune cells correlation graph of *LYN* base on GEO datasets. **(A)** Correlation plot of *LYN* with monocytes base on GSE 25101. **(B)** Correlation plot of *LYN* with neutrophils base on GSE 25101. **(C)** Correlation plot of *LYN* with monocytes base on GSE 73754. **(D)** ROC curve of *LYN* as a diagnostic gene base on GSE 25101. **(E)** ROC curve of *LYN* as a diagnostic gene base on GSE 73754.

We downloaded GSE25101 and GSE73754 datasets, consisting of peripheral blood gene expression data, from the GEO database. CIBERSORT software outcomes of GSE25101 and GSE73754 validated the immune cell components of the clinical data. The number of monocytes (Figure 9A) and neutrophils (Figure 9B) were significantly different in patients with AS and patients without AS. Moreover, *LYN* was DEG in GSE25101 and GSE73754 (Supplementary Figure S1; Supplementary Table S6). We further explored the correlation between *LYN* and immune cells. Figures 10A–C reveal the significant correlation of *LYN* with monocytes and neutrophils in GSE25101 and GSE73754 (*p* < 0.05) datasets. Figure 10D,E illustrate the performance of *LYN* as a diagnostic gene in GSE 25101 and GSE 73754 datasets; the AUC for GSE 25101 and GSE 73754 were 0.750 and 0.682, respectively. The expression of *LYN* are provided in Supplementary Table S6.

### Immunohistochemical analysis results

**FIGURE 11:**
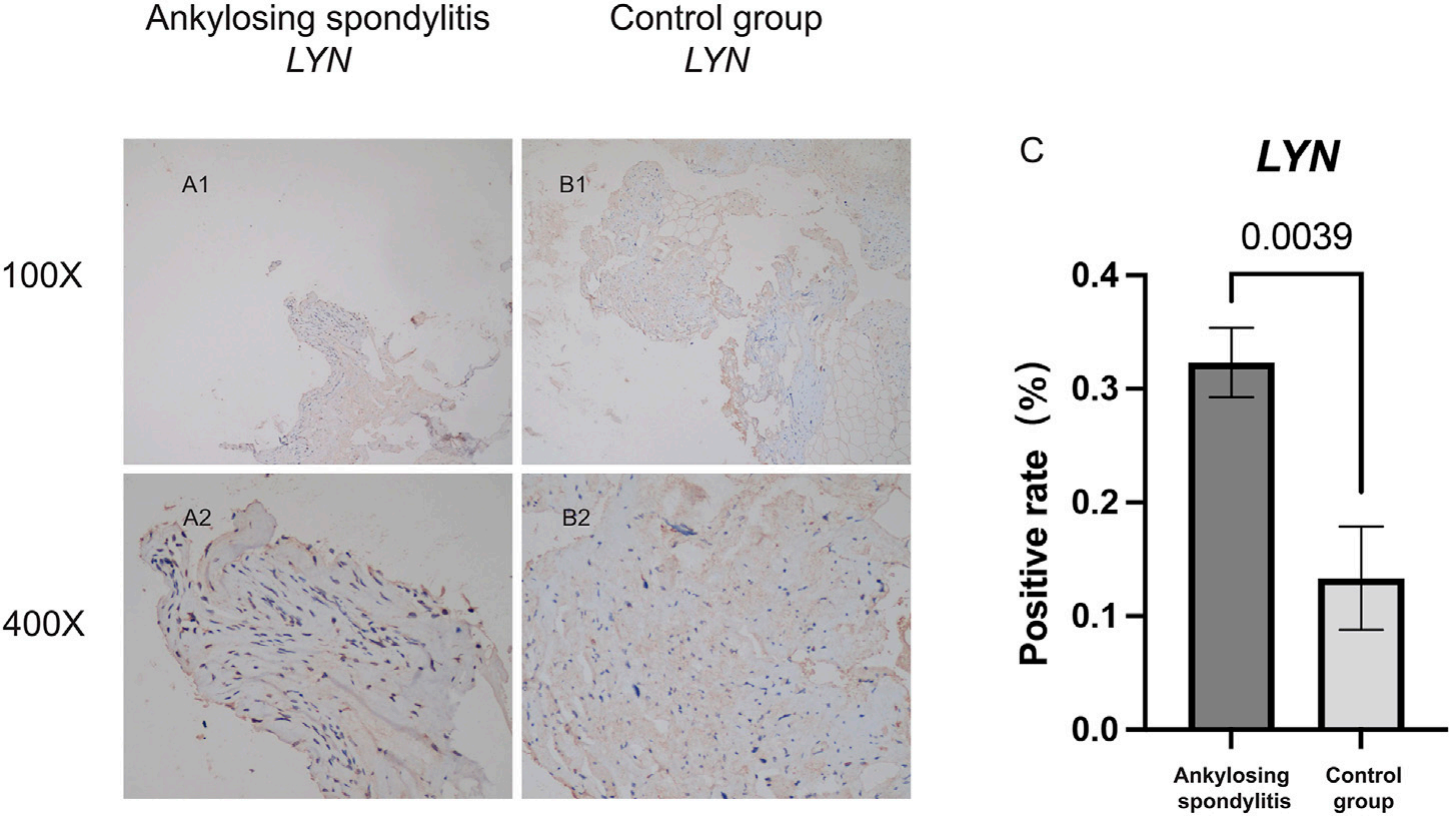
Immunohistochemical staining analysis. **(A,B)** Shows the specific expression of *LYN* in AS group and the non-AS group. **(C)** Shows the statistical analysis results of the positivity rate between AS group and the non-AS group.

Immunohistochemical staining was performed for *LYN* in five patients with ankylosing spondylitis and three patients with spinal fracture. Clinical characteristics of five patients with ankylosing spondylitis and three patients with spinal fracture are included in Supplementary Table S7. Figures 11A,B shows that the specific expression of *LYN* in AS group was significantly higher than that in the control group. We detected the positive rate of Immunohistochemical images by using ImageJ software. Positive rate data of *LYN* were imported into SPSS 22.0, and the difference between the two groups was statistically analyzed by independent samples t-test. The positive rate of *LYN* gene in AS group was significantly higher than that in the normal control group (Figure 11C) (*p* < 0.05). It demonstrates that *LYN* is differentially expressed in AS group and control group. This result confirms the accuracy of our analysis.

## Discussion

Although AS treatment has improved considerably because of developments in medical science, its diagnosis remains a challenge; this is mainly due to the lack of tissue biomarkers. mi-RNA regulates gene expression and has a key role in the development and occurrence of AS (Qin et al., 2019). However, the regulatory network of miRNA-mRNA in AS remains unclear. Blood *HLA-B27* testing is widely used in AS diagnosis (Li et al., 2021); however, in many countries, it is not a routine health examination, and its suitability for the public screening is doubtful (Brown et al., 1996). In addition, *HLA-B27* is not the only major histocompatibility complex (MHC) gene associated with AS susceptibility (Brown et al., 2002). *HLA-B27* testing is not a routine health examination and is unsuitable for the general investigation of chronic back pain patients. Thus, efficient biomarkers and clinical diagnostic model associated with AS must be explored to improve its diagnosis.

On the basis of the AS microarray, we constructed a miRNA-mRNA network consisting of 38 DEGs. GO enrichment analysis was performed for 38 DEGs, and the regulation of monocyte chemotaxis was one of the top five entries. A previous study demonstrated that monocytes contributed to proinflammatory responses in AS (Shi et al., 2020). The chemokine signaling pathway was one of the top five entries in the KEGG enrichment analysis. A recent study indicated that CXCL5, a chemokine CXC subfamily, played an important role in the osteoclastogenesis of AS (Liu et al., 2019). Moreover, we identified that dendritic cell activation was significantly different in patients with AS and patients without AS. Subsequently, six hub genes were filtered from the 38 DEGs: *LYN*, *CITED2*, *GAB1*, *LEFTY2*, *LPAR2*, and *PLAG1*. We further demonstrated that *LYN* was correlated with dendritic cell activation in interspinous ligament tissue samples of patients with AS. Previous studies have indicated that dendritic cell-induced autoimmunity and inflammation resulted from LYN dysregulation (Lamagna et al., 2013; Ma et al., 2019). The statistical analysis of the clinical data revealed four diagnostic factors for AS, including age, sex, neutrophils, and monocytes. Monocytes, neutrophils, and dendritic cells originate from colony-forming unit granulocyte and macrophage (CFU-GM) (Tang-Huau and Segura, 2019). Besides, parts of dendritic cells stem from monocytes (Hamilton, 2020). To further validate the significant difference in peripheral blood monocytes and neutrophils, we downloaded two gene datasets from the GEO database, GSE25101 and GSE73754. Immune infiltration of peripheral blood base in the GSE25101 dataset revealed a significant difference in the components of monocytes. Similarly, immune infiltration of peripheral blood base in the GSE73754 dataset revealed a significant difference in the components of neutrophils. On the basis of these results, we constructed a clinical diagnostic model for AS and validated the prediction probabilities.

*LYN*, known as Src Family Tyrosine Kinase, is expressed by B cells, myeloid cells, and dendritic cells and has special regulatory properties. It activates activating signals and inactivates inhibitory signals, thereby moderating cell activation and leading to tolerance (Scapini et al., 2009). In an *in vitro* study, the activation of TLR-triggered signaling in dendritic cells was observed after *LYN* deletion, leading to the spontaneous development of an autoimmune disease with features of human systemic lupus erythematosus (Lamagna et al., 2013). We validated the correlation of *LYN* and dendritic cell activation in the immune infiltration analysis of interspinous ligament tissue samples. A recent study indicated that immune cells and immune mediators play a key role in the pathogenesis of AS, which is an immune-related disease (Sveaas et al., 2015). Animal experiments have demonstrated that spondyloarthritis-prone *HLA-B27* transgenic rat-origin dendritic cells exhibited several abnormalities, including cytoskeletal alterations, the downregulation of MHC class II molecules, the impaired stimulation of T-cell response, increased apoptotic death, regulatory T-cell function regulation, and the preferential induction of type 17 helper T-cell expansion (Fert et al., 2008; Araujo et al., 2014). In this study, we chose interspinous ligament tissue samples from six patients (three with AS and three without). miRNA and mRNA microarrays were built using the extracted RNA. Using bioinformatics methods, we identified *LYN* as an immune-related hub gene associated with immune cell infiltration in AS patients. It was discovered that *LYN* expression was significantly upregulated in the interspinous ligament of patients with AS using bioinformatics analysis of microarray data (Figure 7B). Because the number of microarray samples was limited (three patients with AS and three patients without AS), immunohistochemistry analysis of interspinous ligament samples (five patients with AS and three patients without AS) from eight additional patients undergoing surgery was performed to validate the bioinformatics analysis results. Figure 11 depicts the immunohistochemistry results. The AS group had a significantly higher positive rate of *LYN* than the control group (Figure 11C) (*p* < 0.05). To sum up, the findings of the above-mentioned studies validated that *LYN* is related to dendritic cell activation and plays a key role in AS pathogenesis.

AS is an inflammatory autoimmune disease (Liang et al., 2021), and monocytes or mononuclear phagocytes play an important role in inflammatory regulation (Serbina et al., 2008). During development, monocytes are involved in homeostasis and inflammation release (Ginhoux and Jung, 2014). A previous study reported that autoimmune disorders, such as rheumatoid arthritis (RA), multiple sclerosis (MS), and systemic lupus erythematosus (SLE), are closely related to monocytes (Narasimhan et al., 2019). Neutrophils constitute the largest number of white blood cells in the human blood and play a key role in chronic inflammatory diseases, such as atherosclerosis, diabetes mellitus, nonalcoholic fatty liver disease, and autoimmune disorders (Herrero-Cervera et al., 2022). Autoimmune disorders, such as RA, MS, SLE (O'Neil and Kaplan, 2019; Woodberry et al., 2018; De Bondt et al., 2020; Tsai et al., 2019), and inflammatory bowel disease (IBD) (Herrero-Cervera et al., 2022) are also implicated to neutrophils. The results of our study demonstrated that the counts of monocytes and neutrophils were significantly higher in patients with AS compared with patients without AS. Therefore, monocyte and neutrophil counts are the independent diagnostic factors for AS. Furthermore, we used the independent diagnostic factors of age, sex, monocyte count, and neutrophil count to construct a nomogram for AS. The efficiency of the constructed nomogram was validated, and it could detect AS among patients with chronic back pain.

By performing bioinformatic analysis on the datasets obtained from the GEO database, we concluded that monocytes and neutrophils were related to the immune infiltration in peripheral blood of patients with AS. On the basis of GEO datasets analysis (GSE25101 and GSE73754), we concluded that *LYN* is a DEG. Besides, the results of immune infiltration indicated that *LYN* was correlated with monocytes and neutrophils. This result is consistent with that of a previous study, which indicated that the dysregulation of *LYN* affected the migration and adhesion of monocytes and neutrophils (Brian Iv and Freedman, 2021; Das et al., 2021).

**FIGURE 12:**
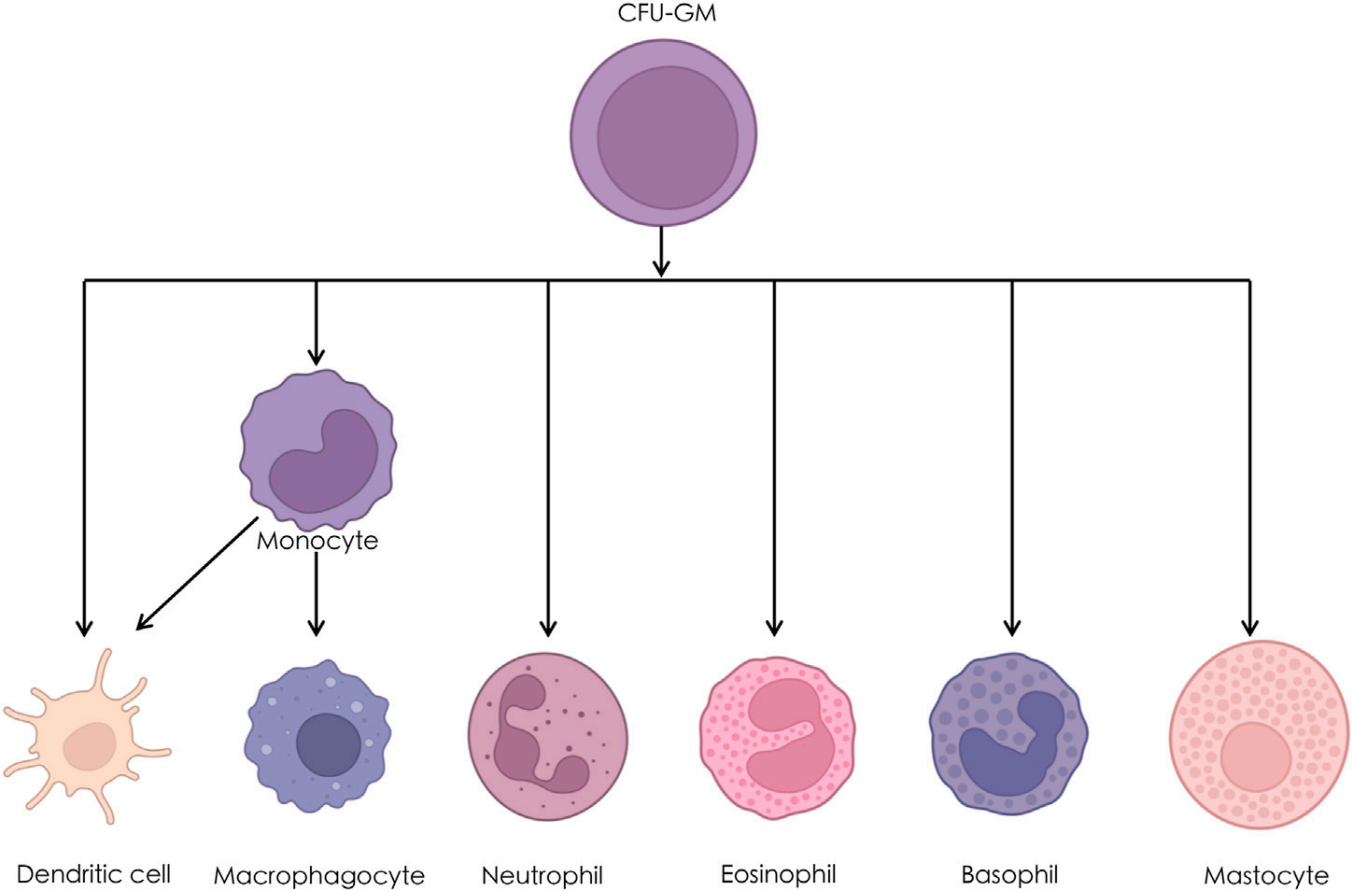
The differentiation of Colony Forming Unit-Granulocyte and Macrophage (CFU-GM).

*LYN*, a DEG in AS, was significantly correlated with the counts of monocytes and neutrophils and dendritic cell activation. The statistical analysis of the clinical data demonstrated that neutrophils and monocytes were the diagnostic factors for AS. Furthermore, we validated the correlation of LYN with monocytes and neutrophils by performing a bioinformatic analysis of immune cell infiltration. On the basis of microarray analysis, we concluded that *LYN* was related to dendritic cell activation and AS pathogenesis; the dendritic cell components were different in patients with AS and patients without AS. To sum up, *LYN* was related to dendritic cell activation in patients with AS patients; it was also correlated with monocytes and neutrophils infiltration in the peripheral blood of patients with AS. The three *LYN*-correlated cells, dendritic cells, monocytes, and neutrophils, originate from different tissues. Immune cells play an important role in the human body, and abnormal immune cell function often causes pathological changes related to AS (McVeigh and Cairns, 2006; Rezaiemanesh et al., 2018). Besides, dendritic cells, monocytes, and neutrophils stem from CFU-GM (Tang-Huau and Segura, 2019). Figure 12 illustrates the differentiation of CFU-GM. A part of dendritic cells stem from monocytes and are called monocyte-derived dendritic cells (Qu et al., 2014). Some of these dendritic cells are related to nonself-antigens immunity, and others contribute to self-antigens tolerance (Rezaiemanesh et al., 2018). We hypothesized that *LYN* might participate in the biological processes of cells during AS pathogenesis. Similar to the findings of a previous study, the results of GO enrichment analysis in this study indicated that *LYN* participated in the regulation of monocyte chemotaxis (Das et al., 2021). Another study indicated that *LYN* regulates the differentiation of monocytes to dendritic cells by blocking the Toll-like receptor signaling pathway (Galgani et al., 2004). Another study on the role of Src family kinases in inflammatory response demonstrated that *LYN* is one of the key regulators in the activation, proliferation, and migration of neutrophils (Kovács et al., 2014). Therefore, *LYN* plays a key role in the regulation of monocyte chemotaxis, differentiation of monocytes to dendritic cells, and neutrophil inflammatory response.

After exploring the correlation between *LYN* and immune cell infiltration, we focused on the clinical diagnosis of AS. It is difficult but important to effectively diagnose AS in patients with chronic low back pain (McVeigh and Cairns, 2006). Although *HLA-B27* testing has been widely utilized in clinical testing for AS, it has been reported that at least 8% of Europeans carry *HLA-B27*, of which 5% will develop AS (Brown et al., 1996). Not all *HLA-B27* positive patients can be diagnosed with AS, and *HLA-B27* negative AS patients are also reported in clinical practice (Brown et al., 1996). In addition, non-MHC components have a diagnostic value for AS. The study by Li et al. (2021) revealed that Polygenic Risk Scores (PRS) testing had a greater discriminatory capacity for AS than *HLA-B27* testing, and the AUC of PRS and *HLA-B27* testing was 0.924 (95% CI 0.920–0.928) and 0.869 (95% CI 0.865–0.874), respectively. Consequently, AS can be identified effectively without *HLA-B27* testing. Age, gender, and counts of monocytes, neutrophils, and white blood cells were identified as six independent diagnostic factors of AS in our study, which was based on clinical characteristics and routine blood tests. AS mainly affects young adults (Ritchlin and Adamopoulos, 2021), and its prevalence is higher in male patients than in female patients (Taurog et al., 2016). This is consistent with the conclusion that gender and age are independent risk factors in this diagnostic model. The pathogenesis of AS is a complex process involving immune cells (Rezaiemanesh et al., 2018). In this study, biomarkers and immune cells related to AS were explored and verified using the GEO database. The counts of monocytes, neutrophils, and white blood cells were also the independent risk factors for AS. We visualized this diagnostic model by nomogram, whose accuracy was verified by the ROC and calibration curves (Figures 8C,D). The ROC analysis (AUC) of *HLA-B27* testing, MRI, and C-reactive protein (CRP) in AS were reported to be 0.869, 0.620, and 0.700, respectively (Rudwaleit et al., 2004; Ye et al., 2020; Li et al., 2021). In this study, the diagnostic model for AS with an AUC of 0.855 was superior to MRI (AUC = 0.620) and C-reactive protein (AUC = 0.700) as predictors of AS. In clinical practice, we can use this diagnostic model to score patients with chronic low back pain and screen out patients with AS, leading to an increased accuracy of initial AS screening.

There are several limitations to our study. First, because this is a retrospective study, the non-AS group included patients who had symptoms of thoracic spinal cord compression or lumbar spinal stenosis. There is no data on *HLA-B27* testing in the non-AS group. Statistical analyses involving *HLA-B27* were difficult to carry out. Furthermore, a selection bias cannot be ruled out in this retrospective study. Second, while this study built a clinical diagnostic model of AS, the participants were from a single center; multi-center external verification with larger sample size is still required. Third, despite the fact that the results of the bioinformatics analysis of the AS microarray in this study were confirmed by immunohistochemical analysis, the sample size was small. Finally, this study discovered that has-miR-30b-5p and has-miR-212-3p are linked to *LYN*, but the mechanism is unknown. Additional laboratory analyses, as well as external, independent data validation, are required to validate our findings.

## Conclusion

*LYN* correlated with immune cell infiltration in patients with AS. In addition, the counts of monocytes and neutrophils were the independent diagnostic factors for AS. Thus, these findings may be used to develop a diagnostic model to predict AS, and its efficiency must be verified in future studies.

## Data Availability

The datasets presented in this study can be found in online repositories. The names of the repository/repositories and accession number(s) can be found in the article/Supplementary Material.
