# Supplementary figures and images for: Immune cell infiltration-related clinical diagnostic model for Ankylosing Spondylitis
Source: Front Genet. 2022 Sep 5;13:949882. doi: 10.3389/fgene.2022.949882 (PMC9575679; doi:10.3389/fgene.2022.949882)

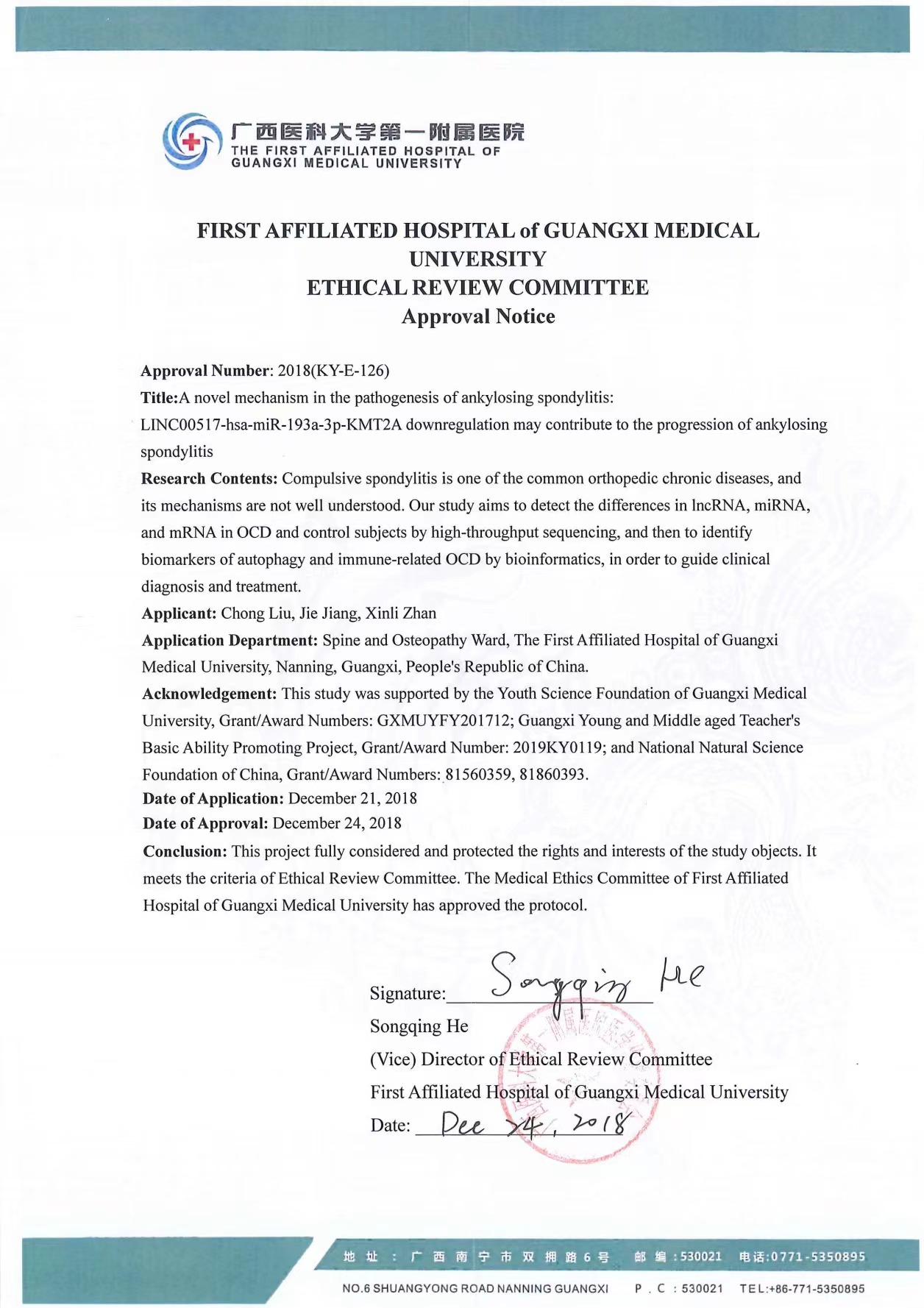

Supplement: Supplementary file 2 [file Image3.JPEG]

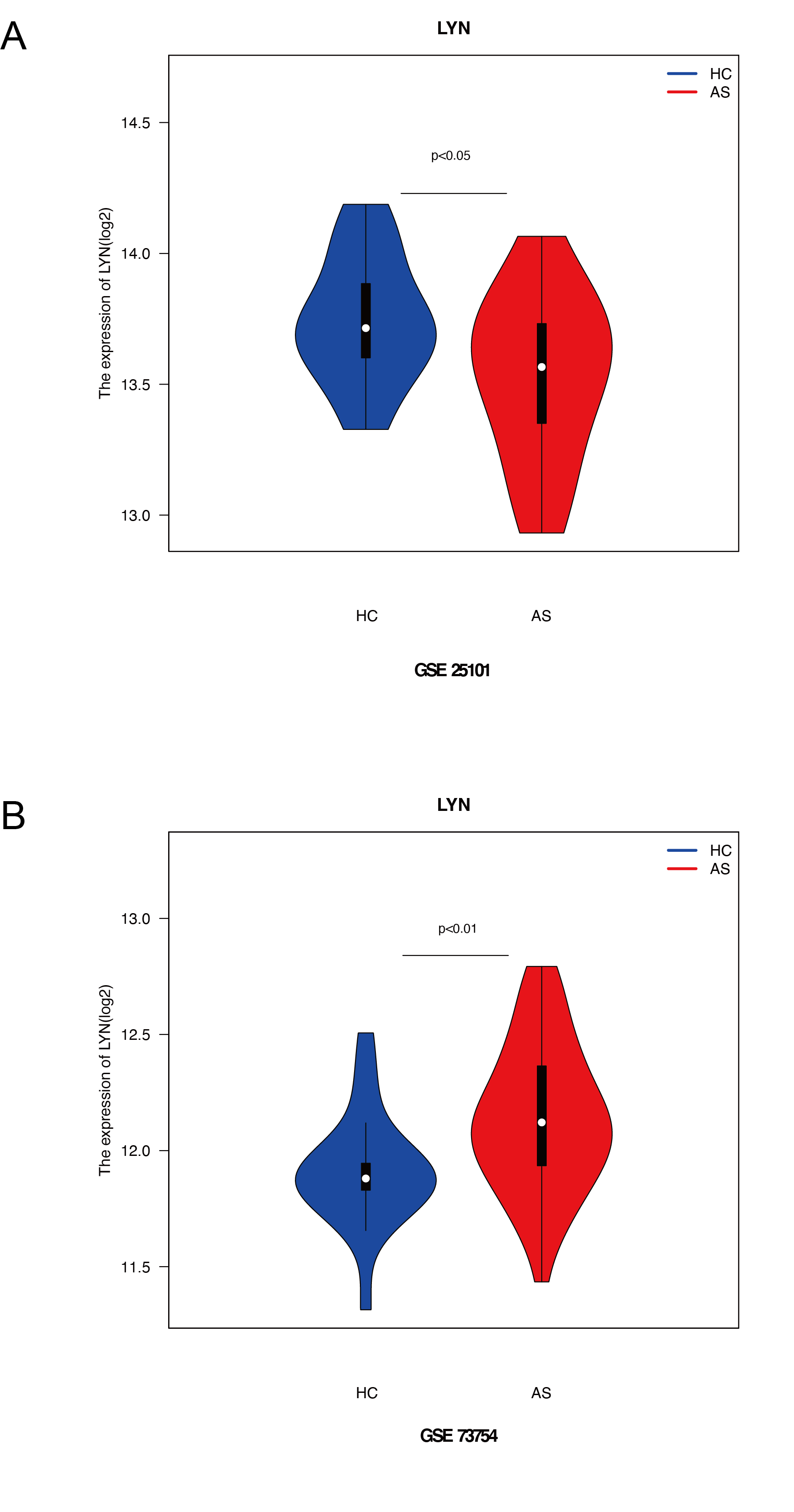

Supplement: Supplementary file 6 [file Image1.JPEG]

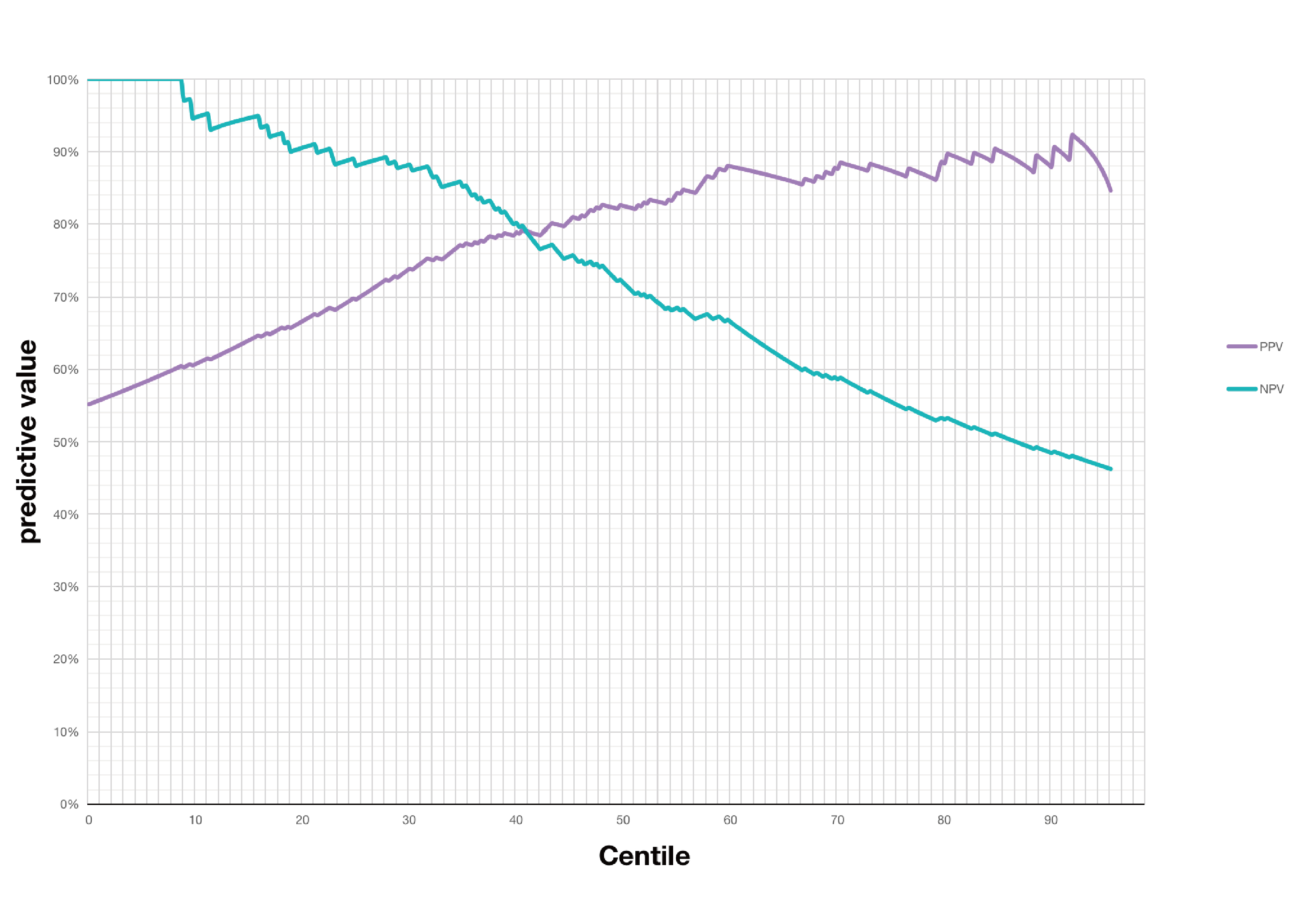

Supplement: Supplementary file 8 [file Image2.JPEG]
