# Supplementary material for: Immune cell infiltration-related clinical diagnostic model for Ankylosing Spondylitis
Source: Front Genet. 2022 Sep 5;13:949882. doi: 10.3389/fgene.2022.949882 (PMC9575679; doi:10.3389/fgene.2022.949882)
Supplement: Supplementary file 3 [file Table1.DOCX]

**Supplement Table 1**

Patients’ clinical characteristics in microarray

| Clinical characteristics | AS patients | | | non-AS patients | | |
| --- | --- | --- | --- | --- | --- | --- |
|  | AS1 | AS2 | AS3 | nonAS1 | nonAS2 | nonAS3 |
| Age | 55 | 24 | 44 | 56 | 52 | 26 |
| Gender | Male | Male | Male | Male | Male | Male |
| HLA-B27 | + | + | + | - | - | - |
| Bilateral sacroiliac joint injuries | + | + | + | - | - | - |
| Spinal and sacroiliac joint fusion | + | + | + | - | - | - |
