# Supplementary material for: Immune cell infiltration-related clinical diagnostic model for Ankylosing Spondylitis
Source: Front Genet. 2022 Sep 5;13:949882. doi: 10.3389/fgene.2022.949882 (PMC9575679; doi:10.3389/fgene.2022.949882)
Supplement: Supplementary file 4 [file Table8.docx]

Download link to original data: https://www.jianguoyun.com/p/DWcsKGUQ3p_DChjB8boEIAA
