# Supplementary material for: Immune cell infiltration-related clinical diagnostic model for Ankylosing Spondylitis
Source: Front Genet. 2022 Sep 5;13:949882. doi: 10.3389/fgene.2022.949882 (PMC9575679; doi:10.3389/fgene.2022.949882)
Supplement: Supplementary file 7 [file Table7.DOCX]

**Supplement Table 7**

Patients’ baseline information of the immunohistochemical analysis.

| Patients | Diagnosis | Gender | Age | Height | Weight | BMI |
| --- | --- | --- | --- | --- | --- | --- |
| N1 | Spinal column fracture | Male | 39 | 177 | 53 | 16.9 |
| N2 | Spinal column fracture | Male | 44 | 172 | 66 | 22.3 |
| N3 | Spinal column fracture | Male | 38 | 168 | 72 | 25.5 |
| AS1 | AS combined with kyphoscoliosis | Male | 34 | 144 | 45 | 21.7 |
| AS2 | AS combined with kyphoscoliosis | Male | 34 | 155 | 36 | 14.9 |
| AS3 | AS combined with kyphoscoliosis | Male | 43 | 145 | 65 | 30.9 |
| AS4 | AS combined with kyphoscoliosis | Male | 37 | 174 | 50 | 16.5 |
| AS5 | AS combined with kyphoscoliosis | Male | 42 | 169 | 66 | 23.1 |
