# Supplementary material for: Immune cell infiltration-related clinical diagnostic model for Ankylosing Spondylitis
Source: Front Genet. 2022 Sep 5;13:949882. doi: 10.3389/fgene.2022.949882 (PMC9575679; doi:10.3389/fgene.2022.949882)
Supplement: Supplementary file 10 [file Table2.DOCX]

**Supplement Table 2**

225 DE-miRNAs list

| miRNA id | AveExpr-AS | AveExpr-NonAS | pvalue |
| --- | --- | --- | --- |
| hsa-let-7i-3p | 2.920445216 | 3.022475271 | 0.028673468 |
| hsa-miR-101-5p | 2.922053038 | 3.461916169 | 0.001223227 |
| hsa-miR-103b | 2.910784165 | 2.85178198 | 0.006007224 |
| hsa-miR-105-5p | 2.945872206 | 2.806691658 | 0.049284566 |
| hsa-miR-10a-3p | 3.021070247 | 3.183644288 | 0.024296022 |
| hsa-miR-1184 | 2.968150843 | 2.89227993 | 0.045945608 |
| hsa-miR-1206 | 3.114386284 | 2.965143327 | 0.048939417 |
| hsa-miR-1251-3p | 2.894384784 | 2.853385976 | 0.024220128 |
| hsa-miR-1256 | 2.99932602 | 2.907083148 | 0.033883553 |
| hsa-miR-125b-2-3p | 3.523845089 | 5.337003306 | 0.047207488 |
| hsa-miR-1273c | 4.677623045 | 3.429182004 | 0.039304225 |
| hsa-miR-1273h-5p | 3.022009746 | 3.145891708 | 0.002619869 |
| hsa-miR-1276 | 3.041302505 | 2.914677691 | 0.00454408 |
| hsa-miR-1289 | 2.91215031 | 3.010223866 | 0.031299686 |
| hsa-miR-1294 | 3.009670721 | 2.919883503 | 0.029744949 |
| hsa-miR-1299 | 3.387545803 | 3.221101055 | 0.044004709 |
| hsa-miR-1302 | 3.055425748 | 2.966413013 | 0.020090429 |
| hsa-miR-1323 | 3.022944646 | 2.882341103 | 0.007494124 |
| hsa-miR-135b-3p | 2.952865376 | 2.860453927 | 0.049679203 |
| hsa-miR-138-2-3p | 2.995652689 | 2.883176611 | 0.002174298 |
| hsa-miR-140-3p | 8.730105198 | 10.99104057 | 0.048843629 |
| hsa-miR-140-5p | 8.214305702 | 10.72158956 | 0.032036042 |
| hsa-miR-151a-3p | 4.805371638 | 6.011857449 | 0.024527268 |
| hsa-miR-154-5p | 3.652074364 | 4.191185658 | 0.041120592 |
| hsa-miR-155-3p | 3.017064597 | 2.923366301 | 0.012181677 |
| hsa-miR-1587 | 7.438373125 | 6.578776866 | 0.037230343 |
| hsa-miR-15a-3p | 2.947953678 | 3.049854564 | 0.034412445 |
| hsa-miR-181c-5p | 3.573279041 | 4.512974322 | 0.043731453 |
| hsa-miR-186-5p | 5.561677884 | 6.806843209 | 0.016306909 |
| hsa-miR-1911-5p | 2.945998939 | 2.817654413 | 0.002741172 |
| hsa-miR-193b-3p | 7.205699007 | 8.353561498 | 0.040817769 |
| hsa-miR-196b-5p | 4.127299129 | 6.567417741 | 0.017699987 |
| hsa-miR-203a-3p | 3.129760472 | 2.990432081 | 0.030096293 |
| hsa-miR-20a-3p | 3.127389574 | 3.430542389 | 0.021851794 |
| hsa-miR-2114-3p | 2.949144132 | 2.844634024 | 0.015355278 |
| hsa-miR-212-3p | 3.010877584 | 3.319327385 | 0.023520535 |
| hsa-miR-224-3p | 3.078070986 | 3.68701137 | 0.039748046 |
| hsa-miR-29a-5p | 2.926607168 | 3.185714034 | 0.011448806 |
| hsa-miR-29b-1-5p | 3.138625733 | 3.295344847 | 0.021536517 |
| hsa-miR-29c-5p | 3.913043073 | 5.362489948 | 0.034171743 |
| hsa-miR-301a-3p | 4.007623471 | 5.403367598 | 0.027687041 |
| hsa-miR-302c-5p | 2.979911861 | 2.877490596 | 0.047669271 |
| hsa-miR-3074-3p | 2.897930084 | 2.970583297 | 0.026252753 |
| hsa-miR-30b-5p | 9.10850903 | 10.26559722 | 0.043234293 |
| hsa-miR-30e-3p | 4.189442832 | 5.359177783 | 0.019666623 |
| hsa-miR-3119 | 3.070166754 | 2.93329228 | 0.009038598 |
| hsa-miR-3145-3p | 2.981051899 | 3.054682047 | 0.046266486 |
| hsa-miR-3147 | 3.751784817 | 3.323223002 | 0.022809931 |
| hsa-miR-3150b-3p | 2.92597833 | 3.013232872 | 0.026155097 |
| hsa-miR-3152-3p | 3.064541847 | 2.990058711 | 0.038265674 |
| hsa-miR-3162-5p | 10.76921201 | 9.50516486 | 0.039718164 |
| hsa-miR-3178 | 2.859827511 | 2.802419484 | 0.013369976 |
| hsa-miR-3182 | 2.871330674 | 2.93237313 | 0.019194147 |
| hsa-miR-3185 | 2.874834954 | 2.956245751 | 0.04911648 |
| hsa-miR-3187-3p | 2.962334906 | 3.067538176 | 0.041850999 |
| hsa-miR-3187-5p | 3.043195128 | 2.949993832 | 0.006197512 |
| hsa-miR-320a | 6.854981582 | 7.479047298 | 0.036873212 |
| hsa-miR-320b | 7.45839456 | 8.205057658 | 0.020178401 |
| hsa-miR-320d | 8.053750135 | 8.817970553 | 0.02619139 |
| hsa-miR-320e | 7.582380204 | 8.404878139 | 0.019501926 |
| hsa-miR-330-5p | 2.937417074 | 2.799767242 | 0.011767607 |
| hsa-miR-342-5p | 3.110342212 | 3.345477472 | 0.000437305 |
| hsa-miR-3613-5p | 3.150240149 | 3.053704273 | 0.02646251 |
| hsa-miR-3615 | 2.865805044 | 2.818556931 | 0.030971951 |
| hsa-miR-3622b-5p | 3.170660074 | 3.23014872 | 0.032882467 |
| hsa-miR-365a-3p | 9.808271548 | 10.77393196 | 0.047879609 |
| hsa-miR-3678-3p | 2.918938381 | 3.01290686 | 0.023748885 |
| hsa-miR-3692-5p | 5.275717626 | 4.382664844 | 0.002747777 |
| hsa-miR-374a-5p | 7.552411278 | 8.435479814 | 0.041274362 |
| hsa-miR-374b-5p | 6.103196306 | 7.282887307 | 0.046770809 |
| hsa-miR-378j | 3.037321012 | 3.166377852 | 0.012960751 |
| hsa-miR-380-5p | 2.788074155 | 2.911619272 | 0.005246053 |
| hsa-miR-3912-3p | 2.972911102 | 2.874052356 | 0.045914978 |
| hsa-miR-3912-5p | 3.173585518 | 2.96013622 | 0.047974268 |
| hsa-miR-3916 | 2.934281918 | 2.814176803 | 0.018427454 |
| hsa-miR-3975 | 2.984020539 | 2.822296543 | 0.045329072 |
| hsa-miR-3978 | 2.865682662 | 2.928426299 | 0.001541835 |
| hsa-miR-410-5p | 3.058136666 | 2.93467332 | 0.023967724 |
| hsa-miR-423-3p | 2.974865592 | 3.110427651 | 0.049648681 |
| hsa-miR-424-3p | 4.128455002 | 3.181954482 | 0.02838548 |
| hsa-miR-4252 | 2.928202039 | 2.855615556 | 0.027216189 |
| hsa-miR-425-5p | 5.819306595 | 7.026565698 | 0.047967872 |
| hsa-miR-4281 | 12.36836501 | 11.58367557 | 0.041532058 |
| hsa-miR-432-3p | 2.827393497 | 2.916517966 | 0.026540929 |
| hsa-miR-4330 | 2.951263595 | 2.888738066 | 0.010963244 |
| hsa-miR-4428 | 8.087564859 | 6.58987035 | 0.032276152 |
| hsa-miR-4441 | 3.457574365 | 3.34133825 | 0.041827145 |
| hsa-miR-4442 | 8.703431864 | 8.14254802 | 0.004695975 |
| hsa-miR-4450 | 3.415161915 | 3.117941961 | 0.032502089 |
| hsa-miR-4478 | 9.025844707 | 7.792744425 | 0.015533031 |
| hsa-miR-4480 | 2.994429015 | 2.919327932 | 0.031144918 |
| hsa-miR-4482-3p | 2.905465766 | 3.022125226 | 0.029024099 |
| hsa-miR-4485-3p | 6.004074967 | 5.157864181 | 0.009012045 |
| hsa-miR-4499 | 7.874850621 | 7.455825236 | 0.012305259 |
| hsa-miR-449b-5p | 3.019491134 | 2.952180791 | 0.043491971 |
| hsa-miR-449c-3p | 2.968799675 | 2.822983911 | 0.007297561 |
| hsa-miR-4509 | 3.180904697 | 3.005006725 | 0.038216414 |
| hsa-miR-4515 | 5.376563413 | 4.729547423 | 0.024147477 |
| hsa-miR-4525 | 2.97775255 | 2.889210262 | 0.010074546 |
| hsa-miR-4537 | 2.961634865 | 2.845725252 | 0.039867303 |
| hsa-miR-4632-3p | 2.860358335 | 2.807019563 | 0.04089595 |
| hsa-miR-4638-5p | 2.985808382 | 2.901675613 | 0.023438055 |
| hsa-miR-4647 | 5.092627142 | 4.223992599 | 0.01376324 |
| hsa-miR-4661-3p | 2.849880631 | 2.915537815 | 0.024413294 |
| hsa-miR-4664-3p | 3.023495945 | 2.984978732 | 0.046398167 |
| hsa-miR-4671-5p | 2.921173512 | 2.859045706 | 0.039242962 |
| hsa-miR-4675 | 2.96470642 | 3.029407184 | 0.03286745 |
| hsa-miR-4684-5p | 3.014412268 | 2.839679271 | 0.036521818 |
| hsa-miR-4691-5p | 2.89563508 | 2.956870705 | 0.043706639 |
| hsa-miR-4694-3p | 2.892849116 | 2.855653267 | 0.009000482 |
| hsa-miR-4697-5p | 5.618673791 | 4.475006159 | 0.014123311 |
| hsa-miR-4704-3p | 3.00615768 | 2.90285934 | 0.042419352 |
| hsa-miR-4706 | 2.947582511 | 2.878525048 | 0.037454097 |
| hsa-miR-4713-3p | 8.054119955 | 7.459760963 | 0.016145675 |
| hsa-miR-4720-3p | 2.88983431 | 2.970531656 | 0.039951439 |
| hsa-miR-4725-3p | 3.101070597 | 2.963490911 | 0.010440106 |
| hsa-miR-4727-3p | 3.179226104 | 2.996123444 | 0.019308107 |
| hsa-miR-4728-5p | 8.094129733 | 7.147637384 | 0.040062883 |
| hsa-miR-4741 | 9.718184029 | 9.051600618 | 0.01544381 |
| hsa-miR-4746-3p | 6.330738072 | 5.444437225 | 0.001589878 |
| hsa-miR-4752 | 3.022820306 | 2.95565458 | 0.012782949 |
| hsa-miR-4755-3p | 3.959806903 | 3.80772569 | 0.044327184 |
| hsa-miR-4758-5p | 5.716041151 | 4.772578385 | 0.021190798 |
| hsa-miR-4761-5p | 2.880296582 | 2.806257311 | 0.040828646 |
| hsa-miR-4779 | 3.199487554 | 3.005885271 | 0.019746975 |
| hsa-miR-4780 | 2.930127171 | 2.867992712 | 0.026891983 |
| hsa-miR-4795-5p | 3.213712266 | 3.008174623 | 0.025361777 |
| hsa-miR-4796-3p | 2.916476801 | 3.072263439 | 0.007467135 |
| hsa-miR-4796-5p | 3.00726387 | 2.866496935 | 0.022615522 |
| hsa-miR-484 | 3.700435386 | 4.715290989 | 0.032465822 |
| hsa-miR-491-3p | 2.938280729 | 2.841903676 | 0.01110859 |
| hsa-miR-494-3p | 9.815887532 | 8.356136301 | 0.021475376 |
| hsa-miR-495-3p | 3.218254169 | 3.813567661 | 0.007648491 |
| hsa-miR-5000-3p | 2.887258317 | 2.94741948 | 0.038354979 |
| hsa-miR-5006-5p | 8.266948953 | 7.443224053 | 0.035764038 |
| hsa-miR-5009-3p | 3.003691802 | 2.922150005 | 0.047908373 |
| hsa-miR-500a-3p | 3.15233118 | 3.867489717 | 0.006314123 |
| hsa-miR-5010-5p | 4.19307036 | 3.886084153 | 0.002854989 |
| hsa-miR-501-5p | 3.207141363 | 3.409088976 | 0.008703961 |
| hsa-miR-502-3p | 3.258484956 | 3.965699625 | 0.007985649 |
| hsa-miR-502-5p | 2.951885147 | 3.199603378 | 0.028109234 |
| hsa-miR-5093 | 2.918822115 | 3.027675538 | 0.015045975 |
| hsa-miR-511-3p | 3.107605801 | 3.340782731 | 0.003842774 |
| hsa-miR-513a-5p | 6.40600609 | 4.778182123 | 0.018572281 |
| hsa-miR-515-3p | 3.00073796 | 2.882300775 | 0.00362534 |
| hsa-miR-5197-3p | 2.966328362 | 2.871088823 | 0.038345864 |
| hsa-miR-532-3p | 3.197522493 | 3.904420245 | 0.022510514 |
| hsa-miR-532-5p | 4.038655566 | 5.400964823 | 0.010255967 |
| hsa-miR-548au-3p | 2.93609282 | 2.997745911 | 0.036878951 |
| hsa-miR-548p | 3.031750328 | 2.916330387 | 0.025497363 |
| hsa-miR-548q | 3.167700656 | 3.3428348 | 0.046906804 |
| hsa-miR-552-3p | 3.022972322 | 2.901017408 | 0.006698797 |
| hsa-miR-5580-3p | 3.157056386 | 3.010212882 | 0.011694877 |
| hsa-miR-5581-5p | 6.724031106 | 6.251669012 | 0.047126876 |
| hsa-miR-5583-5p | 3.070428911 | 2.976822655 | 0.04467623 |
| hsa-miR-5588-3p | 2.834985821 | 2.946016711 | 0.008597722 |
| hsa-miR-5589-3p | 2.834673388 | 2.904210859 | 0.015963696 |
| hsa-miR-566 | 2.955912449 | 3.202757478 | 0.025460486 |
| hsa-miR-5699-3p | 2.969777277 | 2.885949044 | 0.04072217 |
| hsa-miR-574-5p | 8.552093542 | 6.423876199 | 0.035218702 |
| hsa-miR-5787 | 12.92681085 | 12.0808626 | 0.02059777 |
| hsa-miR-582-3p | 2.972451862 | 2.887239116 | 0.018192339 |
| hsa-miR-590-3p | 2.980013321 | 3.054764341 | 0.038548163 |
| hsa-miR-590-5p | 4.492649265 | 5.626140188 | 0.046712925 |
| hsa-miR-596 | 2.811651166 | 2.868483332 | 0.034376927 |
| hsa-miR-600 | 2.871200023 | 2.914959117 | 0.046648161 |
| hsa-miR-605-5p | 3.018477912 | 3.253473995 | 0.035047001 |
| hsa-miR-6077 | 2.823782206 | 2.933731795 | 0.009083378 |
| hsa-miR-6082 | 2.923957094 | 2.846277349 | 0.036293101 |
| hsa-miR-6129 | 4.627170983 | 4.01682589 | 0.00116531 |
| hsa-miR-613 | 2.924542317 | 2.841791385 | 0.003028263 |
| hsa-miR-6133 | 3.649750273 | 3.349941423 | 0.004326796 |
| hsa-miR-614 | 3.009977134 | 2.904896271 | 0.020105791 |
| hsa-miR-617 | 3.033112885 | 3.065479815 | 0.027431439 |
| hsa-miR-628-5p | 3.045935941 | 3.303374894 | 0.008615626 |
| hsa-miR-632 | 2.935234872 | 2.890369788 | 0.043187031 |
| hsa-miR-647 | 2.883485484 | 2.811310713 | 0.013907013 |
| hsa-miR-6502-3p | 2.921223814 | 3.001575292 | 0.016530078 |
| hsa-miR-6504-3p | 2.852354745 | 2.902217767 | 0.002819085 |
| hsa-miR-6510-5p | 10.08793803 | 9.308703545 | 0.00431904 |
| hsa-miR-660-5p | 5.023877729 | 6.278352179 | 0.001907218 |
| hsa-miR-664a-3p | 3.198076232 | 3.856946243 | 0.039186322 |
| hsa-miR-670-5p | 3.099330043 | 2.92141179 | 0.043876354 |
| hsa-miR-6720-3p | 2.991262447 | 3.052381402 | 0.048934481 |
| hsa-miR-6721-5p | 2.906642385 | 3.051662578 | 0.015299133 |
| hsa-miR-6724-5p | 8.800436434 | 8.255356451 | 0.013754057 |
| hsa-miR-6733-3p | 3.041796144 | 2.891636815 | 0.045722412 |
| hsa-miR-6735-3p | 2.861194297 | 2.84085108 | 0.031955696 |
| hsa-miR-6754-3p | 2.874098601 | 2.80481548 | 0.036052164 |
| hsa-miR-6756-5p | 6.377942014 | 6.115805502 | 0.007838189 |
| hsa-miR-6769b-5p | 10.26788776 | 9.25011368 | 0.02883976 |
| hsa-miR-6779-5p | 5.204723104 | 4.889891311 | 0.015763745 |
| hsa-miR-6780b-5p | 9.243701337 | 8.37523217 | 0.013430105 |
| hsa-miR-6785-5p | 11.55394125 | 9.242288069 | 0.038108145 |
| hsa-miR-6803-3p | 2.844896268 | 2.892944337 | 0.048117338 |
| hsa-miR-6804-3p | 2.878192846 | 2.941713271 | 0.048899877 |
| hsa-miR-6816-3p | 2.910620422 | 2.843237975 | 0.044097254 |
| hsa-miR-6832-5p | 3.23585222 | 3.025493906 | 0.026492358 |
| hsa-miR-6852-3p | 2.839360138 | 2.903686163 | 0.048296744 |
| hsa-miR-6852-5p | 3.036776978 | 2.896967823 | 0.005656751 |
| hsa-miR-6853-5p | 3.042047056 | 2.939562077 | 0.002968408 |
| hsa-miR-6864-5p | 2.992102153 | 2.916239985 | 0.020389985 |
| hsa-miR-6867-5p | 8.905837779 | 8.226231579 | 0.015218121 |
| hsa-miR-6868-5p | 3.011027335 | 2.893202984 | 0.013857096 |
| hsa-miR-6875-5p | 10.41141687 | 9.31012594 | 0.049974865 |
| hsa-miR-6879-5p | 9.199771523 | 8.547764641 | 0.013003419 |
| hsa-miR-6888-5p | 2.999553687 | 2.913020455 | 0.043225653 |
| hsa-miR-6893-5p | 9.767260671 | 8.817980981 | 0.049789651 |
| hsa-miR-7107-5p | 11.72079093 | 10.70194727 | 0.043617024 |
| hsa-miR-7-1-3p | 2.978523758 | 3.206730794 | 0.041649172 |
| hsa-miR-7159-3p | 2.978316643 | 3.138255825 | 0.026105946 |
| hsa-miR-760 | 3.045386846 | 3.303964314 | 0.048626412 |
| hsa-miR-764 | 2.815262742 | 2.90075254 | 0.006256695 |
| hsa-miR-767-5p | 2.947682764 | 3.003016936 | 0.010381466 |
| hsa-miR-770-5p | 2.914085945 | 3.027434665 | 0.04874972 |
| hsa-miR-7843-3p | 2.918522717 | 2.812443113 | 0.039204896 |
| hsa-miR-7850-5p | 3.075948812 | 2.929959355 | 0.047347074 |
| hsa-miR-7852-3p | 3.073318425 | 2.970419487 | 0.028929054 |
| hsa-miR-7976 | 2.974824094 | 2.833157057 | 0.002791903 |
| hsa-miR-8072 | 7.471353345 | 6.867367142 | 0.039022034 |
| hsa-miR-885-3p | 3.118328323 | 3.027690766 | 0.037375394 |
| hsa-miR-887-5p | 2.872039503 | 2.933231919 | 0.016950533 |
| hsa-miR-892a | 2.963632626 | 2.885906321 | 0.002574653 |
| hsa-miR-98-5p | 5.737945077 | 6.922107755 | 0.001995492 |
| hsa-miR-99a-3p | 2.923280102 | 3.168672158 | 0.010155976 |
