# Supplementary material for: Immune cell infiltration-related clinical diagnostic model for Ankylosing Spondylitis
Source: Front Genet. 2022 Sep 5;13:949882. doi: 10.3389/fgene.2022.949882 (PMC9575679; doi:10.3389/fgene.2022.949882)
Supplement: Supplementary file 11 [file Table5.DOCX]

**Supplement Table 5**

38 DE-mRNAs in miRNA-mRNA network

| id |
| --- |
| *CDC42SE1* |
| *LIMD2* |
| *MYO1F* |
| *PIGA* |
| *KCTD14* |
| *LEFTY2* |
| *GAB1* |
| *PABPC1L2A* |
| *SYT9* |
| *CITED2* |
| *PAX3* |
| *SKIDA1* |
| *LYN* |
| *PDSS1* |
| *RTN4R* |
| *RFESD* |
| *PURG* |
| *CCL1* |
| *HIST1H2AK* |
| *HIST1H3H* |
| *HIST2H3C* |
| *DLX5* |
| *HMBS* |
| *SYNPR* |
| *DLX3* |
| *GPC6* |
| *LPAR2* |
| *ANK3* |
| *ARID4A* |
| *ELL2* |
| *KIAA1462* |
| *MPPED2* |
| *NMT2* |
| *PARD6B* |
| *PGRMC2* |
| *PLAG1* |
| *SIX3* |
| *ZC3H4* |
