# Supplementary material for: Immune cell infiltration-related clinical diagnostic model for Ankylosing Spondylitis
Source: Front Genet. 2022 Sep 5;13:949882. doi: 10.3389/fgene.2022.949882 (PMC9575679; doi:10.3389/fgene.2022.949882)
Supplement: Supplementary file 13 [file Table3.DOCX]

**Supplement Table 3**

406 DE-mRNAs list

| mRNA id | AveExpr-AS | AveExpr-NonAS | pvalue |
| --- | --- | --- | --- |
| *ABCA5* | 2.732482836 | 5.085679396 | 0.001298999 |
| *ABHD13* | 2.393255198 | 3.286108064 | 0.008996306 |
| *ABI3BP* | 8.838009452 | 10.91731201 | 0.010703142 |
| *ADAM21* | 2.486979539 | 2.387098063 | 0.040450022 |
| *ADAM8* | 5.199861385 | 2.600507562 | 0.014417664 |
| *ADAMTSL2* | 9.674503498 | 11.01471165 | 0.03397763 |
| *ADGRE1* | 5.475155439 | 3.249062404 | 0.033037144 |
| *ADGRG1* | 6.470948411 | 5.968787324 | 0.021247207 |
| *ADNP* | 4.242013033 | 5.496724117 | 0.048814109 |
| *AK9* | 2.645851798 | 3.971737065 | 0.015689502 |
| *ALG10B* | 5.977829168 | 7.022733017 | 0.010894285 |
| *AMIGO3* | 6.989600958 | 7.632207564 | 0.047433618 |
| *ANK3* | 2.314021589 | 4.156977766 | 0.016938033 |
| *ANKRD13C* | 2.511105657 | 4.530756834 | 0.002853386 |
| *ANKRD35* | 10.0177667 | 11.51570464 | 0.032187757 |
| *ANKRD6* | 2.769193421 | 4.073107073 | 0.015063048 |
| *APOBEC3H* | 7.16989463 | 6.001668861 | 0.003598251 |
| *ARHGAP9* | 9.27431488 | 7.304756834 | 0.04575298 |
| *ARHGEF5* | 7.407656291 | 8.021959911 | 0.018899574 |
| *ARID4A* | 6.042332523 | 6.184420684 | 0.037431821 |
| *ARMC12* | 3.088941688 | 4.450444752 | 0.045199292 |
| *ARSG* | 3.999435357 | 5.201632233 | 0.025910173 |
| *ART1* | 2.379651629 | 3.531561912 | 0.024478621 |
| *ASS1* | 9.185217341 | 10.29914815 | 0.033897668 |
| *ATAT1* | 7.833350553 | 7.34793032 | 0.039800068 |
| *ATG12* | 2.833138078 | 4.791083 | 0.010790559 |
| *BAG1* | 5.975168811 | 6.988749961 | 0.008607677 |
| *BBX* | 9.412459346 | 10.18122617 | 0.048169797 |
| *BCAN* | 3.517049512 | 5.441873092 | 0.03391848 |
| *BLID* | 3.039725871 | 5.101403447 | 0.040820004 |
| *BROX* | 5.026370296 | 5.687702899 | 0.005182742 |
| *C10orf111* | 2.387768328 | 3.046831834 | 0.010531013 |
| *C12orf60* | 2.930696866 | 4.986253972 | 0.013780131 |
| *C14orf159* | 4.094673477 | 4.816577158 | 0.021911335 |
| *C17orf53* | 7.326208947 | 5.617786028 | 0.027094381 |
| *C18orf54* | 4.678151068 | 5.672835696 | 0.011079601 |
| *C1QTNF3* | 5.108519273 | 7.375195016 | 0.031486729 |
| *C2CD3* | 2.767538468 | 4.320509011 | 0.043827284 |
| *C3orf35* | 2.387704657 | 3.227548275 | 0.03721397 |
| *C4orf45* | 2.483495739 | 2.39460105 | 0.048175468 |
| *C9orf135* | 2.46511611 | 2.380372809 | 0.048907694 |
| *CACHD1* | 7.20851351 | 9.312559069 | 0.044755118 |
| *CACNA1C* | 5.40841169 | 3.966383753 | 0.007933643 |
| *CACNA1G* | 4.601083025 | 3.324350686 | 0.022812168 |
| *CAPS* | 3.148276647 | 6.009346902 | 0.030365587 |
| *CARD8* | 4.466402345 | 3.393131157 | 0.008124294 |
| *CATSPER3* | 2.38950244 | 4.142421836 | 0.003596754 |
| *CCDC102B* | 9.873804068 | 9.178820722 | 0.040653018 |
| *CCDC110* | 5.117009978 | 6.578434497 | 0.009296892 |
| *CCDC171* | 3.060881371 | 4.554212365 | 0.034426308 |
| *CCK* | 2.499353179 | 4.844409811 | 0.003463911 |
| *CCL1* | 5.931993883 | 4.312702006 | 0.027497021 |
| *CCL26* | 8.860922467 | 7.238007042 | 0.00901129 |
| *CCL28* | 4.018330679 | 6.202475983 | 0.046395089 |
| *CCNO* | 4.351965156 | 6.401824952 | 0.044905104 |
| *CD27* | 7.071164705 | 5.743326653 | 0.034428951 |
| *CD300LF* | 5.26731653 | 3.310005483 | 0.003896129 |
| *CD37* | 5.68913598 | 3.7206129 | 0.021315081 |
| *CD9* | 12.97760488 | 14.18642988 | 0.046983736 |
| *CDC42SE1* | 6.971784589 | 5.545913055 | 0.049050734 |
| *CDC6* | 5.302468119 | 4.282066614 | 0.029866204 |
| *CDH12* | 4.475698641 | 3.039737148 | 0.033501402 |
| *CDH24* | 7.168919364 | 6.377972221 | 0.007844822 |
| *CDO1* | 11.37004709 | 14.18522577 | 0.000425155 |
| *CEP126* | 7.023628093 | 9.411998166 | 0.011204214 |
| *CFAP221* | 6.619483895 | 7.562759664 | 0.017246753 |
| *CFAP53* | 2.350859567 | 4.02573497 | 0.000197206 |
| *CHD5* | 3.000054144 | 5.758127292 | 0.045873088 |
| *CILP2* | 9.571008193 | 12.14547897 | 0.037491083 |
| *CITED2* | 9.511071381 | 11.00488718 | 0.034671473 |
| *CNN2* | 6.906580122 | 6.008524778 | 0.005225852 |
| *CNTNAP4* | 7.215147234 | 7.883351896 | 0.000744373 |
| *COL9A1* | 2.639885716 | 4.379253231 | 0.014484552 |
| *COLGALT2* | 9.639841751 | 12.42224777 | 0.018577827 |
| *CPNE2* | 10.82961061 | 11.54030042 | 0.028329507 |
| *CPTP* | 9.601536796 | 10.66218655 | 0.023374558 |
| *CRHBP* | 2.804033659 | 4.505354249 | 0.028751341 |
| *CRHR2* | 2.609859394 | 3.891354236 | 0.043767951 |
| *CSH2* | 4.470839597 | 3.316548669 | 0.012313892 |
| *CT47B1* | 2.464783845 | 2.380234829 | 0.049000032 |
| *CTRB1* | 2.620936344 | 5.26926983 | 0.026159389 |
| *CUBN* | 3.566989202 | 5.972900183 | 0.037439585 |
| *CXCR5* | 2.583599216 | 3.592357067 | 0.040810997 |
| *CXorf57* | 3.172339133 | 4.978496731 | 0.038370061 |
| *CXXC4* | 5.610418433 | 8.045597001 | 0.010490869 |
| *DCN* | 12.66735933 | 14.73518306 | 0.049433582 |
| *DDA1* | 6.388040503 | 4.985393045 | 0.012479772 |
| *DENND2C* | 2.797202979 | 4.386983335 | 0.006022554 |
| *DLGAP5* | 6.469880308 | 5.388862689 | 0.030322313 |
| *DLX3* | 4.364697978 | 2.652144762 | 0.005190618 |
| *DLX5* | 7.475931589 | 9.400647523 | 0.024772863 |
| *DNAJB13* | 2.698886267 | 3.91167803 | 0.034676636 |
| *DNM1L* | 2.481347637 | 2.385249127 | 0.036449448 |
| *DOCK5* | 5.674468303 | 4.02818456 | 0.012908123 |
| *DOT1L* | 6.944161047 | 6.426849424 | 0.049855128 |
| *DPCR1* | 2.320325153 | 2.862056072 | 0.009451816 |
| *DRC7* | 5.264350255 | 3.25225931 | 0.027665959 |
| *EEF1A2* | 3.494506124 | 8.463235454 | 0.033826422 |
| *EFCAB5* | 6.047645587 | 2.924119145 | 0.019056553 |
| *EFCAB7* | 2.617059471 | 4.037743418 | 0.005521662 |
| *ELL2* | 8.888716513 | 10.10495821 | 0.045430752 |
| *ERMN* | 3.016132647 | 5.571927814 | 0.042176009 |
| *ERVMER34-1* | 2.424601719 | 6.572394841 | 0.000174062 |
| *EVI2B* | 8.315519527 | 3.591669122 | 0.019016668 |
| *EVX2* | 2.537740239 | 6.10601925 | 0.029062225 |
| *EXOC3L1* | 4.555415795 | 11.73402479 | 0.031144757 |
| *FAM178A* | 13.98477746 | 7.521475172 | 0.046829146 |
| *FAM45A* | 7.160296866 | 3.004364384 | 0.03220436 |
| *FAM72C* | 2.351880251 | 11.07751617 | 0.004951175 |
| *FAXDC2* | 9.080431691 | 6.104278079 | 0.044748022 |
| *FCN3* | 7.101442021 | 4.232902283 | 0.002046735 |
| *FGF22* | 5.562445586 | 5.081682181 | 0.021455768 |
| *FGF9* | 2.930343706 | 5.323496602 | 0.00816044 |
| *FGFBP3* | 4.20985538 | 13.9592247 | 0.033389929 |
| *FMOD* | 10.56013186 | 2.357699123 | 0.012967478 |
| *FNDC7* | 2.47422319 | 5.153712427 | 0.038083827 |
| *FOXF2* | 2.706646643 | 8.222859511 | 0.019337654 |
| *FOXP4* | 6.616702171 | 3.275014777 | 0.035738676 |
| *FTH1P18* | 5.070043406 | 4.406233593 | 0.02634182 |
| *FUT7* | 5.870065563 | 7.145000161 | 0.039551328 |
| *GAB1* | 6.353996491 | 11.76821918 | 0.027304523 |
| *GALNT15* | 9.740990657 | 8.3526824 | 0.029727176 |
| *GBP2* | 7.749471871 | 4.010561629 | 0.005850542 |
| *GCDH* | 2.671062907 | 2.381873405 | 0.045798001 |
| *GDF9* | 2.470424611 | 6.708833337 | 0.044864789 |
| *GIPC2* | 5.02350073 | 3.057163188 | 0.042959371 |
| *GJC3* | 2.468796032 | 9.078961219 | 0.024706423 |
| *GLCE* | 10.16019927 | 2.424042248 | 0.038114194 |
| *GPC6* | 4.52688894 | 4.889473503 | 0.014848672 |
| *GRIN3A* | 3.621660006 | 3.878546801 | 0.025070731 |
| *GRK4* | 2.786002829 | 10.9640535 | 0.013229741 |
| *H1FX* | 10.63738365 | 5.967000395 | 0.034027412 |
| *HEPACAM* | 3.723040023 | 4.960324988 | 0.049986248 |
| *HERC1* | 5.605018046 | 4.020738186 | 0.02812308 |
| *HESX1* | 2.585186253 | 11.51929865 | 0.005913916 |
| *HIGD2A* | 11.82989512 | 3.114521279 | 0.041263113 |
| *HIST1H2AK* | 5.209388898 | 2.624232251 | 0.029612677 |
| *HIST1H3H* | 4.004451306 | 3.303553175 | 0.00649038 |
| *HIST2H3C* | 7.089765864 | 8.543497692 | 0.030855624 |
| *HIST2H4A* | 9.406447066 | 4.25712937 | 0.02504699 |
| *HMBS* | 5.382623158 | 3.846554008 | 0.030676871 |
| *HOMEZ* | 2.663129392 | 7.904376425 | 0.012839413 |
| *HOXA5* | 6.351292949 | 10.98970207 | 0.023162807 |
| *HOXD9* | 8.920371228 | 9.633039444 | 0.034331584 |
| *HSPA2* | 8.98729123 | 3.583275912 | 0.013791013 |
| *HTR3D* | 2.390920255 | 6.652613758 | 0.008962779 |
| *HVCN1* | 7.449342815 | 5.872400672 | 0.011820532 |
| *IL17RC* | 6.443602813 | 2.573780118 | 0.023147941 |
| *IL2RG* | 3.671574203 | 7.251259301 | 0.034306067 |
| *IL7R* | 8.591458481 | 4.361530626 | 0.007907204 |
| *INTS2* | 3.717966603 | 5.906907376 | 0.034071789 |
| *INTS8* | 4.665693069 | 7.418064404 | 0.040494421 |
| *IPP* | 6.309044163 | 9.888071928 | 0.016415533 |
| *ISM1* | 7.510242701 | 3.217254366 | 0.011559414 |
| *ITK* | 5.36064044 | 12.22482455 | 0.041468769 |
| *ITPRIPL2* | 11.341678 | 7.776949553 | 0.048813156 |
| *KAT2B* | 6.197688429 | 8.668065647 | 0.043253452 |
| *KCNH3* | 9.365985492 | 3.477583513 | 0.025745911 |
| *KCNJ14* | 5.208113902 | 3.644043607 | 0.031347491 |
| *KCNK17* | 2.408145015 | 3.918462983 | 0.034006195 |
| *KCNV2* | 2.55474927 | 3.7053961 | 0.027171926 |
| *KCTD14* | 2.437162847 | 8.044795279 | 0.017229823 |
| *KCTD15* | 8.602119413 | 3.287584303 | 0.036990978 |
| *KCTD7* | 2.405257311 | 10.29084693 | 0.003893837 |
| *KDELC2* | 9.053137584 | 4.634589949 | 0.043090806 |
| *KIAA1324L* | 2.779675458 | 4.97558702 | 0.003692609 |
| *KIAA1456* | 3.593084935 | 11.09343156 | 0.026792999 |
| *KIAA1462* | 10.50328367 | 7.848654028 | 0.020052654 |
| *KIAA2018* | 6.788072395 | 4.45240208 | 0.007300636 |
| *KIAA2022* | 2.872129039 | 8.894593204 | 0.010110607 |
| *KIF21A* | 9.641938974 | 3.285572349 | 0.005349355 |
| *KLHL10* | 2.408217662 | 8.502271323 | 0.041946537 |
| *KMT2A* | 7.200686843 | 3.106912897 | 0.030281436 |
| *KRTAP10-2* | 7.565130335 | 3.062869933 | 0.034406547 |
| *KRTAP10-9* | 7.466979394 | 9.357944138 | 0.001494736 |
| *KRTAP19-5* | 3.997450444 | 7.093707796 | 0.035049828 |
| *KRTAP29-1* | 3.564201808 | 5.595618766 | 0.046584478 |
| *KRTCAP3* | 2.395078929 | 4.677665488 | 0.024178748 |
| *LANCL3* | 4.971896577 | 11.46095986 | 0.009800788 |
| *LAT* | 10.47328727 | 7.35977887 | 0.036021004 |
| *LCA5* | 5.618303003 | 3.010094312 | 0.03427708 |
| *LCE1D* | 6.759115868 | 3.932760346 | 0.037243703 |
| *LEF1* | 6.336784552 | 5.773999876 | 0.041047642 |
| *LEFTY2* | 7.795382559 | 4.623895315 | 0.041873222 |
| *LENG1* | 6.574046814 | 6.497901821 | 0.031040892 |
| *LIMD2* | 4.612436026 | 10.51928838 | 0.022640573 |
| *LIN7A* | 5.09760929 | 4.421247924 | 0.025627847 |
| *LINC01272* | 8.470153354 | 3.73827927 | 0.036363578 |
| *LIPT2* | 2.845372845 | 10.03368495 | 0.048181716 |
| *LMBRD1* | 7.476698205 | 6.101687181 | 0.03583048 |
| *LMO2* | 9.355873325 | 5.122741725 | 0.032260099 |
| *LOC728485* | 5.423473337 | 3.357935087 | 0.029970114 |
| *LOXL3* | 2.417150002 | 2.299048106 | 0.009522518 |
| *LOXL4* | 7.039147112 | 2.380830389 | 0.048246669 |
| *LPAR2* | 7.732737407 | 11.15738201 | 0.012154369 |
| *LRFN5* | 2.47974287 | 2.787746488 | 0.047866776 |
| *LRP2BP* | 2.593699337 | 6.020699304 | 0.032047745 |
| *LRRIQ4* | 2.29455706 | 3.542754388 | 0.049322506 |
| *LRRTM2* | 2.467057563 | 8.24084731 | 0.048554122 |
| *LTBP1* | 10.2881293 | 8.180753365 | 0.003349765 |
| *LYN* | 3.968422245 | 6.174263606 | 0.025741712 |
| *LZTS3* | 6.254203991 | 8.329704266 | 0.034372817 |
| *MAT1A* | 2.355066663 | 7.507830932 | 0.037823354 |
| *MAVS* | 7.162610934 | 7.828687591 | 0.049604648 |
| *MC5R* | 9.237572608 | 2.689602774 | 0.026428346 |
| *MED12L* | 3.832800286 | 11.00987151 | 0.023704816 |
| *MED13L* | 7.548996642 | 2.707969961 | 0.027095047 |
| *MIEF2* | 6.290121662 | 7.23787849 | 0.042931897 |
| *MIS18A* | 7.346393906 | 13.00681433 | 0.041545056 |
| *MOGAT2* | 4.480715889 | 12.70962753 | 0.010971495 |
| *MPP6* | 9.685594537 | 4.150059076 | 0.045449632 |
| *MPPED2* | 2.361736199 | 10.23649473 | 0.002767522 |
| *MRPL19* | 5.855738692 | 3.549585922 | 0.016331545 |
| *MT1H* | 10.85401559 | 3.931344522 | 0.021165072 |
| *MT1X* | 8.838962643 | 2.50190589 | 0.030586788 |
| *MYEOV* | 5.84904727 | 7.664873134 | 0.00524593 |
| *MYL3* | 4.23455751 | 5.368363543 | 0.016669285 |
| *MYO1F* | 5.84778533 | 2.926511721 | 0.024585934 |
| *NARS2* | 2.663061218 | 7.944977731 | 0.022088719 |
| *NCAPH* | 4.148887464 | 3.151117539 | 0.046322008 |
| *NMT2* | 6.261159617 | 5.08204002 | 0.042505012 |
| *NOG* | 3.021581737 | 2.419114647 | 0.034186229 |
| *NOS1AP* | 4.114659388 | 3.100114603 | 0.033598432 |
| *NPIPA2* | 7.060335679 | 8.135107909 | 0.039457883 |
| *NRXN1* | 2.4665174 | 2.391330489 | 0.026614826 |
| *NTNG2* | 6.385818156 | 4.050224066 | 0.004920638 |
| *NUTM2G* | 4.058952651 | 6.035956463 | 0.005487643 |
| *OGDHL* | 5.748193147 | 11.87646049 | 0.017683784 |
| *OR10A5* | 5.326359221 | 3.407990286 | 0.046289202 |
| *OR52B4* | 2.502199913 | 6.517878414 | 0.033566067 |
| *PABPC1L2A* | 2.916602735 | 5.325512533 | 0.043831858 |
| *PABPC3* | 4.967076872 | 4.000221698 | 0.016428048 |
| *PAMR1* | 8.815719403 | 6.616261268 | 0.019654824 |
| *PARD6B* | 2.340882434 | 6.051087329 | 0.030196358 |
| *PARVG* | 7.187326287 | 3.930996306 | 0.022515488 |
| *PATE3* | 6.348303658 | 4.946502243 | 0.000563628 |
| *PATL2* | 5.610545815 | 5.154221954 | 0.033854566 |
| *PAX1* | 7.576373968 | 8.010434827 | 0.01094492 |
| *PAX3* | 2.342808468 | 9.767390954 | 0.026972113 |
| *PAX7* | 2.34878291 | 8.873132618 | 0.036851892 |
| *PCDH8* | 2.727198124 | 2.538836762 | 0.00659241 |
| *PDSS1* | 5.662744711 | 3.503326419 | 0.01410061 |
| *PEF1* | 7.715657549 | 4.441211664 | 0.045741991 |
| *PGRMC2* | 8.723002229 | 7.123341654 | 0.039823316 |
| *PHF10* | 8.171865488 | 4.586203867 | 0.023897734 |
| *PIGA* | 4.373451815 | 4.470615435 | 0.040560779 |
| *PJA1* | 4.331557871 | 7.313343782 | 0.017108178 |
| *PLAG1* | 2.474995085 | 7.951735668 | 0.020111781 |
| *PLCB2* | 9.24416843 | 5.050330913 | 0.029519553 |
| *PLCB4* | 2.780785981 | 6.006471312 | 0.014333998 |
| *PLCD3* | 2.592090611 | 4.014537022 | 0.007836168 |
| *PLCD4* | 6.280797608 | 3.990512007 | 0.004127703 |
| *PLGLB1* | 7.41132045 | 6.354047656 | 0.036151786 |
| *PLGLB2* | 3.637403056 | 7.244375885 | 0.032354994 |
| *PLSCR3* | 6.696492186 | 3.49414942 | 0.003897078 |
| *PNPLA5* | 2.441238764 | 7.061385273 | 0.001037059 |
| *POLR3F* | 2.457389413 | 10.73725368 | 0.018923925 |
| *POT1* | 6.058142586 | 3.679360139 | 0.016225981 |
| *PPP1R3G* | 6.653414511 | 3.507270524 | 0.00860835 |
| *PREPL* | 2.356054674 | 13.26279392 | 0.007257439 |
| *PRIM1* | 6.430276197 | 6.26138872 | 0.030906789 |
| *PSMG2* | 10.34877747 | 6.450230136 | 0.019074062 |
| *PTBP3* | 4.725008741 | 8.805616407 | 0.042683865 |
| *PTCRA* | 6.110701136 | 4.575403496 | 0.032338359 |
| *PTGIS* | 10.98382756 | 3.242047263 | 0.027889044 |
| *PTPRH* | 2.999177236 | 11.61575148 | 0.013217659 |
| *PURG* | 7.317778071 | 12.06591781 | 0.01796714 |
| *PYGL* | 8.408610303 | 3.859815623 | 0.013437132 |
| *RAB40AL* | 2.870749064 | 8.410550658 | 0.037622266 |
| *RANBP17* | 4.103662445 | 3.882946791 | 0.010553097 |
| *RAVER1* | 11.00870427 | 4.66649842 | 0.024195653 |
| *RBP4* | 10.16530138 | 4.875255245 | 0.021375673 |
| *RCVRN* | 2.600256132 | 5.901214244 | 0.018828486 |
| *REEP2* | 7.128482078 | 7.817595797 | 0.044257413 |
| *RFESD* | 4.989716928 | 5.044016388 | 0.02917181 |
| *RIPPLY2* | 2.741017226 | 3.942543666 | 0.014157589 |
| *RND2* | 2.860424601 | 6.590229492 | 0.045813503 |
| *RNF175* | 3.510533453 | 2.514254608 | 0.049717738 |
| *RPP25* | 8.799715516 | 4.155104987 | 0.033525416 |
| *RPUSD4* | 4.096308708 | 6.028240873 | 0.030645405 |
| *RRAGD* | 2.648256626 | 12.89681135 | 0.044736652 |
| *RSG1* | 5.446194467 | 8.944583953 | 0.042514398 |
| *RSPO4* | 3.861517158 | 2.452092724 | 0.000512203 |
| *RTN4R* | 5.708627886 | 2.873546433 | 0.008390476 |
| *RUNDC1* | 5.541548878 | 5.509911459 | 0.049669018 |
| *S100A13* | 11.66791206 | 4.634602808 | 0.042797764 |
| *SAP25* | 9.906990933 | 7.491793754 | 0.020153872 |
| *SCGB1D4* | 4.383623036 | 2.999779931 | 0.034161944 |
| *SCIMP* | 4.870330667 | 6.241040386 | 0.023039269 |
| *SCNN1A* | 3.489739716 | 3.542902296 | 0.042471305 |
| *SERHL2* | 3.873175019 | 2.45034161 | 0.040558541 |
| *SETMAR* | 7.89938403 | 3.767430926 | 0.037058776 |
| *SHANK2* | 5.663229691 | 8.398359297 | 0.047931452 |
| *SHBG* | 5.386375832 | 3.974336181 | 0.035814221 |
| *SI* | 6.084972152 | 4.211400231 | 0.015615569 |
| *SIGLEC7* | 5.215693654 | 2.381536804 | 0.02274656 |
| *SIRPB2* | 5.385931655 | 3.035682271 | 0.038690629 |
| *SIT1* | 9.404444687 | 8.014341127 | 0.022421263 |
| *SIX3* | 2.690278983 | 4.086266223 | 0.024909197 |
| *SKIDA1* | 2.517020454 | 6.549713087 | 0.002576116 |
| *SLC17A8* | 2.469084467 | 5.042750586 | 0.044193352 |
| *SLC34A2* | 2.480369009 | 8.435654612 | 0.008602133 |
| *SLC38A10* | 7.390240896 | 7.495121915 | 0.048075159 |
| *SLC5A11* | 2.760202346 | 7.119977614 | 0.01799473 |
| *SMARCA2* | 5.718051273 | 10.45878482 | 0.048453513 |
| *SNAI3* | 6.167375415 | 5.925440627 | 0.013143224 |
| *SP4* | 7.002457413 | 3.590478626 | 0.041282572 |
| *SPECC1* | 6.744943314 | 6.506385199 | 0.029317269 |
| *SPECC1L* | 5.894198548 | 11.21261499 | 0.048114459 |
| *SPEN* | 9.733432778 | 4.592366135 | 0.041195084 |
| *SPINK2* | 6.572835691 | 2.382320843 | 0.043736402 |
| *SPOPL* | 2.300206088 | 5.073335452 | 0.001275816 |
| *STAG2* | 5.617327073 | 3.707567806 | 0.041200491 |
| *STEAP2* | 8.603296176 | 2.913782512 | 0.039119856 |
| *STON1-GTF2A1L* | 2.417786115 | 3.925857911 | 0.027456007 |
| *SYNPR* | 2.471726089 | 5.549258406 | 0.048968004 |
| *SYT17* | 2.867361617 | 4.437730495 | 0.027608685 |
| *SYT9* | 2.525310678 | 3.208689179 | 0.030036831 |
| *TACC3* | 6.00271 | 4.660946807 | 0.009539712 |
| *TAS2R10* | 2.432504256 | 3.257036239 | 0.015560204 |
| *TBC1D12* | 4.618038609 | 5.641817635 | 0.002049221 |
| *TBC1D19* | 3.081876757 | 4.669160768 | 0.032857551 |
| *TBL1Y* | 2.309080109 | 9.975175195 | 0.047534452 |
| *TBX5* | 2.401145941 | 15.37942738 | 0.031558488 |
| *TCF3* | 3.960483862 | 3.843268523 | 0.026073564 |
| *TCP11L2* | 4.958991607 | 7.88224295 | 0.013348079 |
| *TFAP2D* | 5.404244043 | 4.581707863 | 0.012772055 |
| *TFDP2* | 8.584395112 | 2.910500875 | 0.031200853 |
| *THBS4* | 12.58439381 | 6.667900329 | 0.024580198 |
| *TLR9* | 4.381722021 | 3.401843899 | 0.042607154 |
| *TMEM106B* | 7.133961055 | 9.823129103 | 0.042944737 |
| *TMEM5* | 2.774674087 | 2.370848035 | 0.04065946 |
| *TMEM53* | 2.336676058 | 3.609446297 | 0.019999189 |
| *TMEM59L* | 7.838807348 | 7.011662216 | 0.026104719 |
| *TMEM67* | 2.582816609 | 8.997605252 | 0.024839317 |
| *TNFRSF11B* | 5.498126437 | 6.696629324 | 0.025736717 |
| *TPPP2* | 2.525972021 | 5.909639537 | 0.031621489 |
| *TRAT1* | 5.15508285 | 8.911667882 | 0.02265542 |
| *TRIM15* | 7.745511042 | 5.985633565 | 0.048079673 |
| *TRIM65* | 8.481223148 | 3.610151039 | 0.024309037 |
| *TRIP11* | 5.578206385 | 5.880467979 | 0.00173099 |
| *TRPM2* | 7.054754919 | 5.386125268 | 0.048869363 |
| *TRPS1* | 7.582599919 | 4.042568274 | 0.039900724 |
| *TRPV2* | 7.140110792 | 11.07461665 | 0.032117539 |
| *TSC22D1* | 2.354170833 | 5.947694384 | 0.017406937 |
| *TSPYL5* | 5.283804294 | 9.665387771 | 0.046909727 |
| *TSSK3* | 3.823582358 | 3.122058131 | 0.043735571 |
| *TTC30A* | 2.336056577 | 2.670794253 | 0.0245051 |
| *TTN* | 4.145936468 | 5.591596575 | 0.0423339 |
| *TYMP* | 7.399369591 | 3.891269742 | 0.039142664 |
| *USP13* | 8.047580462 | 3.443592839 | 0.046208402 |
| *USP50* | 2.333863894 | 7.984399745 | 0.023774244 |
| *UVSSA* | 4.022789103 | 5.353328098 | 0.029382038 |
| *VWC2* | 3.116907443 | 3.520325581 | 0.036728604 |
| *WDR5B* | 2.331183651 | 3.277307575 | 0.002114956 |
| *WDR78* | 2.370179379 | 7.588961755 | 0.047483693 |
| *WDSUB1* | 6.846154038 | 4.828647706 | 0.043848145 |
| *WFIKKN2* | 2.958113134 | 7.56504172 | 0.020173175 |
| *WNT1* | 4.706945112 | 10.7339907 | 0.049027116 |
| *WNT4* | 2.332574562 | 5.955353572 | 0.046576655 |
| *WRAP53* | 8.631570947 | 9.98935771 | 0.024773708 |
| *WWC2* | 3.014760006 | 11.36635025 | 0.045074906 |
| *XRCC6BP1* | 5.726264544 | 7.077109501 | 0.039774592 |
| *ZBTB20* | 8.938268121 | 3.467611618 | 0.033033658 |
| *ZBTB37* | 6.67949606 | 8.000381856 | 0.001425869 |
| *ZBTB45* | 9.785976769 | 6.493494778 | 0.023724217 |
| *ZC3H4* | 10.86028625 | 7.433131825 | 0.037005686 |
| *ZCCHC5* | 4.451457289 | 4.34882134 | 0.010845091 |
| *ZDHHC16* | 5.041189784 | 5.491737605 | 0.019850015 |
| *ZFAND4* | 5.519310556 | 3.620770144 | 0.015949278 |
| *ZGPAT* | 6.759435004 | 2.740442067 | 0.045198617 |
| *ZKSCAN1* | 7.159026274 | 8.559063213 | 0.025542644 |
| *ZKSCAN4* | 2.816842372 | 7.666812477 | 0.046182907 |
| *ZNF154* | 6.574189165 | 4.020731837 | 0.003491949 |
| *ZNF214* | 2.464034776 | 5.371720802 | 0.038257758 |
| *ZNF215* | 5.450116326 | 10.08190957 | 0.004186993 |
| *ZNF24* | 8.040823472 | 3.434048041 | 0.027350193 |
| *ZNF253* | 7.018359208 | 6.223507834 | 0.043718708 |
| *ZNF257* | 2.452454735 | 6.007819528 | 0.000224283 |
| *ZNF337* | 4.207658104 | 5.393650467 | 0.021644996 |
| *ZNF395* | 9.421630995 | 3.373957665 | 0.042317385 |
| *ZNF396* | 2.679648665 | 10.73713869 | 0.047168144 |
| *ZNF438* | 4.935060012 | 3.341424707 | 0.040461209 |
| *ZNF491* | 5.111883732 | 5.157726808 | 0.035977207 |
| *ZNF493* | 3.714957665 | 7.322650784 | 0.018007266 |
| *ZNF497* | 2.586460817 | 8.740954959 | 0.035258093 |
| *ZNF503* | 8.774416773 | 4.548945401 | 0.034496262 |
| *ZNF527* | 2.307965935 | 3.01404224 | 0.03379537 |
| *ZNF563* | 3.534194858 | 6.461869121 | 0.046580023 |
| *ZNF565* | 6.20219425 | 2.782542322 | 0.009293339 |
| *ZNF581* | 8.95669357 | 10.20250822 | 0.013471245 |
| *ZNF610* | 2.419805424 | 8.049337708 | 0.017501069 |
| *ZNF627* | 2.385900674 | 3.703630314 | 0.043464175 |
| *ZNF658* | 4.345718647 | 5.032439424 | 0.023411442 |
| *ZNF671* | 5.633900938 | 6.535237327 | 0.000891415 |
| *ZNF679* | 9.155338188 | 3.830707433 | 0.04636489 |
| *ZNF708* | 7.364916333 | 3.849093901 | 0.011909145 |
| *ZNF711* | 2.365414521 | 6.672096265 | 0.021812445 |
| *ZNF717* | 2.76182999 | 3.726313263 | 0.008052818 |
| *ZNF720* | 5.594565983 | 3.378896319 | 0.034875726 |
| *ZNF81* | 5.028296635 | 3.88157683 | 0.011077165 |
| *ZNF816-ZNF321P* | 2.513512085 | 6.373042243 | 0.019551545 |
| *ZNF850* | 5.912802206 | 6.7712173 | 0.029434172 |
| *ZNF98* | 2.364109497 | 6.614721939 | 0.003623938 |
| *ZNHIT6* | 2.294364119 | 5.789591191 | 0.022085568 |
| *ZSCAN12* | 5.589531224 | 4.417285012 | 0.035395295 |
