# Supplementary material for: Immune cell infiltration-related clinical diagnostic model for Ankylosing Spondylitis
Source: Front Genet. 2022 Sep 5;13:949882. doi: 10.3389/fgene.2022.949882 (PMC9575679; doi:10.3389/fgene.2022.949882)
Supplement: Supplementary file 14 [file Table4.DOCX]

**Supplement Table 4**

miRNA target genes

| ID | miRNA target |
| --- | --- |
| hsa-miR-449b-5p | *AAGAB; ACBD3; ACSL1; ADAM10; ADD2; ADIPOR2; ADK; ADO; AFF4; AGAP2; AGO4; AGTRAP; ALCAM; ALDOA; AMER2; ANK2; ANK3; ANKRD52; ANP32B; AP3S2; APH1A; AREG; ARHGAP1; ARHGAP26; ARHGAP36; ARHGAP44; ARID4A; ARSJ; ASIC2; ATG4B; ATG9A; ATMIN; ATP1A2; ATP5S; ATXN7; ATXN7L3B; AXL; B4GALT2; BCL2; BCL2L13; BMP3; BMP8B; BNC2; BRINP1; BRPF3; BTBD11; C12orf73; C1QL3; C22orf23; C2CD4A; C3orf58; C3orf70; C8orf37; C9orf69; CA7; CACNB1; CACNB3; CADM4; CALCR; CALN1; CAMK4; CAMSAP1; CAPN5; CAPN6; CASP2; CBFA2T3; CBFB; CCDC85A; CCDC88A; CCND1; CCNE2; CDC25A; CDC37; CDH4; CDIP1; CDK6; CDKN1C; CELF3; CERS6; CHD1; CHMP7; CHST12; CNTN2; CNTNAP1; CNTNAP2; COPS7B; CORO1C; CPLX2; CR2; CREB3L1; CRTC1; CSF1R; CTDSP2; CTDSPL; CTNND2; CTRC; CTTNBP2NL; CUEDC1; DAAM1; DAGLA; DCP1A; DCX; DDX17; DGKZ; DHRS13; DIXDC1; DLL1; DMWD; DNAJB1; DNAJC16; DNAJC24; DPYSL4; E2F3; E2F5; ELL2; ELMOD1; ELMSAN1; EML5; ERGIC1; ERN1; ESRRA; ESYT3; EVI5L; F2RL2; FAM117B; FAM126B; FAM133A; FAM167A; FAM175B; FAM46A; FAM73B; FAM76A; FAM81A; FBXO10; FBXO30; FGD6; FKBP1B; FKBP9; FLOT2; FNDC8; FOSB; FOSL1; FOXG1; FOXJ2; FOXP1; FOXP2; FRK; FRMD4A; FUT11; FUT8; FUT9; FXYD2; GABBR2; GABRA3; GALNT7; GAS1; GCH1; GHDC; GK5; GLRA3; GMNC; GNAI2; GNAO1; GNAQ; GOLPH3L; GORASP2; GPR12; GPR158; GPR22; GPR85; GREM2; GRIN2B; GRM7; HCN3; HDAC1; HECW2; HNF4A; HNF4G; HNRNPA1; HOXA13; HSPA1B; HTR2C; IGFBP3; IGSF1; IKZF1; IL6R; INA; INHBB; INPP5K; ITGB8; ITSN1; JAG1; JAKMIP1; KCND3; KCNH7; KCNK3; KIAA1210; KIAA1217; KIAA1462; KLF4; KLF7; LDHA; LEF1; LGI1; LGR4; LHX2; LHX9; LIMD2; LIN28A; LIN54; LMAN1; LMAN2L; LMBR1L; LPAR2; LRRC40; LRRC55; LRRC7; LRRTM2; LYPLAL1; MAP1A; MAP2K1; MAPT; MARCH8; MBD6; MCFD2; MCIDAS; MCTP1; MDM4; MET; METAP1; MGAT5B; MLLT3; MPP2; MPPED2; MRAS; MSANTD3-TMEFF1; MTA2; MTUS1; MYADM; MYCN; MYH9; MYOCD; NAA50; NAV1; NAV3; NCEH1; NCOA1; NDC1; NDST1; NETO1; NFE2L1; NMT2; NOS1AP; NOTCH1; NOTCH2; NPNT; NR4A2; NRIP3; NRN1; NRXN2; NUMBL; ORAI3; ORMDL3; OSGIN2; OXSR1; PACS1; PAG1; PALLD; PAN3; PARD6B; PARP15; PAX5; PDE4B; PDE7A; PDGFRA; PEA15; PEG10; PER2; PGF; PGM1; PGRMC2; PHF19; PIP5K1A; PKIA; PKP4; PLA2G15; PLAG1; PLEKHH2; PLOD1; PNOC; PODXL; POGZ; POMGNT1; POU2F1; POU3F3; PPARGC1B; PPM1L; PPP1R10; PPP1R11; PPP1R16B; PPP2R3A; PPP4R2; PREB; PRKACB; PRKD1; PROX1; PRPF38B; PRRG3; PTPN4; PTPRM; PVRL1; RAB21; RAB43; RAE1; RALGDS; RALGPS2; RALY; RANBP10; RAP1GAP; RAP1GDS1; RARB; RASGRP4; RCAN1; RDH11; RELN; RET; RGS17; RIC8B; RNF34; ROCK1; RPS6KA4; RPS6KL1; RRAGC; RRAS; RSPO4; RTF1; RTN4RL1; SAMD12; SATB1; SATB2; SCML2; SCN1A; SCN2B; SEMA4B; SEMA5B; SEPT3; SERPINF2; SGPP1; SGSM2; SGTA; SHISA7; SHOC2; SIDT1; SIPA1; SIX3; SLC12A2; SLC23A3; SLC25A27; SLC27A4; SLC30A3; SLC44A2; SLC4A5; SLC4A7; SLC6A1; SLC6A17; SLCO3A1; SMIM15; SMTNL2; SNAP25; SNTB2; SNX12; SNX15; SNX30; SOX4; SP2; SPCS2; SPRN; SPRY1; SRPR; SRR; STAC2; STK38L; STRN3; STX17; STX1A; SURF4; SVIP; SVOP; SYT1; SYT9; TAF4B; TAF5; TANC2; TANGO2; TBCK; TCF12; TGIF2; TM9SF3; TMCC3; TMED8; TMEFF1; TMEM109; TMEM126B; TMEM164; TMEM200B; TMEM246; TMEM25; TMEM251; TMEM52B; TMUB2; TNRC18; TOM1; TOX; TPD52; TRAFD1; TRANK1; TRIM41; TRIM67; TSN; TSPAN14; TSPAN18; TTC19; TUSC5; UBP1; UHRF2; UNC13C; UNC45A; USF1; VAMP2; VAT1; VPS37A; VPS37B; VPS4A; VWA5B2; WASF1; WDR91; WSCD2; WTAP; YY1; ZBTB46; ZC3H12B; ZC3H4; ZCCHC17; ZDHHC16; ZDHHC17; ZDHHC23; ZIC5; ZMYM4; ZNF275; ZNF281; ZNF282; ZNF304; ZNF579; ZNF641;* |
| hsa-miR-193b-3p | *AAK1; ADAMTSL3; AJUBA; ALKBH5; ANAPC15; ANKFY1; ANKRD13A; AP2M1; ARHGAP19; ARHGEF12; ARHGEF15; ARMC1; ATOH8; ATP5F1; ATXN1; BAK1; BAZ2A; BCL2L2; C18orf32; CALB1; CALM1; CAPRIN1; CBX7; CCND1; CD34; CLOCK; CLSTN1; CNBP; CNOT6; CTDSPL2; CXXC4; DAAM2; DBN1; DCAF7; DLEU7; DNAJC13; DYNLL2; E2F6; EBAG9; EN2; EPHA10; ERBB2IP; ERBB4; ETS1; ETV1; FAIM2; FAM131B; FAM84A; FAT4; FHDC1; FLI1; FOCAD; GDF11; GDPD5; GNAO1; GPR146; GPR27; GRB7; GREM1; GSG1L; GSR; HEG1; HELZ; HOXD13; HYI; IGFBP5; IL17RD; ING1; ING5; INO80; INO80D; IRF2BPL; JMY; KCNE1; KCNJ2; KDM5C; KIT; KLF7; KLHL2; KMT2E; KRAS; LAMC1; LAMC2; LAMP2; LRP4; LRRC8A; LUC7L3; MAP3K3; MCL1; MED14; MMP16; MMP19; MOCS2; MRPL43; MSANTD2; MYCN; MYLK; NOVA1; NRIP1; NSF; NT5DC3; NT5E; OSMR; PAK4; PCDH1; PET117; PLAG1; PLAU; PLXNC1; POLR2J2; PPARGC1A; PPP2R5C; PPTC7; PRR14L; PSEN1; PTBP1; PTEN; RFFL; RGL1; RGS9BP; RNF222; RPS21; RSF1; RUNX1T1; SCYL3; SEPN1; SF3B1; SIAH1; SKAP2; SLC10A6; SLC15A1; SLC16A6; SLC23A2; SLC30A9; SLC39A5; SNX27; SOS2; SOX5; SP8; SPECC1L; SPOPL; SRSF2; SRSF6; ST6GALNAC5; STMN1; STX16; SYT1; TAOK1; TBL1XR1; TBX20; TCEA2; TCF4; TESK2; TGFB2; TGFBR3; TMEM260; TMEM30A; TNFRSF21; TP53RK; TPM2; UBP1; WDR82; YWHAZ; ZC3H11A; ZFP41; ZFYVE26; ZNF248; ZNF385B; ZNF608; ZNF618;* |
| hsa-miR-330-5p | *AAMP; ABCC1; ALAD; ALOX15B; ANAPC16; ANKRD13B; AQP2; ARPC5L; ART4; ATP6V1G2; BHLHE40; C15orf32; C9orf24; CASKIN1; CBFA2T3; CD247; CD8A; CDH22; CEP85; CLU; CNBP; CNN1; CORO2B; CRY2; CTNS; CTRC; CUEDC2; DGCR6L; DNAJB12; DRD2; DYNLL1; EBPL; EFNA3; EIF1; ELFN1; ELK1; EMC8; EML2; ERBB2IP; FAM127B; FAM127C; FAM221B; FAM98A; FCGR3A; FCRLB; FGF11; FRA10AC1; FSCN1; FUCA1; GAB1; GLB1L; GLOD5; GOLGA7B; GPD2; GPI; GRIPAP1; GRPEL2; HOMER1; HOXC12; HSD11B1; HTR2C; IGLON5; KCNC1; KCNIP2; KCNQ4; KIAA1328; KIF17; KLF1; LDOC1; LETM2; LMNB2; LRRTM1; MAD2L1BP; MARCH10; MEST; METAP1; MEX3C; NCAN; NDNF; NKAIN1; NKX2-8; NRGN; NSL1; OTP; P2RY2; PABPC1L2A; PABPC1L2B; PALM; PARVA; PIP4K2C; PPP1R3F; PPP3CB; PRAF2; PTBP1; RALGAPA1; RALGPS2; RAP2A; RASL10B; RASSF1; RGL3; RIT1; SEPT4; SH3BP4; SIRPA; SLC19A2; SLC38A7; SLC7A2; SMAD6; SMTNL2; SNRPA; SPOCK1; SRPR; ST3GAL3; SYS1; SYT9; TLN1; TNFRSF11A; TNFSF14; TOMM34; TSPAN14; TSPAN18; UBXN10; UROC1; VAMP2; VLDLR; WARS2; WDR91; YBX2; ZBTB12; ZC2HC1A; ZNF322; ZNF394;* |
| hsa-miR-140-5p | *AARS; ADAM10; ADAM9; ADAMTS5; AFTPH; AKAP2; AKIRIN2; AMER2; ANKFY1; ANKRD12; ANO6; AP2B1; ARHGAP19; ARL15; BACH1; BAG2; BCL2L1; BMP2; C1R; C6orf47; CALU; CAMK2N1; CAND1; CAPN1; CCNYL1; CELF1; CELF2; CEP63; CERCAM; CORO2A; CREB3L1; CTSV; DNAJB4; DOK4; DPP10; DPYSL2; DTNA; EAF1; EGR2; EIF4G2; ELAVL2; EPB41L2; ERC2; ERLEC1; FAM102A; FAM105A; FAM175B; FAM214A; FBN1; FBXO45; FCHO2; FECH; FEN1; FES; FGF9; FKBP4; FXR1; GALNT16; GIT1; GLRX5; GNG12; GPD1L; GPR161; GUCD1; HAND2; HDAC7; HIAT1; HS2ST1; HSPA4L; IGFBP5; IPO7; JAG1; KAT2B; KATNBL1; KBTBD2; KIF1B; KLF6; KLF9; KLHL5; KLK10; LAMC1; LAMP2; LCOR; LHX2; LMAN1; LMNB1; LRAT; LRP4; LSM14B; MED13; MICAL3; MIER3; MINPP1; MIPOL1; MLIP; MMD; MNS1; MOB3A; MSMP; MTSS1; MYCBP2; MYO10; NAA20; NCKAP1; NCSTN; NDRG3; NFE2L2; NFYA; NOG; NPL; NUMBL; OGT; OSTM1; PABPC1L2A; PABPC1L2B; PALM2-AKAP2; PAX9; PDE7A; PDGFRA; PFN2; PGP; PIN1; PITX2; PPARGC1A; PPP1CC; PPTC7; PRRG1; PSRC1; PTP4A3; RAB10; RAB11A; RABIF; RALA; RAN; RAP1B; RAP2B; RASSF2; REST; RFFL; RGMB; RIC8B; RLIM; RNF19A; ROR1; SATB2; SCRN1; SEC22C; SEL1L; SEPT2; SIAH1; SLAIN1; SLC2A1; SLC39A9; SLC41A2; SMARCC2; SMOC2; SNX12; SNX2; SNX27; SOX4; SPRED1; SPRY1; SRCAP; STC2; STK3; STRADB; SYS1; TAF9B; TGFBR1; TIMM23; TLR4; TMEM115; TMEM123; TMEM189; TMEM189-UBE2V1; TMEM218; TMEM260; TMEM98; TMOD2; TPGS2; TRMT61B; TSC22D2; TSPAN12; TSSK2; TTK; TTYH2; UBE2V1; UBR5; UBXN7; UST; VCPIP1; VEGFA; VEZF1; WASF1; WDFY3; WEE1; WNK4; WNT9A; YES1; YOD1; YWHAG; ZBTB7A; ZNF800;* |
| hsa-miR-301a-3p | *ABCA1; ABCB7; ABHD3; ACBD3; ACBD5; ACER2; ACSL1; ACVR1; ADAM12; AFF3; AGFG1; AGO4; AGPAT3; AKAP1; ALX4; APCDD1; APPL1; AR; ARAP2; ARFIP1; ARHGAP1; ARHGAP12; ARHGAP24; ARHGEF26; ARHGEF4; ARID4B; ARRDC3; ARX; ATG16L1; ATG4D; ATP2B2; ATXN1; B4GALT5; BAG5; BAHD1; BARHL2; BBX; BHLHE40; BHLHE41; BMP3; BMPR2; BPTF; BTAF1; BTBD3; BTBD7; BTF3L4; BTG1; C16orf70; C4orf36; C5orf30; C7orf60; C9orf69; CALB1; CALM2; CAMSAP2; CAPRIN2; CAV2; CBFB; CCDC126; CCDC6; CCDC71L; CCDC85A; CCND3; CCNY; CCT6A; CD2AP; CD69; CDADC1; CDKN1A; CEBPE; CENPO; CEP170; CEP55; CHD5; CHIC1; CHMP3; CHMP4B; CHRM2; CHST1; CLCN5; CLCN6; CLIP1; CLTC; CMPK1; CNIH1; CNOT6; CNOT6L; CNOT7; COX7A2L; COX8C; CPEB1; CPEB2; CPEB3; CPEB4; CRISPLD1; CSNK1G1; CYB5R4; CYLD; CYP2U1; DCAF8; DCP2; DDX6; DENND1A; DEPDC1; DGKE; DLC1; DLG5; DLL1; DNAJC16; DOCK3; DPYSL2; DSEL; DYNLL2; E2F2; EDN1; EFR3A; EGLN3; EIF4E3; ELK3; EMX2; ENAH; ENPP5; ENPP6; EOGT; EPC2; EPHA7; EREG; ESCO2; ESR1; EXOC5; F3; FAM104A; FAM107B; FAM13A; FAM13B; FAM155A; FAM155B; FAM175B; FAM179B; FAM196B; FAM19A1; FAM43A; FAM46B; FAM73A; FAM73B; FANCA; FASTK; FBXO28; FBXO48; FBXO9; FBXW11; FERMT2; FGF10; FIBIN; FICD; FMR1; FNDC4; FOSL1; FOXF2; FRMD6; FRZB; FSTL5; FUT9; FXR1; FYCO1; FZD6; G3BP2; GABRB2; GADD45A; GADD45B; GAP43; GAREM; GJA1; GLRA3; GMFB; GMNC; GPATCH8; GRB10; GTF2H1; HABP4; HBP1; HECTD1; HECW2; HIAT1; HIVEP2; HOMER1; HOXA3; HOXA5; HOXB1; HOXB3; HOXC8; HOXD1; HPRT1; HRK; HS3ST5; HSPA8; IER3IP1; IGF1; IMPDH1; ING1; INHBA; INHBB; IQGAP2; IRF1; ITPKB; ITPR1; ITPRIPL2; JAKMIP1; JARID2; KALRN; KATNBL1; KBTBD8; KCNA4; KCNJ10; KCNJ15; KCNK10; KIAA0319L; KIAA1468; KIAA2022; KIF13A; KLF3; KLF7; KLHDC8A; KLHL20; KLHL3; KMT2C; LARP4B; LCLAT1; LCOR; LDLR; LDLRAD4; LMLN; LNPEP; LONRF1; LONRF3; LRCH1; LRIG1; LRP12; LRP1B; LRP8; LYSMD2; LZIC; MAF; MAFB; MAP3K12; MAP4; MAP7; MAPK1; MAPK6; MAPRE3; MAST2; MAT2B; MB21D2; MBNL1; MCTP1; MDFIC; MDM4; MEMO1; MEOX2; MET; MEX3D; MFSD6; MID1IP1; MIER1; MIER3; MLLT6; MMGT1; MPPED2; MSMO1; MTMR10; MTMR6; MXD1; MYB; MYBL1; MYO10; MYT1; NAA30; NAA50; NABP1; NAP1L3; NCKAP5; NCOA3; NEDD4L; NEUROD1; NEUROG1; NFIA; NFIB; NHLH2; NHSL1; NIPA2; NME7; NOL4; NPAT; NPEPL1; NPNT; NPTN; NPTX1; NR3C2; NRBF2; NRP1; NUDT10; OTUD3; OTX2; PAK6; PAN3; PAPD4; PAX6; PCGF5; PCNX; PDE7B; PDIK1L; PELI1; PEX5L; PFN2; PHAX; PHF14; PIGA; PIK3CB; PLAA; PLCL2; PLEKHF2; PLLP; PMEPA1; PNRC1; POU3F2; POU4F1; POU6F1; PPARG; PPFIA2; PPP6R1; PRKAA1; PRR15; PSAP; PSD; PTGES3; PTP4A1; PTPN4; PTPRG; PURG; PVRL3; PXK; QKI; QSER1; R3HDM1; RAB12; RAB1A; RAB30; RAB34; RAI2; RAP2C; RAPGEF4; RARB; RASD1; RASSF3; RB1CC1; RBBP8; RBM25; RBM33; RDX; REEP1; RFX7; RND2; RNF103-CHMP3; RNF11; RNF145; RNF165; RNF180; RNF38; ROCK2; RPA2; RPS6KA5; RRAGD; RTCA; RTN1; RUNX3; S1PR1; S1PR2; SASH1; SATB1; SATB2; SBF2; SECISBP2L; SERINC3; SESTD1; SETD7; SH3D19; SIX4; SKIDA1; SKP1; SLAIN1; SLC13A2; SLC24A2; SLC25A12; SLC25A32; SLC2A4RG; SLC35D1; SLC35F3; SLC44A1; SLC6A6; SLC8A1; SLC9A2; SMAD5; SMARCD2; SMOC1; SMOC2; SNAP25; SNIP1; SNPH; SNX2; SNX5; SOCS5; SOCS6; SOS2; SOWAHB; SOX4; SPEN; SPG20; SPHK2; SPOCK1; SPOPL; SPRED1; ST18; ST3GAL3; ST8SIA5; STC1; STIM2; STK33; STOX2; STT3B; STX6; SYBU; SYT6; TACC2; TAF4; TAF4B; TBC1D8; TBL1XR1; TBPL1; TES; TESK2; TGFA; TGFBR2; THOP1; TIMP2; TMEM110; TMEM159; TMEM170B; TMEM236; TMEM55A; TMEM55B; TMEM63B; TMOD1; TNF; TNFRSF1B; TNFSF10; TRERF1; TRIM3; TRIM37; TROVE2; TRPC3; TSC1; TSC22D1; TSHZ1; TSPAN18; TSPAN3; TTYH3; UBAP2L; UBB; UBC; UBE2D1; UBE3B; UBXN2B; UCP3; ULK2; UNC13A; USP28; USP47; USP48; USP8; UTS2B; UXS1; VGLL4; VMP1; VPS37A; WDR20; WDR47; WNT10A; WRN; WRNIP1; YTHDF2; ZBTB18; ZBTB4; ZCCHC14; ZFAND5; ZFP91; ZFPM2; ZFYVE9; ZIC5; ZMAT3; ZNF131; ZNF217; ZNF3; ZNF451; ZNF800; ZNF804B; ZPLD1;* |
| hsa-miR-140-3p | *ABCA1; ABHD2; ACBD5; ACTA1; ADA; ADAM10; ADAM17; ADD3; AEN; AHRR; AIDA; ALDH1A3; ANGEL1; AP2A2; ARHGAP12; ARHGAP18; ARID5B; ASB6; ASCC1; ATG4B; ATL2; ATP1B2; ATP2B1; ATP9A; B3GALNT1; BHLHE40; BSN; BTG1; C17orf49; C5orf30; CAP1; CAPRIN2; CASD1; CBL; CCDC179; CCNT2; CD274; CDC5L; CEP170; CHL1; CHRNA5; CHST14; CISD1; CNEP1R1; COA5; COL4A1; COLQ; CREBZF; CRK; CTSW; CUL2; CYB561; CYLC1; DAAM1; DAND5; DAZAP2; DBP; DCTN5; DDX11; DDX52; DLX5; DNAJB4; DNAJC1; DNAJC5; DTX4; DYRK1A; ECE1; EDEM1; EIF5A2; ELOVL1; EMC8; ERLIN2; EZH1; FAIM; FAM110D; FAM127B; FAM174B; FAM222B; FBXO33; FCGRT; FCRLB; FGD5; FGF9; FOXG1; FRMPD3; FRRS1; FXYD6; GAB2; GLT8D1; GOLPH3L; GOPC; GPATCH2; GRIA4; GRIK2; HBP1; HDC; HELLS; HGF; HLF; HMGCS1; HMGN3; HNRNPA2B1; HNRNPAB; HNRNPDL; HNRNPK; HOXA9; HOXB5; HS3ST2; HS3ST5; HSPH1; ILKAP; IQCJ-SCHIP1; IRF2; ISCA2; ITGA10; ITGA6; KCNB1; KCNK17; KCTD6; KDM2A; KDM3A; KDM5A; KHDRBS2; KHDRBS3; KIAA0232; KIAA0355; KIAA1147; KIAA1468; KIAA1549; KIAA2013; KIF1B; KLF4; LDLRAD4; LFNG; LHFPL2; LIMK2; LIPA; LPAR2; LRIG1; LTBP1; LYG2; MAFG; MANEA; MAP1LC3A; MARCKS; MARCKSL1; MBD6; MCTS1; MEIS2; MEMO1; MIA3; MINOS1-NBL1; MMRN2; MOB3B; MOCS2; MPZL1; MRFAP1; MTRNR2L5; MXI1; NAGA; NBL1; NCAN; NCL; NFYA; NHLH2; NKRF; NNAT; NOVA1; NR2F2; NR6A1; NRG3; NUP188; OSBPL10; OTUD7A; OTX2; P4HA1; PABPC1; PANK2; PAQR4; PARP2; PCDH8; PELI2; PET100; PIK3CB; PLEKHA2; PLEKHG3; POGZ; POMGNT1; POMT2; PPAP2B; PPFIA2; PPP1R10; PPP2R5E; PRKD1; PRPF38A; PRSS46; PTPRM; PVRL3; RAB23; RAI14; RALGPS2; RAP1B; RARB; RASGEF1B; RBFOX2; RBM12; RBM45; REEP5; RELT; RHOA; RIT2; RNF34; RNF7; RRAGC; RTN2; RTN4; S1PR2; SCHIP1; SDC4; SERAC1; SETD1A; SGIP1; SHC1; SIN3B; SIRPA; SIRT1; SIX5; SLC10A4; SLC17A9; SLC29A2; SLC30A3; SLC35F1; SLC6A9; SLITRK1; SMARCA2; SMCR8; SMUG1; SNAP25; SNCA; SNRNP27; SOBP; SP4; SPPL3; SPRED2; SRGAP3; SRPK1; SSR3; SVOP; TAB2; TADA3; TAF11; TAF4; TAX1BP3; TIGD3; TLL1; TMEM178B; TMEM229B; TMEM260; TNFAIP1; TNFSF12; TRIM28; TRIM39; TSTD2; TTBK1; TTC1; UBALD1; UBAP2L; UBXN10; UNC45A; UQCR10; USP1; USP34; VANGL2; VGLL2; VPS37C; WDR55; YWHAG; ZBTB38; ZC3H4; ZCCHC5; ZFPM2; ZNF131; ZNF146; ZNF24; ZNF777;* |
| hsa-miR-613 | *ABCB7; ACTB; ADAM12; ADPGK; AKAP12; ANKRD29; ANKRD34B; ANP32B; ANP32E; ANXA2; ANXA4; AP1S1; ARCN1; ARF3; ARG2; ARID2; ARPC1A; ARPC3; ASH2L; ASPH; ATF2; ATG13; ATP6V1A; ATXN1L; AXL; AZIN1; BAG4; BDNF; BET1; BLCAP; BPNT1; BRI3BP; BSCL2; C10orf126; C16orf47; C1GALT1; C2CD5; C2orf69; C5orf51; C9orf152; CA8; CAAP1; CALM2; CAPZA1; CASK; CBL; CCNC; CCSAP; CD164; CDC42; CDC42SE1; CDK14; CDK4; CDON; CEP44; CERS2; CHSY1; CITED2; CLCN3; CLTC; CNN3; COIL; COL4A3BP; CORO1C; CPEB1; CPED1; CPLX2; CREBL2; CT62; CTBP2; CTTNBP2NL; CXCL11; CXCR4; DCAF12L1; DDX5; DGKE; DGKH; DGKZ; DHX15; DLG4; E2F5; EAF1; EBPL; EDN1; EHMT2; EIF1AX; EIF4E; EIF4E3; ELF1; ELMO1; ELOF1; EPB41L4B; ERMP1; ETS1; FAM101B; FAM107B; FAM155A; FAM63B; FAM91A1; FBXL14; FBXO33; FBXW7; FGF17; FLI1; FN1; FNBP1L; FOSB; FUBP1; FZD7; G6PD; GAK; GCFC2; GCH1; GCLC; GJA1; GLCCI1; GLS; GPD2; GPR137C; GPR6; GTF2B; H3F3B; H3F3C; HACE1; HAND2; HCN1; HELZ2; HIAT1; HIGD1A; HMGN1; HMGN3; HNRNPA1; HNRNPA3; HNRNPK; HNRNPU; HOXB4; HS3ST3B1; HSP90B1; HSPD1; HSPE1; IGF1; ISY1-RAB43; JARID2; JUND; KALRN; KANK4; KCNA5; KCND3; KCNIP3; KCNJ2; KHSRP; KIF2A; KLF4; KMT2E; KRAS; KTN1; LASP1; LETM1; LIN7C; LMBR1; LRCH1; LRRC59; LRRC8A; MAB21L1; MAL2; MAP4K3; MAPK3; MATR3; MBLAC2; MCTP1; MECOM; MED1; MEOX2; METTL23; MIER1; MIPOL1; MMD; MMD2; MOB3C; MON2; MPP5; MPZ; MPZL1; MSANTD2; MTX1; MXD1; MXD4; MYEF2; MYLK; NANP; NAP1L5; NAV3; NCK2; NCL; NDFIP2; NDRG3; NET1; NETO1; NFATC2; NFATC3; NINJ1; NME1-NME2; NME2; NR1H3; NXT2; OSBPL7; OSTF1; OTUD5; OTX2; PAX3; PCDH17; PDCD10; PDE7A; PDGFA; PEA15; PEX26; PFN2; PGD; PHAX; PHF6; PHKG2; PICALM; PIGF; PIK3C2A; PIRT; PLEKHA1; PLEKHO2; POGK; POLR3G; PPIB; PPP4R2; PRKCE; PRKRIR; PTBP1; PTMA; PTPRG; PTPRS; RAB43; RAB5A; RABEPK; RABGAP1L; RAP1A; RAP1B; RARB; RASA1; RIT2; RNF138; RNF14; RNF141; RNF145; RRAGC; RRBP1; RSBN1; RSBN1L; RSPO3; RYBP; SDCBP; SEC23B; SEC62; SEC63; SEPT2; SERP1; SFPQ; SH3BGRL; SH3BGRL3; SKIDA1; SLC10A7; SLC25A22; SLC25A25; SLC25A30; SLC29A3; SLC35B3; SLC35F1; SLC44A1; SMAP1; SMARCB1; SMIM14; SNAI2; SNAP25; SNX2; SOX9; SP2; SPRED1; SRI; SRSF1; SRSF3; SRSF9; STARD7; STC2; SYN3; TACR1; TAGLN2; TAOK3; TBC1D15; TERF2; TEX30; TGIF1; TGIF2; THBS1; TIGD6; TIMP3; TKT; TMCC1; TMEM135; TMEM178A; TMEM243; TMEM55B; TMEM68; TMSB4X; TMX1; TNKS2; TNPO2; TPK1; TPM3; TPM4; TPPP; TRANK1; TRIM2; TSPAN4; TTR; TWF1; UBE2H; UBR5; UNC119B; USP33; UST; VAMP4; VASP; VPS45; WDR1; WDR48; WDR61; YPEL2; YRDC; YWHAQ; YWHAZ; ZBTB8A; ZC2HC1C; ZNF148; ZNF26; ZNF280C; ZNF281; ZNF580; ZNF800;* |
| hsa-miR-98-5p | *ABCB9; ABCC10; ABCC5; ABHD17C; ACAP3; ACER2; ACPP; ACSL6; ACVR1C; ADAM15; ADAMTS5; ADAMTS8; ADIPOR2; ADRB1; ADRB2; ADRB3; AEN; AFF2; AGO3; AGO4; AGPAT6; AGPAT9; AHCTF1; AIFM1; AKAP6; ALKBH1; AMER3; AMOT; AMT; ANGPTL2; ANKRD46; AP1S1; APBA1; APBB3; APPBP2; ARG2; ARHGAP20; ARHGAP28; ARHGEF15; ARHGEF38; ARHGEF7; ARID3A; ARID3B; ARL4D; ARL5A; ARL6IP6; ARRDC4; ASAP1; ATP2A2; ATP8B4; ATPAF1; ATXN7L3B; B3GNT7; BACE2; BACH1; BCAP29; BCAT1; BCL2L1; BCL7A; BEGAIN; BEND4; BIN3; BLOC1S5; BLOC1S6; BMP5; BRWD3; BSND; BTBD9; BTF3L4; BTG2; BZW1; BZW2; C11orf84; C15orf39; C15orf41; C19orf47; C20orf194; C2orf88; C3orf52; C5orf51; C8orf58; CACFD1; CACNB4; CACNG4; CADM2; CALM1; CALN1; CANT1; CAPN3; CASP3; CASP5; CBX2; CCDC144A; CCDC71L; CCL3; CCL3L1; CCL3L3; CCL7; CCNF; CCNJ; CCNJL; CCNY; CCR7; CCSAP; CD200R1; CD86; CDC25A; CDC34; CDC42SE1; CDCA8; CDH22; CDKN1A; CDV3; CEBPD; CECR6; CEP135; CEP164; CERCAM; CFL2; CGNL1; CHD7; CHRD; CHRNA7; CHSY3; CHUK; CLASP2; CLDN12; CLP1; CNTRL; COIL; COL14A1; COL15A1; COL1A1; COL1A2; COL24A1; COL27A1; COL4A1; COL4A2; COL4A6; COL9A1; COL9A3; CPA4; CPD; CPEB1; CPEB2; CPEB4; CPED1; CPSF4; CRCT1; CRK; CRTAP; CRY2; CSRNP3; CTDSPL2; CTNS; CTPS1; CXorf36; CYB561D1; CYP19A1; CYP46A1; DAGLA; DAPK1; DCAF15; DCUN1D2; DDI2; DDX19A; DDX19B; DDX26B; DHX57; DIAPH2; DKK3; DLGAP4; DLST; DMP1; DNAJB9; DPF2; DPH1; DPH3; DPP3; DPYSL3; DTX2; DTX4; DUSP1; DUSP16; DUSP22; DUSP4; DUSP9; DYRK1A; E2F2; E2F5; E2F6; EDEM3; EDN1; EEA1; EEF2K; EFHD2; EGLN2; EIF4G2; ELF4; ELOVL4; EMB; EOGT; EPHA3; EPHA4; ERCC4; ERGIC1; ESPL1; ETNK2; ETV3; FAM103A1; FAM118A; FAM135A; FAM189A1; FAM214B; FAM222B; FAS; FASLG; FBXO32; FIGN; FKRP; FNDC3A; FNIP1; FNIP2; FOXP2; FRMD4B; FSD2; FZD3; FZD4; GABRA6; GALC; GALE; GALNT1; GALNT15; GALNT2; GAN; GAS7; GATM; GCNT4; GDF6; GDPD1; GEMIN7; GHR; GIPC1; GK5; GLRX; GNAL; GNG5; GNPTAB; GOLGA4; GOLGA7; GPATCH3; GPCPD1; GPR132; GPR137; GPR156; GPR157; GPR26; GPR61; GPR63; GREB1L; GRID2IP; GRPEL2; GSG1L; GTF2I; GXYLT1; GYG2; HABP4; HAND1; HAS2; HBEGF; HDX; HECTD2; HIC1; HIC2; HIF3A; HMGA1; HMGA2; HOOK1; HOXA1; HOXB1; HOXC11; HOXD1; HS2ST1; HSPE1-MOB4; HTR1E; ICMT; ICOS; IDH2; IGDCC3; IGDCC4; IGF1R; IGF2BP1; IGF2BP2; IGF2BP3; IGLON5; IGSF1; IKBKAP; IKBKE; IL10; IL13; IMPG2; INPP5A; IQCB1; IRS2; ISLR; ITGB3; ITGB8; KCNC1; KCNC2; KCNJ11; KCNJ3; KCNQ4; KCTD10; KCTD16; KCTD17; KCTD21; KDM3A; KIAA1161; KIAA1958; KIAA2022; KIF2A; KLF9; KLHDC8B; KLHL13; KLHL23; KLHL31; KLK10; KPNA5; KREMEN1; LBH; LBR; LCOR; LDB1; LGR4; LIMD1; LIMD2; LIMK2; LIN28A; LIN28B; LIPH; LIPT2; LMX1A; LOR; LOXL4; LPGAT1; LRIG2; LRIG3; LRRC20; LRRC8B; LSM11; LUC7L3; LYVE1; MAP3K1; MAP3K13; MAP3K2; MAP4K3; MAPK6; MARCH9; MARS2; MBTPS2; MDM4; MED28; MED6; MED8; MEF2D; MEIS2; MESDC1; MEX3A; MFSD4; MGA; MIB1; MIEF1; MIOS; MLLT10; MMP11; MOB4; MSI2; MSN; MTUS1; MXD1; MYCBP; MYCN; MYO1F; NAA20; NAT8L; NDST2; NEFM; NEK3; NEK9; NGF; NHLRC2; NHLRC3; NID2; NIPAL4; NKD1; NKIRAS2; NME4; NME6; NOVA1; NPEPL1; NR6A1; NRAS; NRK; NSMCE2; NUP155; NXT2; ONECUT3; OSBPL3; OSMR; OSTF1; P2RX1; P4HA2; PALD1; PALM3; PANX2; PAPPA; PARD6B; PARM1; PARP8; PBX1; PBX2; PBX3; PCDH19; PCDH20; PCGF3; PCTP; PDE12; PDGFB; PDHB; PDPR; PEG10; PEX11B; PGRMC1; PI4K2B; PIAS4; PIGA; PIGU; PITPNM3; PKIA; PKN2; PLA2G15; PLA2G3; PLD3; PLEKHA8; PLEKHG7; PLEKHH1; PLEKHO1; PLXNC1; PLXND1; PMAIP1; POGZ; POLR2D; POTEG; POTEM; POU6F2; PPAPDC2; PPARGC1B; PPP1R16B; PPP2R2A; PPP3CA; PQLC2; PRDM1; PRDM5; PRKAA2; PRKAB2; PRKAR2A; PRPF38B; PRR18; PRRX1; PRSS22; PSORS1C2; PTAFR; PTPRD; PTPRU; PXMP4; PXT1; QARS; RAB11FIP4; RAB15; RAB40C; RAG1; RALB; RASGRP1; RASL10B; RB1; RBFOX1; RBFOX2; RBMS1; RCN1; RDH10; RDX; RFX6; RGAG1; RGS16; RGS17; RICTOR; RIOK3; RNF165; RNF20; RNF38; RNF44; RNF7; RNFT1; RORC; RPUSD2; RRM2; RRP1B; RSPO2; RTCA; RUFY3; RUNX1T1; S100A8; SALL3; SALL4; SAMD12; SBK1; SCD; SCN4B; SCYL3; SDK1; SEC14L1; SEC16B; SEC31B; SECISBP2L; SEMA4C; SEMA4G; SENP2; SENP5; SH2B3; SKIDA1; SKIL; SLC12A9; SLC16A10; SLC16A14; SLC1A4; SLC20A1; SLC25A18; SLC25A24; SLC25A27; SLC25A32; SLC25A4; SLC2A12; SLC30A1; SLC30A4; SLC30A6; SLC35D2; SLC4A4; SLC52A3; SLC5A6; SLC5A9; SLC6A1; SLC6A15; SLC6A5; SLC8A2; SLC9A9; SMARCAD1; SMC1A; SMCR8; SMIM3; SMUG1; SNAP23; SNN; SNX30; SOCS1; SOCS4; SOX13; SP8; SPIRE1; SPRED3; SPRYD4; SREK1IP1; SRGAP3; ST3GAL1; STAB2; STARD3NL; STEAP3; STK40; STOX2; STRBP; STX17; STX3; SUCLG2; SULF2; SURF4; SWT1; SYNCRIP; SYT1; SYT2; TAB2; TARBP2; TBKBP1; TBX5; TEAD3; TECPR2; TET3; TGDS; TGFBR1; TGFBR3; THAP9; THBS1; THRA; THRSP; TIMM17B; TMC7; TMEM110; TMEM143; TMEM2; TMEM234; TMEM255A; TMEM26; TMEM41A; TMEM65; TMOD2; TMPPE; TMPRSS2; TNFRSF1B; TNFSF9; TNIK; TP53; TRABD; TRANK1; TRIB1; TRIM17; TRIM41; TRIM71; TSC1; TSC22D2; TSPAN18; TSPEAR; TTC31; TTL; TTLL4; TXLNA; UBE2G2; UFM1; UGCG; UHRF2; ULK2; USP21; USP38; USP44; USP47; USP6; UTRN; VASH2; VAV3; VGLL3; VPS26B; VSNL1; VSTM5; WARS2; WASL; WDR37; WNK3; WNT9A; WNT9B; XK; XKR4; XKR7; XKR8; XRN1; YBX2; YOD1; YPEL2; YY1; ZBTB16; ZBTB26; ZBTB39; ZBTB5; ZBTB8B; ZC3H10; ZC3H3; ZC3HAV1L; ZCCHC3; ZFYVE26; ZNF10; ZNF200; ZNF202; ZNF275; ZNF280B; ZNF341; ZNF391; ZNF451; ZNF473; ZNF512B; ZNF516; ZNF583; ZNF641; ZNF689; ZNF697; ZNF70; ZNF710; ZNF774; ZNF784; ZNF879; ZPLD1;* |
| hsa-miR-196b-5p | *ABCB9; ABL1; ACER2; ACSL6; ANKRD49; ARHGAP28; ARHGEF38; BACH1; BCAT1; BIRC6; BLOC1S6; BNC2; C11orf84; C15orf41; CALM1; CALM3; CASK; CCDC39; CCNJ; CDC34; CDKN1B; CDYL; CHRD; COL14A1; COL1A1; COL1A2; CPD; CREBL2; DCAF15; DCDC2; DDX19A; DDX19B; DERL2; DIP2A; DIRC2; EBF1; EEF2K; ELAVL4; ELF4; ENTPD4; EPC2; EPHA7; EPS15; ERG; ERI2; ERLIN2; EXOC5; FAM102B; FAM127A; FAM127B; FLRT1; FNIP1; FOXP2; FRMD4B; GALC; GAN; GAS7; GATA6; GCNT4; GLTP; GPCPD1; GPR156; GRPEL2; GTF2A1; HABP4; HAND1; HMGA1; HMGA2; HOOK1; HOXA5; HOXA7; HOXA9; HOXB1; HOXB6; HOXB7; HOXB8; HOXC8; ICOS; IGF2BP1; IGF2BP3; ING5; IQCJ-SCHIP1; KCNJ2; KCNQ5; KLHL23; LCOR; LETMD1; LIN28A; LIN28B; LOR; LRIG2; LRIG3; LRP1B; LRRC17; LRRC4B; MAGT1; MAP3K1; MAP4K3; MAPK1; MARCH7; MARS2; MBNL2; NAP1L1; NCS1; NDUFC2-KCTD14; NME4; NR6A1; NRAS; NRXN1; NXPE3; OPCML; OSMR; OTX1; PACRGL; PANK2; PAPOLG; PARD6B; PAX7; PBX1; PBX3; PEG10; PIGU; PLEKHA8; POLR2D; POLR3D; POTEG; POTEM; PPAPDC2; PPP1R16B; PPP6R2; PRDM5; PSMD11; PTPRG; PVRL3; RASGRP1; RASSF3; RAVER2; RCC2; RDX; RGL2; RIOK3; ROCK1; RSPO2; RUFY3; RXFP2; SCHIP1; SDCBP; SEPT7; SERP1; SLC9A6; SMAD6; SNAP91; SOCS2; SOX12; SPTSSA; SSR1; STOX2; SUDS3; SYT9; TBPL1; TGFBR3; TMEM143; TMEM245; TMX1; TOX3; TRERF1; TSPAN12; TSPAN18; UBE2G2; UHRF2; VSNL1; XKR4; YIPF6; YOD1; ZBTB26; ZCCHC3; ZDHHC21; ZMYND11; ZNF385D; ZNF395; ZNF516; ZNF710; ZNF804B;* |
| hsa-miR-30b-5p | *ABHD6; ABI3BP; ABL1; ACTC1; ACTN1; ACTR3C; ACVR1; ADAM12; ADAM19; ADAM9; ADO; ADRA1D; ADRA2A; AKAP10; AMOTL2; ANKRA2; ANKRD17; ANKS4B; AP2A1; AP3S1; ARF4; ARID4A; ARL15; ARL4A; ARL6IP6; ASB3; ATF1; ATG5; ATL2; ATP8A1; AZIN1; B3GNT5; B4GALT6; BCL2L11; BCOR; BECN1; BEND7; BNC1; BTBD10; C10orf11; C10orf25; C4orf19; C7orf43; C8orf4; C9orf72; CA10; CACHD1; CALB2; CALCR; CALU; CAMK2D; CAMK2N1; CAMK2N2; CAPN5; CAPZA1; CARF; CARS; CAT; CBX2; CBX3; CCDC117; CCDC148; CCDC178; CCDC43; CCDC71L; CCNE2; CCNK; CCNT2; CCNY; CDC37L1; CDCA7; CELF3; CELF4; CEP76; CFDP1; CFL2; CHD1; CHL1; CHST1; CHST2; CNTN4; COL13A1; COL25A1; COL9A3; CPNE8; CPOX; CSAD; CSNK1A1; CTHRC1; CUL2; CYB561; CYP24A1; CYSLTR1; CYYR1; DBF4; DCBLD1; DDAH1; DDIT4; DESI2; DEXI; DGKZ; DIP2B; DLGAP1; DLGAP2; DLL4; DNAJC13; DNAJC25; DNAJC25-GNG10; DNMT3A; DOC2A; DOCK7; DOLPP1; E2F7; EAF1; EDEM3; EDNRA; EED; EFR3A; ELAVL2; ELAVL4; ELMOD2; ELOVL5; ENTHD2; EPB41; EPC1; EPC2; EPG5; ERG; ERLIN1; ESCO1; EVX2; EXTL2; FAM104A; FAM109B; FAM110B; FAM133A; FAM13C; FAM214A; FAM43A; FAM46A; FAM49A; FAM73B; FAM81A; FAM91A1; FANCF; FAP; FBXL14; FBXO45; FGF20; FKBP3; FOSL2; FOXB1; FOXD1; FOXG1; FRZB; FUCA1; FXR1; GABRA5; GABRB1; GALNT1; GALNT3; GALNT7; GALR1; GATA5; GATM; GCLC; GFPT2; GJA1; GLCCI1; GLDC; GLUD1; GLUD2; GMNC; GNA13; GNAI2; GNAO1; GNG10; GNPDA1; GOLGA1; GOLGA4; GPR150; GPR75-ASB3; GRAMD2; GRB10; GRIA2; GSKIP; GTF2H1; HEPHL1; HIVEP1; HLF; HLX; HMGB3; HOXA1; HOXA11; HOXB8; HTRA3; IDH1; IER5; IL1RAPL2; ING5; INO80D; INSIG2; IP6K3; IQCG; IRX4; ITGA4; ITGA6; ITGA8; ITSN1; JAK1; JAKMIP2; JARID2; JDP2; JPH4; KCNA4; KCTD8; KDM3A; KIAA0101; KIAA0226L; KIAA2026; KLF10; KLF11; KLHL20; KMT2C; KXD1; LARGE; LCLAT1; LGI1; LHX5; LHX8; LIN28A; LIN28B; LIN7C; LMBR1L; LRFN2; LRRC17; LRRC8D; LSM14B; LYN; LYSMD3; MAB21L1; MAML1; MAN1A2; MAP3K12; MAP3K5; MBNL3; MBOAT1; MBTPS2; METAP2; MEX3B; MFSD11; MIA3; MICAL1; MINOS1; MINPP1; MKRN3; MMD; MOV10; MSANTD3-TMEFF1; MSANTD4; MTDH; MYBL2; MZT1; NAALADL2; NAGPA; NDEL1; NECAP1; NEFM; NEUROD1; NEUROD6; NHLH2; NKAIN2; NKX2-2; NR4A2; NRBF2; NRBP1; NRXN3; NUS1; OMG; ORC2; OVOL1; OXR1; P4HA1; P4HA2; PAAF1; PAPD4; PARP16; PBRM1; PCDH20; PCGF5; PDCD10; PDCL; PDE5A; PDE7A; PDSS1; PEX5L; PFN2; PGM1; PGM3; PGP; PHACTR2; PI4K2B; PICALM; PIGA; PIGX; PIP4K2A; PIP4K2B; POLE3; POLR3G; PON2; POP1; PPID; PPIL3; PPP1R14C; PPP3CA; PPTC7; PRDM1; PRKRIR; PRPF40A; PSEN2; PSMD7; PTP4A1; PTPN13; PVRL3; R3HDM1; RAB15; RAB22A; RAB27B; RAB32; RAB38; RAB4B; RAB7A; RAB8A; RABGAP1L; RAD23B; RALGDS; RANBP9; RAP1B; RAP2B; RAPGEF4; RARB; RARG; RASA1; RASD1; RASEF; RCBTB1; REEP3; REV1; RFTN2; RFX2; RFX6; RHEBL1; RNF122; RNF157; RNF220; RNF34; RPRD1A; RRAD; RTCB; RTN4R; RUNDC3B; RUNX2; RWDD4; SCAF4; SCN1A; SCN9A; SCYL3; SDAD1; SEC22C; SEC23A; SEC61A2; SEMA3A; SEMA6B; SEPT7; SETD3; SH2B3; SH3RF1; SHOC2; SIX1; SIX4; SKP2; SLC22A5; SLC25A34; SLC29A3; SLC35A5; SLC35B4; SLC35D3; SLC35F1; SLC35F4; SLC41A2; SLC5A11; SLC6A9; SLC7A10; SMAD1; SMAP1; SMIM14; SNAI1; SNAPIN; SNX10; SNX16; SOCS1; SOCS3; SOCS6; SOX4; SOX9; SPHKAP; SRSF7; SSR3; STAC; STK39; STRIP1; STX2; SUCLG2; SURF4; SYNGR3; TAF4B; TASP1; TBC1D10B; TBPL1; TCF7; TEX2; TFDP1; TIA1; TMCC1; TMED2; TMEFF1; TMEM106B; TMEM121; TMEM170B; TMEM181; TMEM229A; TMEM87A; TMTC3; TNRC6A; TRAF3IP2; TRIM23; TRO; TRPM7; TTC39A; TTLL7; TTPA; TUSC3; TVP23B; TWF1; TXNDC5; UBAC1; UBE2D2; UBE2D3; UBE2F; UBE2I; UBE2J1; UBE2V2; UBE3C; UCP3; USP2; USP45; USP48; VAPA; VAT1L; VIM; VIP; VKORC1L1; VOPP1; VPS26B; WDR44; WDR82; WWP1; YBX1; YOD1; YPEL2; YPEL5; YTHDC1; YWHAZ; ZBTB11; ZBTB18; ZDHHC21; ZFAND5; ZFC3H1; ZNF200; ZNF280B; ZNF382; ZNF521; ZNF608; ZNF711; ZNRF1; ZPBP2;* |
| hsa-miR-494-3p | *ACBD4; ATXN3; B3GALT2; BBC3; BEND6; BHLHE40; CAMK1; CAV2; CKS1B; CLPTM1L; CYSTM1; DCP1B; DLGAP1; DNAJA2; EIF1; EIF1B; GTF3C6; GULP1; H3F3A; HIVEP1; JAKMIP2; JUND; KCTD14; KLRC4; LEFTY2; LUM; MORF4L2; MRPS23; MSANTD2; NHLH2; NMNAT2; NUTF2; PDCL3; PGPEP1L; PHOX2B; PLA2G12B; PPWD1; PRR14; PTEN; PTPN12; RAB5A; SLA2; SLC26A3; SLC35F5; SRSF7; TMEM135; UBP1; ZC3H7A;* |
| hsa-miR-186-5p | *ACSM2A; ACSM2B; ARAP2; ARCN1; BDH2; CXCL13; DSPP; DUS3L; EIF2D; FUT8; GPR37; HOXB2; INSM1; IRF8; MAPKAPK5; NIT2; PSPH; RHBDF2; SMIM15; SYCP1; TOB1; UBE2B; ZDHHC13; ZMYM6;* |
| hsa-miR-181c-5p | *ACTA2; ADAMTS5; ADO; ADRA1A; AFTPH; AK3; AKIRIN1; ANKRD13C; AP1AR; AP1S3; APOO; ARF6; ARL1; ARL5A; ARSJ; ASAH2B; ATP1B1; BAG4; BCL2; BCL2L11; BHLHE40; BLOC1S6; BRD1; BTBD3; C16orf87; C2CD5; C2orf69; CALM1; CALR; CARD11; CBLB; CBX4; CBX7; CCAR1; CCL8; CCNJ; CD69; CDON; CLASP1; CLVS1; CNTN4; COL16A1; CPNE2; CPOX; CREBRF; CRIM1; DDIT4; DDX52; DEK; DEPTOR; DERL1; DNAJA4; DNAJC13; DOCK7; DUSP10; DUSP6; E2F5; EIF4A2; ELAVL4; EPC2; ESM1; EVI2A; EXOSC3; FAM160A2; FAM222B; FAM3C; FBXO34; GATA6; GLB1L; GOT2; GPBP1; GPR137C; GSKIP; HEXIM1; HLF; HMBS; HMGB2; HOXA1; HOXA11; HOXB4; HOXB8; HOXD1; HS3ST3A1; HS6ST1; HSP90B1; IL1A; IPO8; IQCJ-SCHIP1; ITSN1; KANK1; KIAA0195; KLF15; KLF6; KLHL2; KMT2E; LCLAT1; LIF; LIN28A; LMO1; LMO3; LRBA; LRRC32; LRRC8D; LRRFIP1; LYRM1; MAMDC2; MAP2K1; MAP3K3; MARK1; MB21D2; MED8; MELK; METAP1; MKRN1; MLF1; MTPN; NAA50; NCALD; NFATC2IP; NKAIN2; NLN; NMT2; NPEPPS; NPTN; NR6A1; NUS1; OSBPL3; OTOGL; PAM; PAPD5; PARM1; PAWR; PAX9; PBX3; PCDHA1; PCDHA10; PCDHA11; PCDHA12; PCDHA13; PCDHA2; PCDHA3; PCDHA4; PCDHA5; PCDHA6; PCDHA7; PCDHA8; PCDHA9; PCDHAC1; PCNP; PDAP1; PDXDC1; PET117; PHLDA1; PHOX2B; PI4K2B; PKNOX2; PLCL2; PLEKHA3; PNISR; PNRC2; PPAP2B; PPFIA1; PRDM4; PRKCD; PROX2; PSPC1; PTBP2; RASSF6; RBAK; RBBP7; RLF; RNF182; RNF34; RSPO2; S1PR1; SCD; SCHIP1; SCOC; SEC24C; SEMA4G; SH2B3; SIN3B; SIRT1; SLA; SLC10A7; SLC25A37; SLC25A4; SLC2A3; SLC35E1; SMAP1; SMCO1; SNAI2; SNN; SPECC1L; SPP1; SPRY4; SRSF7; ST8SIA4; SYNPR; TBC1D4; TBPL1; TCERG1; TCF7L2; THBS4; TMED4; TMEM165; TMF1; TNF; TNFRSF11B; TRNP1; TSC22D2; TTC39A; TXNDC12; UBE2W; UBP1; UNC5A; USP42; WASF1; WDR82; WHAMM; WNK1; ZDHHC3; ZDHHC7; ZFAND6; ZFP14; ZFP30; ZFP36L1; ZFP62; ZFP82; ZIC2; ZIC3; ZNF136; ZNF140; ZNF200; ZNF23; ZNF266; ZNF268; ZNF283; ZNF286B; ZNF302; ZNF33B; ZNF470; ZNF527; ZNF562; ZNF594; ZNF667; ZNF699; ZNF780B; ZNF781; ZNF791; ZNF800;* |
| hsa-miR-495-3p | *ACTC1; ANKRD7; ASB5; BUB1; C17orf96; CAST; CMTM8; GPR22; HSDL2; HSPA5; ITM2B; KIAA0101; LHX2; MORF4L1; MTA3; NABP1; PLEKHH3; PSMC6; RAN; SNRPB2; SNRPF; TESC; UAP1;* |
| hsa-miR-425-5p | *ACTR3; AMPH; AP3M1; ATP5G3; B3GALT5; BCOR; C3orf17; CAB39L; CADM4; CFL2; CLVS2; CPEB1; CREBZF; CTSS; CUL4B; CXorf56; DHX40; DNAJC27; DYNC1I2; FOXD3; FOXJ3; FSCN1; FST; HSPB8; HSPH1; MAP2K6; MAP3K5; MAPK6; N4BP2L1; PABPN1; PCMT1; PDCD10; POMT2; PPM1F; PPP2CB; PTPRN2; RHNO1; RNF145; SCAMP1; SERP1; SH3RF1; SIAH2; SLC6A1; SNAP25; SPATS2L; SSX2IP; STMN1; TIMM10; TNC; UBQLN1; UXS1; WTAP; ZNF148; ZNF24; ZNF423; ZNF449;* |
| hsa-miR-365a-3p | *ACVR1; ADAM10; ADAMTS1; ADD3; ADM; AKT3; AMMECR1; ANK3; ANKRD11; ANKRD62; ANKS1A; ARRB2; ARRDC3; B4GALNT3; BCL2; BEST3; BMPER; BTF3L4; BTG2; C15orf32; C18orf25; C3orf38; C8orf44-SGK3; CDK9; CHD8; CHP1; CLCN6; COL7A1; CREB5; CRKL; CS; CSK; CXorf23; DCP2; DCUN1D5; DHFR; DLAT; DLX3; DMRTC2; DOCK4; DTNA; E2F2; EFEMP1; EHF; EIF4E3; ENPP5; ENTPD7; EPS8; ESRRA; ETS1; ETV1; EYA3; FAM60A; FAM91A1; GALNT4; GBA; GOSR1; GPC6; GRAMD1C; GRPEL2; GXYLT1; HAPLN2; HELLS; HHIP; HMGA2; HMGCR; HMGCS1; HOXA9; HS3ST1; HSPA8; IFNLR1; IMPA1; KCNA7; KCNH2; KCNJ2; KCNQ1; KIAA1147; KIAA1328; KIAA2026; KLF3; KLK15; KMT2E; LAMP2; LHX9; LIN7C; LPAR5; LSM14B; LYSMD3; MAFB; MAPK1IP1L; MAU2; MCOLN2; MEIS1; MEX3A; MGAT5; MMP3; MYCBP; MYLIP; MYLK; NABP1; NFIB; NKX2-1; NR3C2; NR4A2; ORMDL1; P2RY1; PAX6; PCNP; PIK3R3; PLEKHB2; POC1B-GALNT4; PPFIA2; PPP5C; PRDM1; PRKAR2A; PRKCI; PRPF40A; PTCHD1; PTTG1IP; QTRTD1; RAC1; RAPGEF4; RASD1; RGS9BP; RHEB; RICTOR; RNF152; RNF182; RNF44; S100A14; SALL1; SET; SGK1; SGK3; SGTB; SIAH3; SIX4; SLC16A2; SLC30A9; SNRK; SNX12; SNX24; SOCS5; SOCS7; SRGAP1; ST6GAL2; SYNJ1; TADA2A; TBK1; TECPR1; TFDP1; TIAM2; TLL2; TMEM183A; TMOD3; TRIM24; TRIM39; TSPYL6; UBP1; UBXN2B; UGCG; USP33; USP48; WDR37; WNT5A; WTAP; YTHDF2; YWHAH; ZNF148; ZNF385D; ZNRF1;* |
| hsa-miR-502-3p | *ADAM12; ADAMTS3; ADCY2; AHR; ANP32E; ATXN1; B4GALT5; BCL7A; CBLL1; CDH5; CDK6; CIT; CLDN11; CLIC4; COL10A1; COX7C; CSDE1; DCUN1D5; DOK6; ELAVL2; ESRRG; EVX2; EXOC5; FBN2; HMGCS1; HOXC11; HOXD10; ITGB3BP; KCNA1; KCTD9; KIAA0408; KPNA4; KRAS; LIN7C; LMX1A; MARCKS; MYCN; MYNN; NAP1L5; OGT; OLR1; PEG10; PHOX2B; POU3F1; PPP2R2C; PPP2R5E; PPP4R2; PSMD11; PTPRF; RAI14; RAP1A; RBMS1; RCC2; REEP5; RNF144A; RPRD1B; SAMD12; SCN2B; SEC63; SEMA3C; SHPRH; SLA2; SNX13; SPOCK1; STIM2; STRN; TARDBP; TMEM198; TSHZ3; TSPAN4; UBE2E2; UBE2H; ZBTB43; ZEB2; ZFHX4; ZMYM4; ZRANB2;* |
| hsa-miR-212-3p | *ADCY3; ADRA1A; AKIRIN1; ANGEL2; ANKRD29; ANP32A; ARFGAP2; ARHGEF11; ARID2; ARID4B; ARX; BOLL; BRI3; BTAF1; C11orf87; C16orf87; C19orf47; C1orf115; C8orf44-SGK3; CALU; CC2D1B; CDC40; CDK19; CFL2; CNIH1; CSDE1; CTGF; DAZAP2; DNAJA2; DOCK4; DPYSL3; DUSP9; DYNLL2; E2F5; EDIL3; EGR1; EIF4A2; ELMSAN1; EP300; ETV1; FAM167A; FAM91A1; FBXO21; FBXO28; FEM1C; FGF7; FKBP2; FOXA1; GAPVD1; GDF5; GEMIN6; GHR; GOLM1; GRM3; GRSF1; GTDC1; GTF2H1; H2AFZ; H3F3B; H3F3C; HAS2; HIP1R; HMGA2; HMGXB4; HNRNPU; HSD11B1; ISL1; KCNK2; KDM5B; KIAA1211L; KIAA1549; KLF7; LARGE; LEMD3; LIN28B; LSM11; LYN; MAP3K3; MAPK1; MAPK3; MED9; MEF2A; MEIS2; MIA3; MSANTD3-TMEFF1; MUC13; MYCBP2; NCALD; NDRG4; NET1; NMNAT2; NMT2; NOVA1; NREP; NTNG1; PAIP2; PCDH10; PDE7A; PEA15; PLK5; PNN; POC1B; PPM1G; PPP2CB; PPP2R5C; PSMD12; PTBP2; PYURF; RAB1A; RAP2B; RASA1; RB1; RGS7BP; RPP14; RTF1; SALL1; SAP30L; SCN1A; SEMA4G; SERP1; SGK3; SIRT1; SLAIN2; SLBP; SLC10A7; SLC25A28; SLC26A7; SLC2A1; SLC30A6; SLC6A1; SMAD2; SNIP1; SOCS2; SOX4; SOX5; SPAST; SPPL3; SPRED1; SPRY1; SPTSSA; SRGAP2; SS18; TCF15; TCF7L1; TIMM9; TJAP1; TMEFF1; TMEM43; TTK; USP38; USP9Y; WT1; WTAP; ZCCHC11;* |
| hsa-miR-203a-3p | *ADK; ANXA4; BANF1; C11orf91; C2orf80; C4orf33; C8orf4; CAMTA1; CCDC112; CCDC50; COPS7B; CSN2; CSRNP2; CTSS; DLX5; FGG; GLYATL2; GPATCH1; GPKOW; GRHL3; GSKIP; HNRNPL; HNRNPUL2; HOXB1; IFNA10; IFNA14; IL15; IL24; ISCA2; KIF2A; KRT26; KRT35; MLNR; MORF4L2; NCL; PDE6H; PRPS2; PTP4A1; RAB10; RNASE4; SCFD1; SCGB2A1; SH3BGR; SNAI2; SRA1; SUMO2; TEDDM1; TKTL1; TMEM100; TMEM69; TRAF3IP3; TTC39A;* |
| hsa-miR-374b-5p | *ADORA2B; APOPT1; ARL15; BOLA3; C19orf81; C5orf28; CCL2; CCL8; CDA; CEBPB; CYP26A1; EIF2S2; EN1; GADD45A; HSBP1; IL10; KCTD19; NCK1; NEUROG2; NFIL3; NKX2-2; NPPC; NTF3; OSTF1; PDE7B; PIGW; PITX2; PRR16; RGS14; SIX6; SLC15A4; SRSF7; STMN2; TCERG1; TMEM185A;* |
| hsa-miR-374a-5p | *ADORA2B; APOPT1; ARL15; BOLA3; C5orf28; CCL2; CCL8; CEBPB; CYP26A1; EN1; GADD45A; GNB2; GTF2A2; HSBP1; IL10; KCTD19; NCK1; NEUROG2; NFIL3; NKX2-2; NPPC; NTF3; OSTF1; PIGW; PITX2; PRR16; RGS14; SIX6; SRSF7; STMN2; TCERG1; TMEM185A;* |
| hsa-miR-532-3p | *AEBP2; ATP2B1; AZIN1; BET1; BZRAP1; C2orf68; C7; CACUL1; CALM3; CARHSP1; CBX1; CDC42BPG; CLMP; CNBP; CSF3; CT62; CTNNA2; CYB561D1; CYTH1; DDOST; EFNA5; EIF4G3; ETNK2; ETS1; ETV1; EXO5; FAM110D; FJX1; FOXO4; FOXR2; GCNT3; GPRC5A; HECTD1; HMGA2; HNRNPH3; LBH; LETMD1; MCAT; NEDD8; NPTN; PAPD5; PAX5; PDPN; PIK3CB; PPM1L; PPP1R2; PRMT6; PTP4A1; RAB9A; RAD51; SLC10A5; SNX24; SPARC; SSR3; STMN4; SUMO1; SYDE1; TCF23; TEAD3; TRHDE; UBE2D3; UBE2E1; WDFY3; WDR31; ZBTB7A; ZBTB7C; ZEB2; ZNF146; ZNF428; ZNF48; ZNF514; ZNF852;* |
| hsa-miR-760 | *AFAP1L2; AKAP3; ALS2; ANKRD11; ANP32B; AP4E1; ARNTL; ATXN1L; ATXN7; C10orf25; C14orf79; C16orf54; C20orf202; C6orf25; CAPN14; CASQ2; CD22; CDCA7L; CDH8; CDK5R1; CDX2; CHIT1; CLASP1; CLK3; CLPP; CSNK2A1; CTSG; CYB5B; DCTN2; DDX11; DERL2; DGKG; DOCK7; EBF2; EIF1; ELMSAN1; ELOVL6; ELP6; ENKD1; ENSA; EPB41L3; EPHB3; FABP3; FAM107B; FAM170B; FAM222B; FAM71B; FAM72A; FAM72B; FAM72C; FBXL3; FBXO8; FFAR4; FGF1; FNDC5; FOXA1; FOXC2; FREM2; FSHR; GALC; GIT1; GMEB2; GMPR; GOLGA7; GPR153; GPR3; GPR61; GPRC5C; GPX6; GRK5; GSDMA; GTDC1; GTF3C2; HBEGF; HES7; HIBADH; HIST1H1B; HIST1H1C; HIST1H1D; HIST1H1E; HIST1H2AA; HIST1H2AB; HIST1H2AD; HIST1H2AE; HIST1H2AH; HIST1H2AI; HIST1H2AK; HIST1H2AL; HIST1H2AM; HIST1H2BA; HIST1H2BB; HIST1H2BE; HIST1H2BG; HIST1H2BH; HIST1H2BL; HIST1H2BM; HIST1H2BO; HIST1H3A; HIST1H3B; HIST1H3C; HIST1H3D; HIST1H3E; HIST1H3F; HIST1H3H; HIST1H3I; HIST1H3J; HIST2H2AA3; HIST2H2AA4; HIST2H2BE; HIST2H3A; HIST2H3C; HIST2H3D; HIST3H2A; HIST3H2BB; HIST3H3; HOXA2; IL6; ISY1-RAB43; KCNA6; KCNA7; KXD1; LARS2; LMNB2; LRRC59; MAGED1; MBNL1; MED22; MKL2; MMP16; MYO18B; NCL; NECAP1; NGFR; NRAS; NRG2; OTUD7B; PABPC1L2A; PABPC1L2B; PHC2; PIGA; PIP4K2B; PIP4K2C; PPDPF; PPIP5K1; PPP1R37; PRMT6; PSMF1; PTP4A2; PYGO2; RAB27A; RASL10B; RBP2; RCBTB1; RNPS1; SCN2B; SCYL1; SEC24C; SEMA5B; SEPHS2; SEPT8; SH2D4A; SHCBP1; SLC25A34; SLC34A2; SLC6A1; SLC7A1; SLCO3A1; SNCB; SNN; STARD3; STARD7; STX3; SYNPO2L; TEAD2; TGFBI; TMEM11; TMEM184B; TMEM221; TNIP1; TOR3A; TRAM2; TSC22D3; UBE2K; UBE2L6; UBTD2; VMAC; ZDHHC9; ZNF79; ZSWIM6;* |
| hsa-miR-590-5p | *AIM1L; ALX1; AP1AR; ARHGAP24; ARMCX1; ASF1A; BCL7A; BEST3; BRWD1; C10orf12; CASKIN1; CCL1; CCL22; CD69; CHIC1; CPEB3; DMRTC1B; DUSP8; ELF2; ERG; FAM13A; FASLG; FGF18; GLCCI1; GLIS2; GRAMD3; IL12A; JAG1; KBTBD6; KBTBD7; KLF5; KRIT1; LANCL1; LRRC57; MAP2K3; MAP3K1; MATN2; MBLAC2; MSH2; MSX1; NFIB; NTF3; OSR1; PAN3; PBRM1; PCBP1; PCBP2; PCSK6; PDCD4; PDZD8; PELI1; PFKM; PITX2; PLEKHA1; PPP1R3A; RAB22A; RASGRP1; RECK; RMND5A; RNFT1; RSAD2; RTN4; S100A10; SCML2; SKI; SKP2; SLC16A10; SMAD7; SOX5; SPRY1; SPRY2; ST3GAL6; ST6GAL1; STAG2; TADA2A; TGFBI; TIMP3; TMEM170A; TRAPPC8; UBE2D3; XKR6; YOD1; ZFP36L2; ZNF367;* |
| hsa-miR-151a-3p | *AKT3; ARL17B; ATP2A2; C6orf106; CALD1; CASD1; CCDC67; CHL1; CLASP2; CLK1; CRK; CYP7B1; DECR1; DLC1; EFNA3; FAM104A; FSTL3; FXR1; GABRB1; GHR; HIF1A; LAMTOR3; MAF; MEX3C; NIPAL2; NPL; PANK2; PFN2; RFESD; RYBP; SIX1; SOCS5; TMEM98; TRA2B; TWIST1; UNC5B; UPP2; YTHDF3; ZFPM2; ZMAT1;* |
| hsa-miR-320b | *ALKBH5; ARFIP1; ARL8B; ARMCX2; ARPP19; ASH2L; BANP; BCAP29; CCR7; CD274; CDH20; CDK13; CNKSR2; COPS2; DAZAP1; DBN1; DDX42; DESI2; DHX15; DLX1; DPY30; EFS; EIF2B1; EMC7; EOGT; ETFA; FOXQ1; FTL; FUS; GNAI1; GRASP; HECTD2; HOXA10; INSM2; IRF6; KCNS3; KITLG; KLF13; KLF5; KLHDC9; KLHL36; LMO3; MDK; MED21; MSI2; MYL12A; NAA20; NABP1; NKX2-4; NPAS2; PAPD5; PAPOLA; PBX3; PCSK7; PHF1; PLEKHA5; PLK3; POLE4; POLR1C; PPCS; PRKAG2; PYGO2; RAC1; RAD51; RAI2; RAP1A; RASA1; RBM24; RCN2; RGS9BP; SCOC; SDHD; SFTA3; SLC10A3; SLC10A7; SLC28A3; SMARCD2; SMIM15; SPOPL; STARD4; STAT4; SYNGR2; TFRC; TIMM8B; TMEM106B; TMEM108; TPD52L2; TPM3; TRIAP1; TSC22D4; TUSC3; VDAC1; VIM; VPS37B; YWHAH;* |
| hsa-miR-532-5p | *APBB2; ATP2C1; BHLHB9; C11orf31; CAPN3; CCDC64; CCNG1; CCR4; CD40LG; CPEB3; CRIPT; CXCL1; CXCL2; CYCS; DDHD1; DENND6A; DHFR; ERCC6L; FGF14; GBP1; GBP3; GFRA1; GPR137C; HNRNPDL; HSPA9; IARS2; IL6ST; IRS2; KLHL7; KRAS; LEP; LINGO2; MCTP1; MDH2; MED1; MPP7; MRPL18; MTMR4; NAA50; NDP; NFATC2IP; NFIB; NKD1; NYAP2; PAK7; PCED1A; PSMD5; PSMD9; PURA; RAB18; RASSF5; RAVER2; RCOR1; SC5D; SESTD1; SLC25A14; SLC39A8; SMIM10; SRRM2; ST6GALNAC5; STC2; TAP1; TMUB2; TTC14; UBE2E3; ZDHHC13; ZKSCAN1; ZMPSTE24; ZNF250;* |
| hsa-miR-154-5p | *AQP9; ATG7; CADM2; CAMK2G; CDH20; COPS2; CUL2; DCAF16; DOCK1; E2F5; HDHD2; HNRNPR; JAM3; KCNA2; LYVE1; LZIC; MAP4; NPEPPS; NRXN3; PKNOX2; RAB5B; RAD9B; RBM7; RNF11; SALL1; SOS2; SRSF7; TADA1; TMEM108; TMEM133; TRPM7; UBE2D3; VPS4B; WAC;* |
| hsa-miR-320d | *ARFIP1; ARL8B; ARMCX2; ARPP19; ASH2L; BANP; BCAP29; CCR7; CD274; CDH20; CDK13; CNKSR2; COPS2; DAZAP1; DBN1; DDX42; DESI2; DHX15; DLX1; DPY30; EFS; EIF2B1; EMC7; EOGT; ETFA; FOXQ1; FTL; FUS; GNAI1; GRASP; HECTD2; HOXA10; INSM2; IRF6; KCNS3; KITLG; KLF13; KLF5; KLHDC9; KLHL36; LMO3; MDK; MED21; MSI2; MYL12A; NAA20; NABP1; NDRG3; NKX2-4; NPAS2; PAPD5; PAPOLA; PBX3; PCSK7; PFKM; PHF1; PLEKHA5; PLK3; POLE4; POLR1C; PPCS; PRKAG2; PYGO2; RAC1; RAD51; RAI2; RAP1A; RASA1; RBM24; RCN2; RGS9BP; SCOC; SDHD; SFTA3; SLC10A3; SLC10A7; SLC28A3; SMARCD2; SMIM15; SPOPL; STARD4; STAT4; SYNGR2; TFRC; TIMM8B; TMEM106B; TMEM108; TPD52L2; TPM3; TRIAP1; TSC22D4; TUSC3; VDAC1; VIM; VPS37B; YWHAH;* |
| hsa-miR-320a | *ARFIP1; ARL8B; ARMCX2; ARPP19; ASH2L; BANP; BCAP29; CCR7; CD274; CDH20; CDK13; CNKSR2; COPS2; DAZAP1; DBN1; DDX42; DESI2; DHX15; DLX1; DPY30; EFS; EIF2B1; EMC7; EOGT; ETFA; FOXQ1; FTL; FUS; GNAI1; GRASP; HECTD2; HOXA10; INSM2; IRF6; KCNS3; KITLG; KLF13; KLF5; KLHDC9; KLHL36; LMO3; MDK; MED21; MSI2; MYL12A; NAA20; NABP1; NKX2-4; NPAS2; PAPD5; PAPOLA; PBX3; PCSK7; PHF1; PLEKHA5; PLK3; POLE4; POLR1C; PPCS; PRKAG2; PYGO2; RAC1; RAD51; RAI2; RAP1A; RASA1; RBM24; RCN2; RGS9BP; SCOC; SDHD; SFTA3; SLC10A3; SLC10A7; SLC28A3; SMARCD2; SMIM15; SPOPL; STARD4; STAT4; SYNGR2; TFRC; TIMM8B; TMEM106B; TMEM108; TPD52L2; TPM3; TRIAP1; TSC22D4; TUSC3; VDAC1; VIM; VPS37B; YWHAH;* |
| hsa-miR-423-3p | *BCORL1; CINP; FAM222B; GBX2; KCTD2; LGALSL; MEIS1; MYO9A; PABPC1; RAP2C; VLDLR; WTIP; ZXDC;* |
