# Supplementary material for: Immune cell infiltration-related clinical diagnostic model for Ankylosing Spondylitis
Source: Front Genet. 2022 Sep 5;13:949882. doi: 10.3389/fgene.2022.949882 (PMC9575679; doi:10.3389/fgene.2022.949882)
Supplement: Supplementary file 15 [file Table6.DOCX]

**Supplement Table 6**

Expression of LYN in GSE73754 and GSE25101.

| GSE73754 | ExpLYN | GSE25101 | ExpLYN |
| --- | --- | --- | --- |
| GSM1902130_AS | 12.025606 | GSM616668_AS | 13.835624 |
| GSM1902131_AS | 12.264033 | GSM616669_AS | 13.522244 |
| GSM1902132_AS | 12.257301 | GSM616670_AS | 13.705671 |
| GSM1902133_AS | 11.879865 | GSM616671_AS | 13.584914 |
| GSM1902134_AS | 12.477696 | GSM616672_AS | 13.859509 |
| GSM1902135_AS | 11.913061 | GSM616673_AS | 13.660957 |
| GSM1902136_AS | 11.953071 | GSM616674_AS | 13.04604 |
| GSM1902137_AS | 12.129295 | GSM616675_AS | 13.169642 |
| GSM1902138_AS | 11.866295 | GSM616676_AS | 13.238595 |
| GSM1902139_AS | 11.679668 | GSM616677_AS | 13.415256 |
| GSM1902140_AS | 12.4431505 | GSM616678_AS | 13.387645 |
| GSM1902141_AS | 12.223888 | GSM616679_AS | 13.716332 |
| GSM1902142_AS | 12.4388 | GSM616680_AS | 13.777582 |
| GSM1902143_AS | 12.435083 | GSM616681_AS | 13.54682 |
| GSM1902144_AS | 12.245917 | GSM616682_AS | 12.931462 |
| GSM1902145_AS | 12.152135 | GSM616683_AS | 14.065189 |
| GSM1902146_AS | 12.028931 | GSM616684_nonAS | 13.842883 |
| GSM1902147_AS | 12.608322 | GSM616685_nonAS | 13.610257 |
| GSM1902148_AS | 11.993683 | GSM616686_nonAS | 14.009313 |
| GSM1902149_AS | 12.658246 | GSM616687_nonAS | 14.071702 |
| GSM1902150_AS | 12.4388 | GSM616688_nonAS | 13.327325 |
| GSM1902151_AS | 12.1406 | GSM616689_nonAS | 13.573433 |
| GSM1902152_AS | 11.886681 | GSM616690_nonAS | 14.021368 |
| GSM1902153_AS | 12.004857 | GSM616691_nonAS | 14.187391 |
| GSM1902154_AS | 12.205266 | GSM616692_nonAS | 13.558587 |
| GSM1902155_AS | 11.434575 | GSM616693_nonAS | 13.648062 |
| GSM1902156_AS | 12.6953335 | GSM616694_nonAS | 13.728303 |
| GSM1902157_AS | 12.348569 | GSM616695_nonAS | 13.759303 |
| GSM1902158_AS | 12.156234 | GSM616696_nonAS | 13.700793 |
| GSM1902159_AS | 12.420443 | GSM616697_nonAS | 13.683805 |
| GSM1902160_AS | 12.717997 | GSM616698_nonAS | 13.387645 |
| GSM1902161_AS | 12.210366 | GSM616699_nonAS | 13.765419 |
| GSM1902162_AS | 12.68654 |  |  |
| GSM1902163_AS | 12.133384 |  |  |
| GSM1902164_AS | 12.793337 |  |  |
| GSM1902165_AS | 12.011058 |  |  |
| GSM1902166_AS | 11.822081 |  |  |
| GSM1902167_AS | 11.722556 |  |  |
| GSM1902168_AS | 12.096219 |  |  |
| GSM1902169_AS | 12.055677 |  |  |
| GSM1902170_AS | 12.113488 |  |  |
| GSM1902171_AS | 12.412473 |  |  |
| GSM1902172_AS | 11.942188 |  |  |
| GSM1902173_AS | 11.639322 |  |  |
| GSM1902174_AS | 11.658934 |  |  |
| GSM1902175_AS | 11.788703 |  |  |
| GSM1902176_AS | 11.94536 |  |  |
| GSM1902177_AS | 12.218425 |  |  |
| GSM1902178_AS | 11.895759 |  |  |
| GSM1902179_AS | 11.751542 |  |  |
| GSM1902180_AS | 12.089736 |  |  |
| GSM1902181_AS | 12.052958 |  |  |
| GSM1902182_nonAS | 11.939486 |  |  |
| GSM1902183_nonAS | 11.963886 |  |  |
| GSM1902184_nonAS | 11.866295 |  |  |
| GSM1902185_nonAS | 11.898613 |  |  |
| GSM1902186_nonAS | 11.879865 |  |  |
| GSM1902187_nonAS | 11.920253 |  |  |
| GSM1902188_nonAS | 11.7067585 |  |  |
| GSM1902189_nonAS | 11.913061 |  |  |
| GSM1902190_nonAS | 11.314502 |  |  |
| GSM1902191_nonAS | 11.775458 |  |  |
| GSM1902192_nonAS | 11.879865 |  |  |
| GSM1902193_nonAS | 12.320374 |  |  |
| GSM1902194_nonAS | 12.506772 |  |  |
| GSM1902195_nonAS | 11.866295 |  |  |
| GSM1902196_nonAS | 11.856405 |  |  |
| GSM1902197_nonAS | 11.722556 |  |  |
| GSM1902198_nonAS | 12.01386 |  |  |
| GSM1902199_nonAS | 11.83745 |  |  |
| GSM1902200_nonAS | 12.1979065 |  |  |
| GSM1902201_nonAS | 11.806094 |  |  |
